# Platelet-derived serotonin epigenetically programs macrophage alternative activation to orchestrate type 2 airway inflammation

**DOI:** 10.1038/s41423-026-01455-2

**Published:** 2026-07-30

**Authors:** Ru Tang, Guofang Xia, Ying Zhu, Jinhong Shen, Zhihan Liu, Song Mao, Jiayao Zhou, Yuelong Gu, Shilei Pu, Zhipeng Li, Hai Lin, Congfeng Xu, Hongqing Xu, Feng Zhang, Yongxu Zhao, Shankai Yin, Weitian Zhang, Feng Liu

**Affiliations:** 1 Department of Otolaryngology Head and Neck Surgery, Otolaryngology Institute of Shanghai Jiao Tong University, Shanghai Sixth People’s Hospital Affiliated with Shanghai Jiao Tong University School of Medicine, Shanghai, China; 2 National Key Specialty of Skull Base Diseases Affiliated with Shanghai Sixth People’s Hospital, Shanghai, China; 3 Center for Sleep Monitoring and Respiratory Therapy Technology, Institute of Medical Equipment and Technology, Shanghai Jiao Tong University School of Medicine, Shanghai, China; 4 Department of Cardiology, Shanghai Sixth People’s Hospital Affiliated with Shanghai Jiao Tong University School of Medicine, Shanghai, China; 5 Department of Otolaryngology Head and Neck Surgery, Shanghai Children’s Hospital, Shanghai Jiao Tong University, Shanghai, China; 6 Department of Cardiovascular Medicine, Quzhou Affiliated Hospital of Wenzhou Medical University, Quzhou, China; 7 Department of Respiratory and Critical Care, Quzhou Key Laboratory of Aging Biology and Longevity Medicine, Zhejiang-Germany Joint Laboratory on Lung Injury Repair and Regeneration, Quzhou Affiliated Hospital of Wenzhou Medical University, Quzhou, China; 8 Shanghai Institute of Materia Medica, Chinese Academy of Sciences, Shanghai, China; 9 Shandong Laboratory of Yantai Drug Discovery, Bohai Rim Advanced Research Institute for Drug Discovery, Yantai, China

**Keywords:** TGM2, alternatively activated (M2) macrophages, serotonylation, type 2 inflammation, platelet, Chronic inflammation, Monocytes and macrophages

## Abstract

Type 2 inflammation (T2I) drives chronic airway diseases such as eosinophilic chronic rhinosinusitis (eCRS) and asthma, which are frequently accompanied by coagulation activation and platelet recruitment. However, whether and how these associated processes actively contribute to shaping inflammation remain largely unknown. Here, using single-cell transcriptomic profiling of human nasal polyp samples from eCRS patients with or without comorbid asthma, we identified a *TGM2*^hi^ macrophage population enriched in inflamed tissues and strongly correlated with local eosinophilia and systemic disease burden. Using dust mite-induced type 2 airway inflammation models in mice, we showed that the loss of *Tgm2*, either globally or in macrophages, selectively impaired alternative macrophage activation and attenuated both eosinophilic inflammation and epithelial remodeling. Mechanistically, TGM2 catalyzes the recently described histone modification H3Q5 serotonylation (H3Q5Ser), promoting an epigenetically permissive chromatin state for alternative macrophage activation. We further revealed a transcellular circuit driven by the activated platelet-derived monoamine metabolite serotonin (5-HT), which acts as a critical paracrine signal to fuel this epigenetic reprogramming of macrophages. Crucially, pharmacological inhibition of platelet 5-HT release or TGM2 activity ameliorated both nasal and pulmonary pathology in a mouse model of type 2 inflammation, underscoring the therapeutic potential of this pathway. Our findings establish a serotonin-TGM2-H3Q5Ser axis that couples platelet activation to macrophage epigenetic programming. This transcellular mechanism drives type 2 inflammation and reveals novel therapeutic opportunities across airway diseases.

## Introduction

Type 2 inflammation (T2I) is a key driver of several chronic mucosal diseases, including eosinophilic chronic rhinosinusitis (eCRS) and asthma [1–3]. These conditions, often triggered by exposure to allergens or microbial pathogens, involve core pathological features, such as eosinophil infiltration, epithelial barrier dysfunction, and extracellular matrix (ECM) remodeling, which are mediated primarily by Th2-associated cytokines, including IL-4, IL-5, and IL-13 [4, 5]. eCRS and asthma represent the upper and lower airway models of T2I, respectively. Their frequent clinical co-occurrence (eCRSwAS) is associated with greater disease severity and reduced therapeutic responsiveness, representing a heightened state of T2I across mucosal sites, providing a useful framework for dissecting the core mechanisms of type 2 immune pathology [6–8].

Macrophages play a central role in T2I in eCRS and asthma, serving as a critical nexus that integrates immune signals with the maintenance of tissue homeostasis. Specifically, alternatively activated (M2) macrophages (AAMs; traditionally termed M2) play a dual role in immune regulation and tissue repair [9–12]. Under Th2 cytokine stimulation, AAMs secrete IL-10 to resolve acute inflammation and promote tissue homeostasis while simultaneously driving pathological processes through the production of profibrotic mediators (TGF-β, IGF-1) and extracellular matrix components (e.g., FN1), as well as FXIII, which stabilizes ECM proteins via cross-linking [13–16]. This dual role allows AAMs to support tissue repair physiologically but exacerbates epithelial dysfunction, fibrosis, and tissue remodeling in chronic inflammatory conditions such as eCRS [17]. Moreover, AAMs amplify T2I by secreting chemokines and cytokines that recruit and activate eosinophils, Th2 cells, and group 2 innate lymphoid cells (ILC2s), thereby sustaining the inflammatory cascade and contributing to disease severity. Recent single-cell RNA sequencing studies have begun to reveal previously underappreciated heterogeneity within the AAM population, highlighting the existence of specialized subsets with distinct functional roles in T2I. For example, an *ALOX15*^+^ macrophage subset enriched in nasal polyps has been identified and correlated with disease severity [18]. Despite these advances, the upstream molecular mechanisms that drive AAM plasticity and lock them into a pathogenic state within the inflamed environment, particularly epigenetic regulation of their functions in orchestrating eosinophilic inflammation and driving tissue remodeling, remain poorly defined.

Beyond canonical inflammatory and remodeling responses, local activation of the coagulation system is a well-appreciated pathological hallmark of T2I [19] and is correlated with disease severity. This is characterized by profound fibrin deposition and elevated expression of coagulation factors, including FXIII, which has been established as a key driver of type 2 inflammatory pathology [20, 21]. However, how the coagulation system collaborates with inflammation, as well as the precise role orchestrated by its central cellular component, the platelet, remains largely unknown [22]. As a primary hub at the interface of coagulation and inflammation, platelets store potent signaling molecules, among which serotonin (5-HT) is a crucial circulatory factor that connects the neural, endocrine, and hemostatic systems [23]. Its pleiotropic functions extend to immune regulation [24–26], where it has emerged as a key modulator of T2I and a potential mediator of gut–lung/nose axis communication [27–29]. In addition to its classical receptor-mediated effects, 5-HT serves as a substrate for serotonylation, a transglutaminase (TGM)-mediated posttranslational modification that covalently conjugates 5-HT to glutamine residues on target proteins, including histone H3 at glutamine 5 (H3Q5Ser) [30]. Critically, this epigenetic modification has been shown to increase chromatin accessibility and govern pathological processes in contexts such as cancer and metabolic liver disease [31–34]. The central unresolved issue, therefore, is whether platelet-derived 5-HT utilizes this novel epigenetic pathway to drive macrophage-mediated pathology, thereby bridging systemic coagulation activation and localized type 2 inflammatory disease.

Our study revealed that platelet-derived serotonin fuels T2I by driving epigenetic reprogramming of macrophages via histone serotonylation. We identified TGM2 as the critical enzyme mediating this modification and demonstrated its necessity for alternative macrophage activation and inflammatory pathology in both human eCRS and mouse models. By linking systemic platelet activation to the epigenetic state of tissue macrophages, our work reveals a novel therapeutic axis for treating chronic airway inflammatory disease.

## Results

### *TGM2* emerges as a signature gene of AAMs in eCRS

**Fig. 1:**
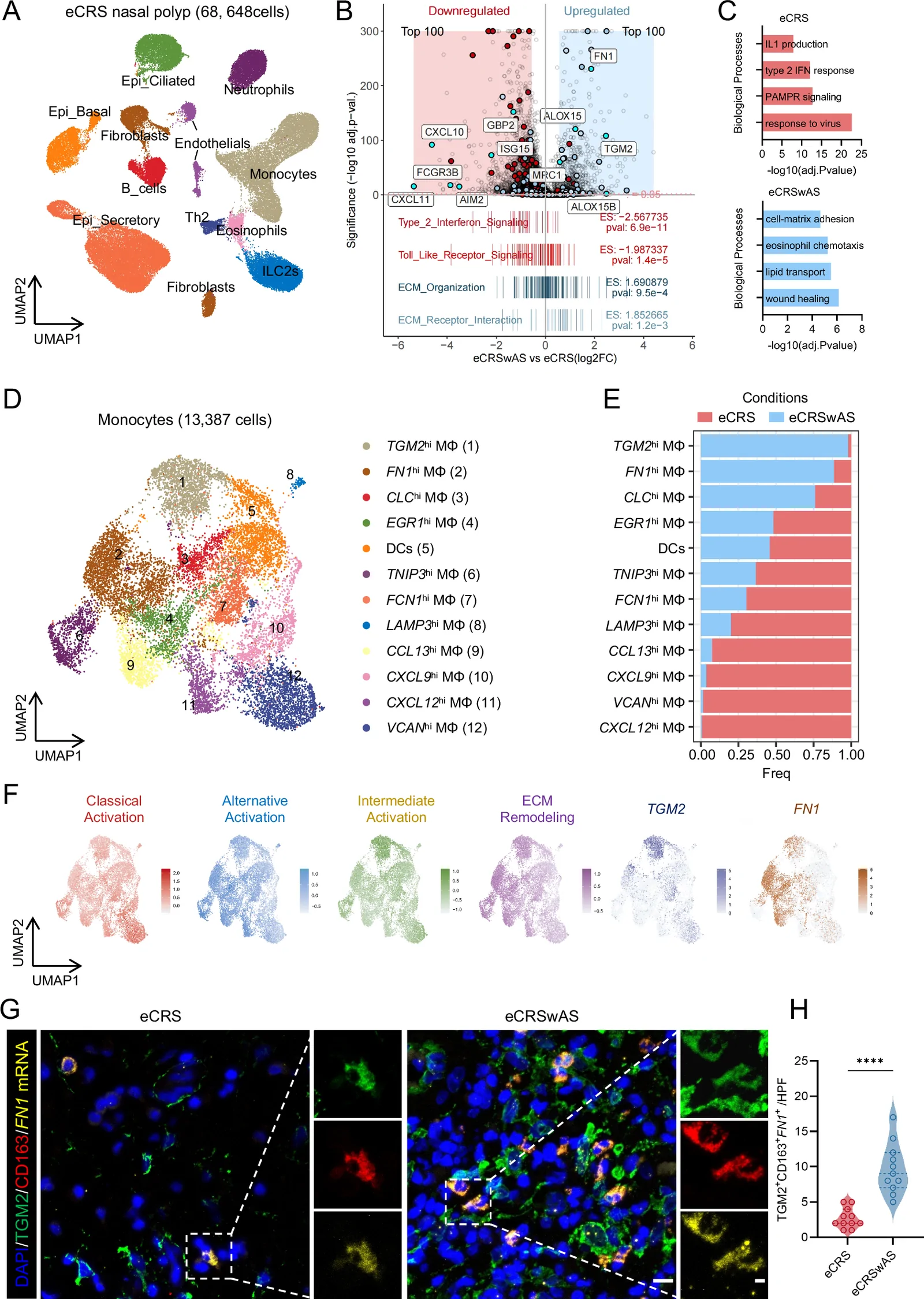
Single-cell transcriptomic analysis identifies *TGM2* as a signature gene in AAMs in eCRS. **A** Uniform manifold approximation and projection (UMAP) visualization of 68,648 cells from nasal polyp samples, showing 11 major cell types clustered by single-cell transcriptional profiles. Volcano plot **B** and GO analysis **C** of DEGs in monocytes between eCRS and eCRSwAS, with enriched gene set enrichment analysis (GSEA) terms for significant associations. **D** UMAP plot of 13,387 monocytes from eCRS and eCRSwAS patients, colored by 12 distinct clusters defined by their signature gene whose expression levels are significantly higher than the population average (i.e., *TGM2*^hi^ MΦ: avg_log2FC = 3.33, adjusted_*p*_value < 1 × 10^−300^; *FN1*^hi^ MΦ: avg_log2FC = 3.03, adjusted_*p*_value < 1 × 10^−300^). **E** Stacked bar plot showing the proportional distribution of cell types (eCRS vs. eCRSwAS) across monocyte clusters. **F** Feature plots visualizing the expression scores associated with signatures including classical activation, alternative activation, intermediate activation and ECM remodeling, as well as *TGM2* and *FN1* across monocytes in UMAP space. **G**, **H** Single-molecular fluorescence in situ hybridization (FISH) results revealed greater accumulation of *FN1* mRNA (yellow) in TGM2^+^ AAMs (indicated by CD163 (red) and TGM2 (green) staining) in nasal polyp samples from eCRSwAS patients than in those from eCRS controls. Scale bar: 10 µm

Since eCRS and its subtype with comorbid asthma (eCRSwAS) serve as effective models for studying T2I pathology, we performed single-cell RNA sequencing on 68,648 cells from nasal polyp samples from eCRS (*n* = 8) and eCRSwAS (*n* = 6) patients. Unsupervised clustering revealed eleven major cell types, namely, myeloid cells (including monocytes, neutrophils, and eosinophils), epithelial cells (basal, secretory, and ciliated), T cells (mainly the Th2 subset), B cells, endothelial cells, fibroblasts, and innate lymphoid cells (predominantly ILC2s) (Fig. 1A), with canonical marker expression validating cell identity (Supplementary Fig. S1A, B).

Differential gene expression analysis revealed striking differences in ILC2s, epithelial cells, and monocytes between eCRS and eCRSwAS samples. Given their potential role in disease pathogenesis, we focused our subsequent analysis on monocytes (Supplementary Fig. S2A). Specifically, monocytes from eCRSwAS patients exhibited upregulated expression of AAM features, such as *TGM2*, *FN1*, *MRC1*, and *ALOX15*, whereas their eCRS compartments presented elevated expression of classical activation markers, including *CXCL10*, *GBP2*, and *AIM2* (Fig. 1B and Supplementary Fig. S2B). Gene set enrichment analysis (GSEA) further revealed that ECM organization and ECM-receptor interaction pathways were enriched in eCRSwAS samples, whereas eCRS samples favored type 2 interferon (IFN) and Toll-like receptor (TLR) signaling signatures; this divergent pattern was consistently supported by Gene Ontology (GO) analysis (Fig. 1B, C and Supplementary Fig. S2C).

High-resolution subclustering of monocytes revealed twelve subpopulations, including one DC cluster, and eleven transcriptionally heterogeneous macrophage clusters spanning diverse functional states (Fig. 1D, F). The expression of AAM markers was significantly greater in two populations, *TGM2*^hi^ and *FN1*^hi^ macrophages, in eCRSwAS patients than in eCRS patients (Fig. 1E and Supplementary Fig. S2D), both of which were enriched for tissue remodeling signatures (Fig. 1F and Supplementary Fig. S3A, B). Although these two phenotypically distinct but spatially adjacent populations displayed partial transcriptomic overlap (e.g., *TGM2*^hi^ cells expressing low levels of *FN1;* Fig. 1F), pseudotime analysis suggested a convergent trajectory path toward a tissue remodeling fate (Supplementary Fig. S3C). Critically, to spatially localize and confirm the existence of this disease-relevant macrophage population, we employed single-molecule RNA fluorescence in situ hybridization (smFISH) integrated with multiplex immunohistochemistry (mIHC) and precisely determined that CD163^+^TGM2^+^ macrophages colocalized with high levels of *FN1* mRNA. These cells were significantly more abundant in eCRSwAS tissue samples than in eCRS tissue samples (Fig. 1G, H), thereby confirming the expansion of the *TGM2*^hi^ macrophage population with a strong tissue-remodeling signature in the most severe disease state.

Complementary evidence from human genetics revealed significant associations between *TGM2* polymorphisms and susceptibility to both CRS and asthma (Supplementary Fig. S3D), implicating *TGM2* in shared pathomechanisms of T2I diseases [35]. Thus, *TGM2* emerged as a key regulator, connecting eosinophilic inflammation and tissue remodeling *via* its elevated expression in eCRSwAS and its involvement in tissue remodeling and immunoregulatory processes. Coexpression analysis further confirmed that *TGM2* expression was significantly correlated with the expression of type 2 inflammatory markers, including *FN1*, *MRC1*, *CD163*, *ALOX15*, *TREM1*, *TREM2* and *IL10* (Supplementary Fig. S3E), indicating that *TGM2* serves as a critical signature gene for AAMs in eCRS.

To precisely delineate the transcriptional trajectory across the wider clinical spectrum of chronic rhinosinusitis, we integrated our local datasets with a comprehensive public scRNA-seq dataset (HRA000772) to construct a unified cohort spanning multiple disease subtypes, including healthy controls (HC), CRSsNP (chronic rhinosinusitis without nasal polyps), neCRSwNP (noneosinophilic chronic rhinosinusitis with nasal polyps), eCRSwNP (eosinophilic chronic rhinosinusitis with nasal polyps), and eCRSwNP with comorbid asthma (eCRSwNPwAS) (Supplementary Fig. S4A, B) [18]. The results strongly confirmed the robust enrichment of *TGM2* and *FN1* in the macrophage compartment, which was accompanied by the coexpression of *MRC1*, *ALOX15*, *CD163*, and *F13A1* (Supplementary Fig. S4C). Notably, rather than a discrete upregulation, the expression of both *TGM2* and *FN1* in macrophages exhibited a stepwise increase that perfectly mirrored the increase in eosinophilic inflammation and tissue remodeling severity across the disease spectrum, culminating in the highest levels within the eCRSwNPwAS group (Supplementary Fig. S4D), further underscoring their potential involvement in the pathogenesis of eCRS.

### *TGM2* expression is associated with disease severity

**Fig. 2:**
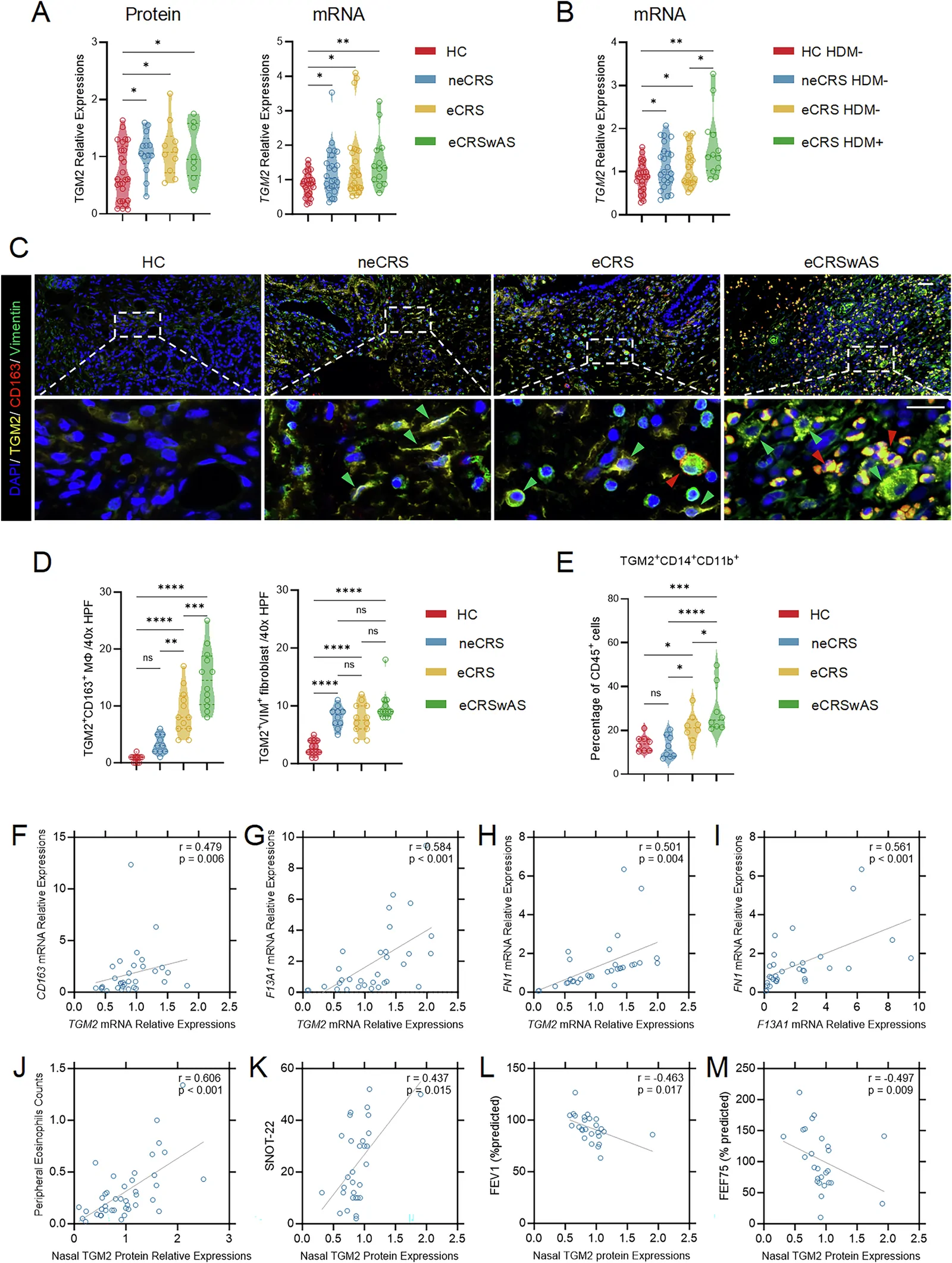
TGM2 expression is associated with disease severity. **A** Quantitative analysis revealed significantly higher *TGM2* expression in the eCRS groups than in the control group at both the protein (HC:25, neCRS:13, eCRS:10, eCRSwAS:8) and mRNA (HC:22, neCRS:24, eCRS:24, eCRSwAS:9) levels. **B** Compared with nonsensitized patients, house dust mite (HDM)-sensitized eCRS patients (*n* = 12) presented particularly elevated *TGM2* mRNA expression (*n* = 19). **C,** **D** Immunofluorescence staining showed increased TGM2 (yellow) expression in CD163^+^ macrophages (red, indicated by red arrows) from eCRSwAS patients. In contrast, Vimentin^+^ fibroblasts (green, indicated by green arrows) exhibited comparable TGM2 levels across neCRS, eCRS and eCRSwAS groups. Scale bar: upper (200 µm) and lower (100 µm). **E** Quantitative analysis by FCM validated a significantly expanded TGM2^+^ macrophage population in eosinophil-rich samples compared with that in controls. *TGM2* mRNA expression was positively correlated with the expression of AAM markers, including *CD163* (**F**; *n* = 32), *F13A1* (**G**; *n* = 32) and *FN1* (**H**; *n* = 32). (**i**) *FN1* mRNA expression positively correlated with *F13A1* mRNA expression (*n* = 32). **J** TGM2 protein levels were significantly positively correlated with peripheral eosinophil counts (*n* = 41). TGM2 protein expression positively correlated with 22-item Sinonasal Outcome Test (SNOT-22) symptom scores (**K**; *n* = 30) but negatively correlated with lung function parameters, including forced expiratory volume in 1 second (**L**; FEV1, *n* = 26) and forced expiratory flow at 75% (**M**; FEF75, *n* = 26), indicating an association with disease severity. The data are presented as the mean ± SEM. *p < 0.05, **p < 0.01, ***p < 0.001, ****p < 0.0001; ns, not significant (one-way ANOVA with Dunnett’s test for difference analysis, Spearman’s correlation analysis for correlation analysis)

After identifying *TGM2* as a key signature gene of AAMs in eCRS through our initial transcriptomic analysis, we next sought to validate its clinical relevance in a larger, well-characterized patient cohort. To this end, we analyzed nasal tissue samples from healthy controls (HC, *n* = 46) and from individuals with neCRS (*n* = 37), eCRS (*n* = 46), and eCRSwAS (*n* = 16) (Table E1). Our findings revealed significant upregulation of both *TGM2* mRNA and protein levels in CRS samples compared with those in healthy controls, with the most significant increases observed in the eCRS and eCRSwAS samples (Fig. 2A and Supplementary Fig. S5A). Notably, the highest levels of *TGM2* expression were detected in dust mite-exposed eCRS samples (Fig. 2B), suggesting a potential role for environmental allergens in driving *TGM2* expression.

To identify the cellular sources of TGM2 upregulation, we performed mIHC analysis of human nasal tissue samples and revealed that TGM2 was highly expressed in both macrophages and fibroblasts. Notably, its expression in macrophages increased significantly with eosinophilic inflammation, whereas in fibroblasts, TGM2 expression was elevated in CRS samples compared with that in healthy controls but did not further increase with disease severity (Fig. 2C, D). Flow cytometry analysis further confirmed that the TGM2^+^ macrophage population (CD45^+^CD14^+^TGM2^+^) was significantly greater in eosinophil-rich samples than in control samples, suggesting that these cells potentially contribute to disease development (Fig. 2E and Supplementary Fig. S5B).

Correlation analyses of nasal polyp tissues revealed that *TGM2* mRNA expression was specifically associated with a type 2 immune signature and strongly positively correlated with key AAM markers (e.g., *F13A1*, *FN1*, *CD163*, and *IL10*) but not with the type 1 marker *CD80* (Fig. 2F−H and Supplementary Fig. S5C, D). This AAM-related profile was reinforced by a strong positive correlation between *FN1* and *F13A1* (Fig. 2I). Clinically, TGM2 protein levels were strongly positively correlated with peripheral blood eosinophilia and worse symptom burden (SNOT-22 scores) (Fig. 2J, K). Furthermore, elevated TGM2 was linked to impaired objective respiratory function, which was significantly inversely correlated with FEV1, FEF75%, and FEF25-75% (Fig. 2L, M and Supplementary Fig. S5E). Collectively, these data suggest that TGM2 is a key driver linking T2I to functional airway decline in eCRS.

### *Tgm2* Shapes the inflammatory and fibrotic landscape of type 2 airways via macrophage eprogramming

**Fig. 3:**
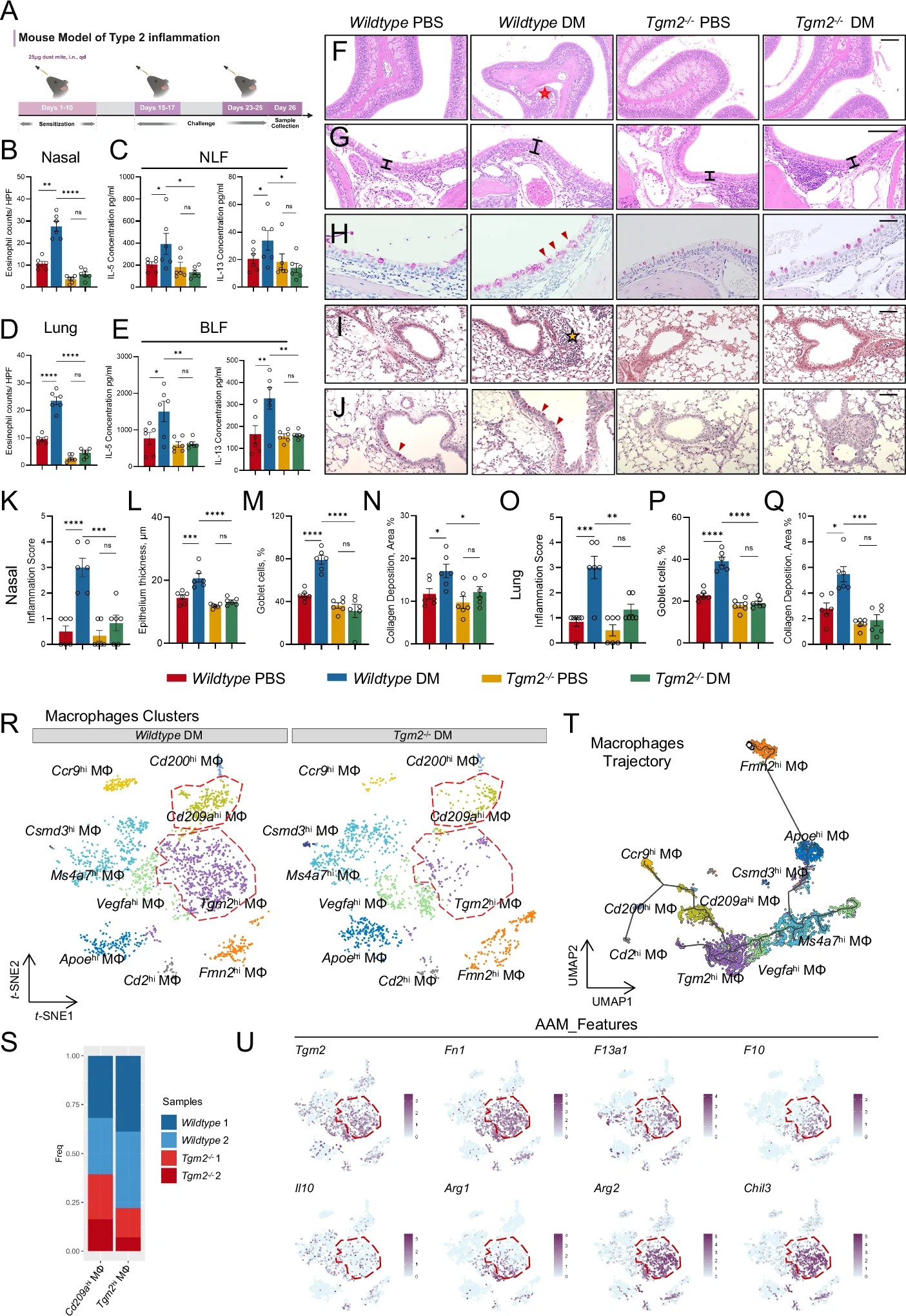
*Tgm2* is critical for type 2 inflammation in a dust mite-induced murine model. **A** Schematic diagram of the experimental design of the dust mite-induced type 2 inflammation model. **B** Comparative analysis of eosinophil infiltration in the nasal mucosa of dust mite-exposed wild-type mice and *Tgm2*^−/−^ mice. **C** Measurement of interleukin-5 (IL-5) and interleukin-13 (IL-13) concentrations in nasal lavage fluid (NLF) from wild-type and *Tgm2*^−/−^ mice after dust mite challenge. **D** Comparative analysis of eosinophil infiltration in lung tissues from dust mite-exposed wild-type and *Tgm2*^−/−^ mice. **E** Measurement of interleukin-5 (IL-5) and interleukin-13 (IL-13) concentrations in bronchoalveolar lavage fluid (BLF) from dust mite-exposed wild-type versus *Tgm2*^−/−^ mice. Hematoxylin‒eosin (HE) staining of nasal mucosa from dust mite-exposed wild-type and *Tgm2*^−/−^ mice revealed subepithelial inflammation (**F**, indicated by a red five-pointed star) and epithelial thickness (**G**, indicated by an I-bar). **H** Periodic acid–Schiff (PAS) staining of nasal mucosa reveals goblet cell hyperplasia (indicated by a red arrowhead) as intensely magenta-stained epithelial cells. HE **I** and PAS **J** staining revealed airway inflammation (indicated by yellow five-pointed stars) and goblet cell hyperplasia (indicated by red arrowheads) in lung tissue. Scale bar: 100 μm. Quantitative comparison of inflammation scores, goblet cell hyperplasia and collagen deposition in nasal mucosa **K**–**N** and lung tissues **O**–**Q** between dust mite-treated wild-type and *Tgm2*^−/−^ mice. **R** *t*-SNE plot of 21,808 macrophages from the nasal mucosa of dust mite-treated wild-type and *Tgm2*^−/−^ mice, colored according to 10 distinct macrophage clusters and separated by genotype, with red circles highlighting the most prominent *Tgm2*^hi^ macrophage population. (i.e., *Tgm2*^hi^ MΦ: avg_log2FC = 2.18, adjusted_*p*_value = 7.5×10^-179^; *Cd209a*^hi^ MΦ: avg_log2FC = 5.45, adjusted_*p*_value = 1.0×10^-273^). **S** Quantitative comparison of the proportions of *Tgm2*^hi^ and *Cd209a*^hi^ macrophages in the nasal mucosa between dust mite-exposed wild-type and *Tgm2*^−/−^ mice. **T** Pseudotime trajectory analysis illustrating the differential activation pathways of macrophages in dust mite-induced nasal mucosa. **U** Feature plots showing the expression patterns of *Tgm2* and other indicated AAM markers (including *Fn1*, *F13a1*, *F10*, *Il10*, *Arg1*, *Arg2*, and *Chil3*) across macrophage populations in the *t*-SNE dimensional space, with red circles highlighting the most prominent *Tgm2*^hi^ macrophage population. The data are presented as the mean ± SEM. *p < 0.05, **p < 0.01, ***p < 0.001, **p < 0.0001; ns, not significant (two-tailed unpaired *t* test and one-way ANOVA with Dunnett’s test)

To investigate the role of *Tgm2* in type 2 airway inflammation, we subjected systemic *Tgm2*^−/−^ mice and their wild-type littermate controls to a house dust mite exposure model (Fig. 3A). TGM2 expression was increased in the nasal and lung tissues of the dust mite-exposed wild-type mice (Supplementary Fig. S6A), and histochemical analysis revealed a significant increase in the number of TGM2^+^ macrophages in these tissues (Supplementary Fig. S6B, C).

Strikingly, *Tgm2*^−/−^ mice exhibited markedly attenuated T2I. In the nasal compartment, we observed significantly reduced eosinophil infiltration and lower levels of IL-5 and IL-13 in nasal lavage fluid (NLF) (Fig. 3B, C and Supplementary Fig. S6D) and improved tissue pathology, including diminished epithelial thickening, goblet cell hyperplasia, and collagen deposition (Fig. 3F–H, K–N and Supplementary Fig. S6F). This protective effect extended to the lungs, with *Tgm2*^*−/−*^ mice showing decreased eosinophil accumulation, reduced levels of IL-5 and IL-13 in the bronchoalveolar lavage fluid (BLF) (Fig. 3D, E), and alleviated airway goblet cell metaplasia and pulmonary fibrosis (Fig. 3I, J, O–Q and Supplementary Fig. S6E, G). Collectively, these data demonstrate that *Tgm2* deficiency broadly suppresses type 2 inflammatory responses and associated tissue remodeling throughout the respiratory tract.

To investigate the cellular mechanisms underlying the attenuated inflammation in *Tgm2*^*−/−*^ mice, we performed scRNA-seq profiling on nasal mucosa from dust mite-exposed wild-type and *Tgm2*^−/−^ mice, which included a total of 64,662 cells, which were classified into 10 distinct cell types, including macrophages, neutrophils, eosinophils, T cells, B cells, epithelial cells, and fibroblasts (Supplementary Fig. S6H). Following *Tgm2* deletion, we observed a marked reduction in the proportions of neutrophils, eosinophils, and macrophages, which was consistent with the decreased inflammatory response in the *Tgm2*^*−/−*^ mice (Supplementary Fig. S6I). Further analysis revealed a distinct *Tgm2*^hi^ macrophage population characterized by high expression of *Tgm2* alongside canonical AAM markers (*Arg1*, *Arg2*, and *Chil3*) and ECM-remodeling effectors (*Fn1*, *F13a1*, and *F10*). Notably, this *Tgm2*^hi^ population exhibited a conserved transcriptional signature with that of human *TGM2*^hi^ and *FN1*^hi^ macrophages, highlighting a cross-species AAM state dedicated to tissue remodeling. In response to *Tgm2* deficiency, both *Tgm2*^hi^ and *Cd209a*^hi^ macrophages were markedly depleted, with the *Tgm2*^hi^ population being particularly severely affected (Fig. 3R–U and Supplementary Fig. S6J, K).

**Fig. 4:**
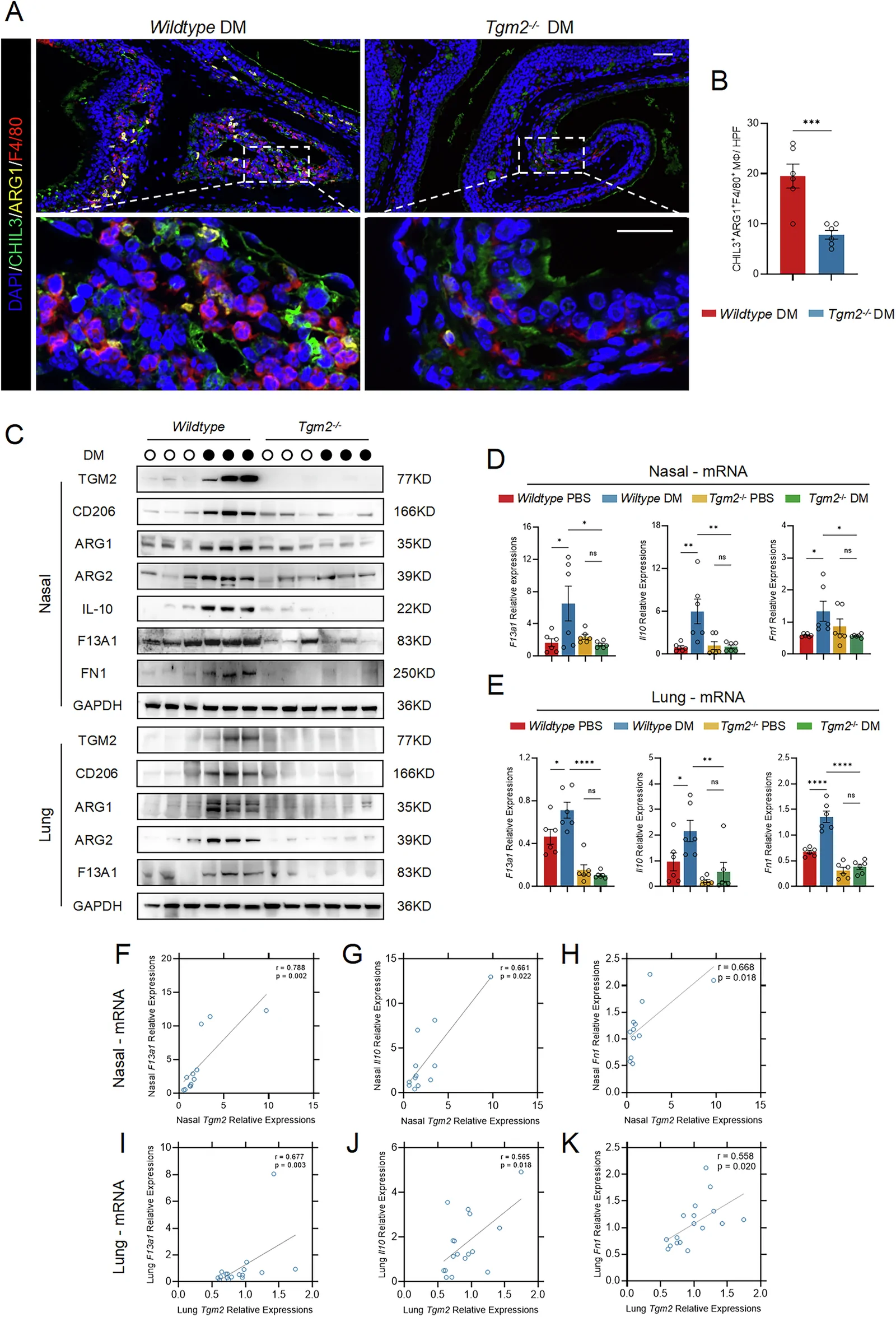
*Tgm2* is essential for dust mite-induced alternative activation of macrophages. **A**, **B** Multicolor immunofluorescence staining demonstrated colocalization of the macrophage markers F4/80 (red), ARG1 (yellow), and CHIL3 (green) in the nasal mucosa of dust mite-treated wild-type mice compared with that in *Tgm2*^−/−^ mice, with decreased AAMs in knockout mice. Scale bar: 100 μm. **C** Western blot analysis confirmed the reduced protein expression of canonical AAM markers (CD206, FN1, F13A1, IL-10, ARG1, and ARG2) in both the nasal mucosa and lung tissues of dust mite-challenged *Tgm2*^−/−^ mice compared with wild-type control mice. Quantitative RT‒PCR revealed significantly decreased mRNA expression of the profibrotic factors *Fn1*, *Il10*, and *F13a1* in both the nasal mucosa **D** and lung tissue **E** of *Tgm2*^−/−^ mice following dust mite exposure. Scatter plots with Spearman correlation coefficients demonstrating strong positive correlations between *Tgm2* expression levels and *Fn1* (**F**: nasal mucosa *r* = 0.668; **I**: lung *r* = 0.558), *F13a1* (**G**: nasal mucosa *r* = 0.788; **J**: lung *r* = 0.677) and *Il10* (**H**: nasal mucosa *r* = 0.661; **K**: lung *r* = 0.565) across all dust mite-treated samples. The data are presented as the mean ± SEM. *p < 0.05, **p < 0.01, ***p < 0.001, ****p < 0.0001; ns, not significant (one-way ANOVA with Dunnett’s test for difference analysis, Spearman’s correlation analysis for correlation analysis)

On the basis of the scRNA-seq findings that revealed *Arg1* and *Chil3* as highly expressed markers in the *Tgm2*^hi^ macrophage population (Fig. 3U), we next sought to visualize and quantify the loss of this specific subset in situ. mIHC staining for ARG1 revealed a marked reduction in the number of these cells in the tissues of *Tgm2*^−/−^ mice following dust mite challenge (Fig. 4A, B). Western blot analysis further demonstrated markedly lower protein levels of AAM markers, including FN1, F13A1, ARG1, ARG2, CD206, and IL-10, in both the nasal and lung tissues of the *Tgm2*^−/−^ mice than in those of their wild-type littermates (Fig. 4C and Supplementary Fig. S7A, B). Consistent with these findings, the results of the qPCR analysis confirmed that the mRNA expression of *Fn1*, *F13a1*, and *Il10* was significantly lower in the *Tgm2*^*−/−*^ tissue samples than in the WT tissue samples (Fig. 4D, E), with the expression of these genes strongly correlated with the abundance of *Tgm2* across the wild-type tissue samples (Fig. 4F–K). Notably, whereas scRNA-seq did not detect significant transcriptional changes in *Il10*, *Igf1*, or *Ccl24*, quantitative ELISA revealed substantially reduced protein concentrations of IL-10, IGF-1, and CCL-24 in *Tgm2*^*−/−*^ samples, confirming a systematic impairment in type 2 inflammatory factor production (Supplementary Fig. S7C). Collectively, these multiomics data identify *Tgm2* as a master regulator of alternative macrophage activation, orchestrating ECM remodeling, immunosuppressive cytokine production, and effector cell recruitment, which together drive T2I pathology across both the upper and lower airways.

### TGM2 is essential for alternative activation of macrophages and AAM-driven type 2 airway inflammation

**Fig. 5:**
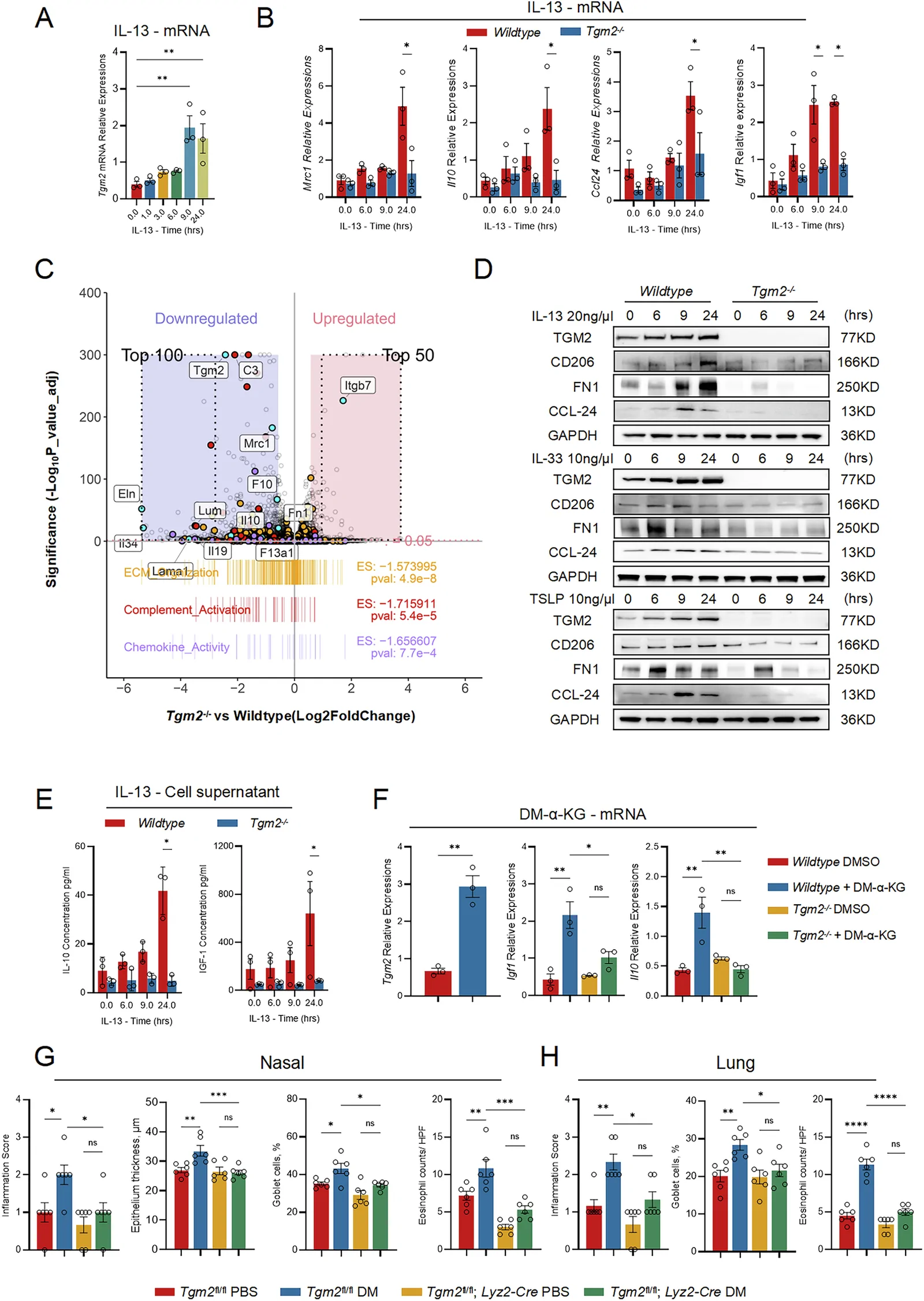
*Tgm2* is an integrative signaling hub critical for the alternative activation of macrophages. **A** qRT‒PCR confirmed the time-dependent increase in *Tgm2* mRNA levels in IL-13-stimulated wild-type macrophages (*n* = 3 biological replicates). **B** qRT‒PCR confirmed the reduced mRNA expression of *Mrc1*, *Il10*, *F13a1*, and *Ccl24* in IL-13-stimulated *Tgm2*^−/−^ macrophages compared with that in wild-type controls (*n* = 3 biological replicates). **C** Volcano plot of the RNA-seq data (|log2FC| >1; FDR < 0.05) for genes whose expression differed between IL-13-stimulated *Tgm2*^−/−^ and wild-type peritoneal macrophages, with GSEA highlighting impaired extracellular matrix organization (NES = −1.574; *p* = 4.9 × 10^−8^), complement activation (NES = −1.716; *p* = 5.4 × 10^−5^) and chemokine activity (NES = −1.657; *p* = 7.7 × 10^−4^). **D** Western blots showing that the induction of AAM markers (TGM2, CD206, FN1, and CCL-24) in *Tgm2*^−/−^ macrophages was abolished by stimulation with IL-13 (20 ng/μL), IL-33 (10 ng/μL), or TSLP (10 ng/μL) for the indicated time points within 24 hours. **E** ELISA revealed marked reductions in the levels of secreted IL-10 and IGF-1 in supernatants from IL-13-stimulated *Tgm2*^−/−^ macrophages compared with those from wild-type controls (*n* = 6 at each time point). **F** qPCR revealed reduced mRNA expression of *Il10* and *Igf1* in *Tgm2*^−/−^ macrophages compared with that in wild-type controls stimulated with DM-α-ketoglutarate (DM-α-KG, 10 μM), a membrane-permeable form of α-KG. Quantitative scoring confirmed reduced inflammation, goblet cell hyperplasia and eosinophil counts in the nasal mucosa **G** and lung tissue **H** of *Tgm2*-cKO mice. *p < 0.05; ns not significant. (by multiple unpaired *t* tests with Benjamini–Hochberg (FDR) correction)

To further elucidate the role of TGM2 in macrophage activation, we isolated primary peritoneal macrophages from wild-type and *Tgm2*^−/−^ mice and exposed them to distinct stimuli to induce different activation states. TGM2 expression was selectively induced by type 2 cues but not by classical proinflammatory stimuli, highlighting its specific responsiveness to type 2 cytokine signaling (Fig. 5A and Supplementary Fig. S8A, B).

Upon IL-13 stimulation, the mRNA expression of canonical AAM markers, including *Mrc1*, *Il10*, *Igf1*, and *Ccl24*, was significantly impaired in *Tgm2*^*−/−*^ macrophages (Fig. 5B). Bulk transcriptomic profiling confirmed broader suppression of both AAM signature genes (*Arg1*, *Retnla*, *Chil3*, *etc*.) and AAM-associated effector molecules, including ECM remodeling mediators (*Fn1*, *F13a1*, *F10*, *Lama3*, *Eln*, *etc*.) and chemokines (*Ccl11*, *Ccl24*, *Ccl28*, *etc*.) in IL-13-treated *Tgm2*-deficient macrophages (Fig. 5C and Supplementary Fig. S9A, B), which is consistent with the results of scRNA-seq and supports the role of TGM2 in AAM programming. Complement activation genes (*C2*, *C3*, *C4b*, *C1qa*, *C1qb*, etc.) together with antigen presentation-associated MHC-II molecules (*H2-Aa*, *H2-Ab1*, *H2-Eb1*) were also downregulated (Fig. 5C and Supplementary Fig. S9C), indicating widespread disruption of effector functions. Notably, the expression of the panmacrophage lineage markers *Cd68* and *Adgre1* (F4/80) was largely preserved without functionally significant alterations under standard differential criteria, whereas the expression of the M1-associated gene *Cd86* was upregulated in *Tgm2*-deficient macrophages (Supplementary Fig. S9A), further supporting the selective impairment of the alternative activation program rather than a general defect in macrophage identity. GSEA and KEGG pathway analyses further confirmed that *Tgm2* deletion predominantly downregulated ECM-related signaling pathways (Supplementary Fig. S9D–F). These transcriptional changes are faithfully translated into reduced protein levels of AAM-associated functional players. Specifically, western blot analyses confirmed a distinct decrease in the expression of the AAM effectors CD206, CCL-24, and FN1 (Fig. 5D and Supplementary Fig. S10A). Concurrently, ELISA measurements confirmed a marked decrease in the secretion of the other two AAM effectors, IL-10 and IGF-1 (Fig. 5E). Together, these dual-layered validations provide conclusive evidence that TGM2 is essential for establishing the characteristic AAM phenotype.

In addition to IL-13, we investigated whether TGM2 expression could be induced by other factors known to promote alternative activation of macrophages, including IL-33, thymic stromal lymphopoietin (TSLP), dimethyl α-ketoglutarate (DM-α-KG) and itaconate. Stimulation of wild-type macrophages with IL-33, TSLP, or DM-α-KG, but not itaconate, resulted in a significant upregulation of TGM2 expression, similar to the effects observed with IL-13 (Supplementary Fig. S8A, B). Notably, the absence of *Tgm2* inhibited the upregulation of AAM-associated gene expression induced by these factors, further underscoring the central role of TGM2 in the differential activation of macrophages (Fig. 5D, F and Supplementary Fig. S10B, C). These findings suggest that TGM2 serves as a critical integrator of diverse signals, enabling macrophages to adopt an AAM phenotype in response to various microenvironmental cues.

To test whether the contribution of TGM2 to dust mite-induced T2I is dependent on its function in alternative macrophage activation, we generated *Tgm2* flox mice (*Tgm2*^fl/fl^) and crossed them with *Lyz2*-Cre mice to achieve macrophage-specific deletion of *Tgm2*. Using immunofluorescence staining, we confirmed the selective deletion of *Tgm2* in macrophages (Supplementary Fig. S10D). Compared with *Tgm2*^fl/fl^ control mice, *Tgm2*^fl/fl^; *Lyz2*-Cre mice exhibited a significant alleviation of the pathological airway phenotype, although to a lesser extent, which was characterized by a robust reduction in nasal epithelial thickening, lower inflammation scores, and significantly lower numbers of goblet cells and eosinophil counts in both nasal and lung tissues (Fig. 5G, H and Supplementary Fig. S10E–I). Together, these findings establish TGM2 as a key regulator of differential AAM activation and demonstrate that macrophage-specific TGM2 expression is indispensable for driving T2I and ECM remodeling in airway disease, suggesting that the role of nonmacrophage TGM2 reservoirs in facilitating full-scale structural remodeling is complementary.

### TGM2-mediated H3Q5Ser promotes AAM programming

Although *Tgm2* deficiency caused widespread transcriptomic alterations, protein array analysis revealed no significant changes in canonical signaling cascades upstream of macrophage activation (Supplementary Fig. S11A). This uncoupling between transcriptional rewiring and upstream signaling prompted the hypothesis that TGM2 regulates gene expression through a more direct mechanism, potentially at the transcriptional or epigenetic level.

**Fig. 6:**
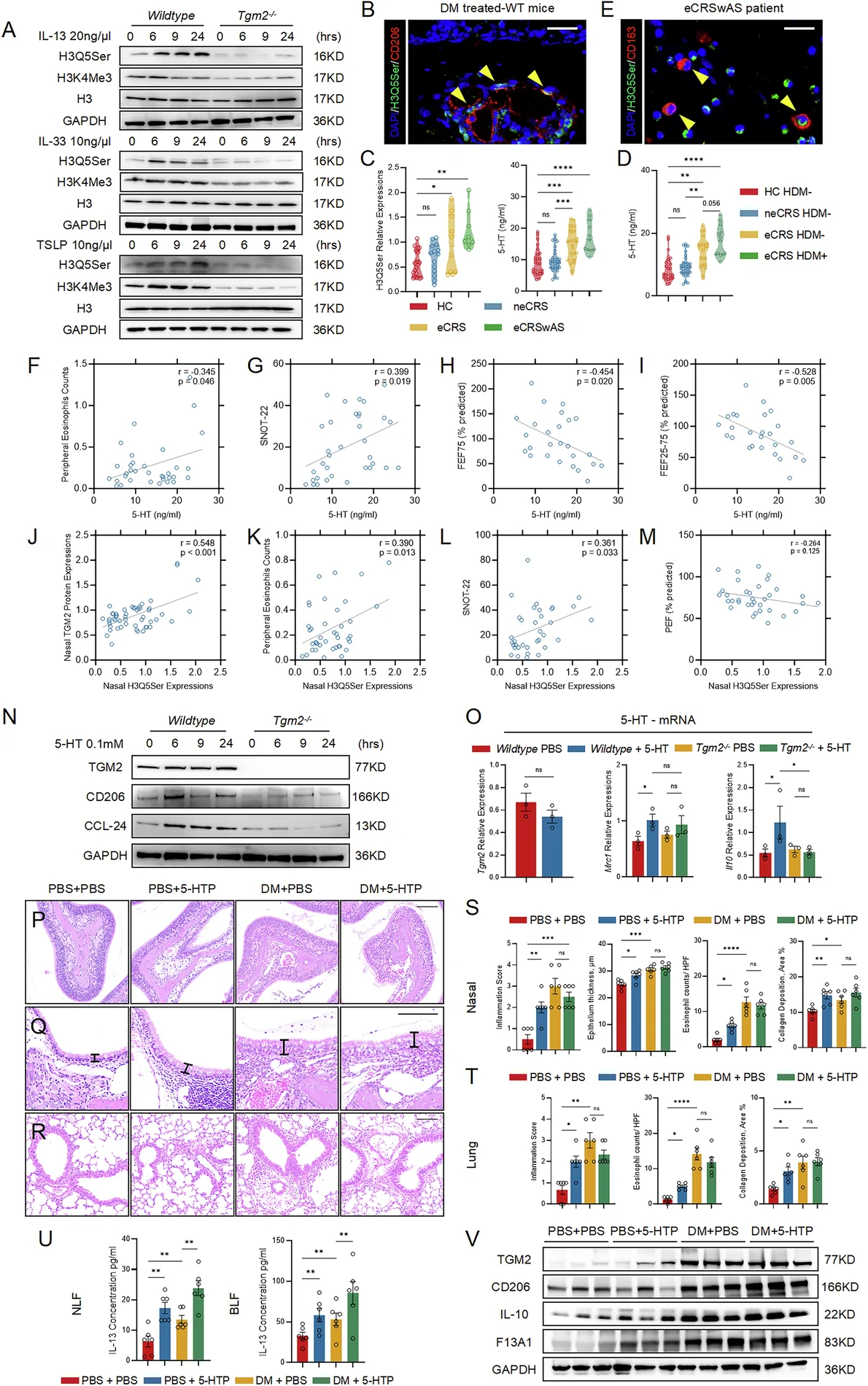
TGM2-mediated H3Q5Ser promotes alternative activation of macrophages. **A** Western blotting of H3Q5Ser and H3K4Me3 levels in wild-type and *Tgm2*^−/−^ macrophages upon stimulation with IL-13 (20 ng/μL), IL-33 (10 ng/μL), or TSLP (10 ng/μL) for the indicated time points within 24 hours. **B** Representative multicolor immunofluorescence images showing H3Q5Ser (green) colocalization with CD206^+^ macrophages (red) in the nasal mucosa of dust mite-treated wild-type mice. Nuclei were counterstained with DAPI (blue). Scale bar: 100 μm. Corresponding images from untreated wild-type and *Tgm2*^−/−^ mice are presented in Supplementary Fig. S12C. **C** Quantitative analysis revealed significantly higher H3Q5Ser (HC = 20, neCRS=14, eCRS=10, eCRSwAS=8) and 5-HT levels (ELISA measurement) (HC = 24, neCRS=28, eCRS=23, eCRSwAS=11) in the eCRS and eCRSwAS groups than in the other groups. **D** ELISA revealed that compared with nonsensitized patients (*n* = 20), dust mite-sensitized eCRS patients (*n* = 13) had suggestive elevated 5-HT levels (*p* = 0.056). **E** Representative multicolor immunofluorescence images showing H3Q5Ser (green) colocalization with CD136^+^ macrophages (red) in nasal polyp tissues from eCRSwAS patients. The corresponding images of the tissues from the healthy controls and the neCRS and eCRS patients are presented in Supplementary Fig. S12F. 5-HT levels in nasal mucosa samples were positively correlated with peripheral eosinophil counts (**F**, *n* = 34) and SNOT-22 scores (**G**, *n* = 34) but negatively correlated with lung function parameters, including FEF75% (**H**, *n* = 26) and FEF25-75% (**I**, *n* = 26). The nasal H3Q5Ser expression level was positively correlated with the TGM2 expression level (**J**, *n* = 52), peripheral eosinophil count (**K**, *n* = 40) and SNOT-22 score **(L**, *n* = 35) but was not correlated with the FEV1 level (**M**, *n* = 35). **N** Western blot analysis of CD206, TGM2 and CCL-24 expression in wild-type and *Tgm2*^−/−^ macrophages stimulated with 5-HT (0.1 mM) at the indicated time points within 24 hours (by multiple unpaired *t* tests with Benjamini–Hochberg (FDR) correction). **O** qRT‒PCR results showing the time-dependent induction of *Mrc1*, *Tgm2* and *Il10* mRNA expression in 5-HT-treated wild-type versus *Tgm2*^−/−^ macrophages. Representative HE staining revealed inflammation and remodeling in nasal mucosa **P**, **Q** and lung (**R**) tissues from mice cotreated with 5-HTP and dust mites, as well as in the groups treated with 5-HTP and dust mites alone. Scale bar: 100 μm. Quantitative histopathology scoring confirmed increased inflammation, eosinophil infiltration and collagen deposition in the nasal mucosa **S** and lung **T** tissues of the 5-HTP+dust mite groups compared with those of the dust mite alone group. **U** ELISA quantification revealed synergistic increases in the levels of IL-13 in NLF and BLF from mice coexposed to dust mites and 5-HTP (10 mg/kg i.p.) versus those from mice treated with dust mites alone (*n* = 6/group). **V** Western blot analysis revealed increased expression of AAM markers (CD206, TGM2, F13A1, and IL-10) in the nasal mucosa of mice cotreated with 5-HTP and dust mites. The data are presented as the mean ± SEM. *p < 0.05, **p < 0.01, ***p < 0.001, ****p < 0.0001; ns not significant (two-tailed unpaired *t*-test and one-way ANOVA with Dunnett’s test for difference analysis, Spearman’s correlation analysis for correlation analysis)

We therefore focused on the specific epigenetic modification of histone H3 glutamine-5 serotonylation (H3Q5Ser), as TGM2 is its known catalytic enzyme and facilitates TFIID recruitment to increase transcription [30]. To determine whether TGM2 affects H3Q5Ser in macrophages, we stimulated wild-type and *Tgm2*^−/−^ macrophages with IL-13, IL-33, TSLP, or DM-α-KG. While these type 2 stimuli robustly triggered H3Q5Ser in wild-type macrophages, with IL-13 also inducing a significant increase in H3K4Me3 levels, the induction of both epigenetic markers was profoundly abrogated in the absence of *Tgm2* (Fig. 6A and Supplementary Fig. S12A, B). In vivo, immunofluorescence staining of nasal tissues from dust mite-exposed mice revealed strong H3Q5Ser signals specifically within macrophages in wild-type but not *Tgm2*^*−/−*^ mice (Fig. 6B and Supplementary Fig. S12C, D), suggesting that this modification is linked to TGM2-mediated AAM programming.

We next validated the clinical relevance of this axis in human patients. In line with the murine data, nasal tissues from patients with eCRS and eCRSwAS presented significantly higher levels of 5-HT and H3Q5Ser than those from healthy or neCRS individuals did (Fig. 6C, D and Supplementary Fig. S12E). Immunofluorescence staining demonstrated pronounced enrichment of H3Q5Ser specifically in CD163^+^ macrophages within these lesions (Fig. 6E and Supplementary Fig. S12F, G). We then assessed the clinical associations linked to the abundance of 5-HT and H3Q5Ser. The mucosal levels of 5-HT were positively correlated with peripheral eosinophil counts and symptom severity (SNOT-22) but negatively correlated with lung function (FEF75% and FEF25-75%) (Fig. 6F–I). H3Q5Ser levels were strongly positively correlated with TGM2 expression and key disease severity markers, including eosinophil counts and SNOT-22 scores (Fig. 6J–M). These data firmly establish H3Q5Ser as a clinically relevant epigenetic marker in human type 2 airway inflammation.

We then investigated whether 5-HT is sufficient to activate this pathway and drive pathology both in vitro and in vivo. In the in vitro assay, stimulation of wild-type macrophages with 5-HT robustly increased H3Q5Ser expression and AAM gene expression, and these effects were abrogated in *Tgm2*^*−/−*^ macrophages (Fig. 6N, O and Supplementary Fig. S12H). For the in vivo assessment, we administered 5-hydroxytryptophan (5-HTP, a precursor of 5-HT) to naïve or dust mite-challenged mice intraperitoneally. In naϊve mice, 5-HTP alone was sufficient to recapitulate key features of T2I, including eosinophilic infiltration, elevated IL-13 and 5-HT levels, and tissue remodeling in both the nasal and pulmonary compartments (Fig. 6P–U and Supplementary Fig. S13A–E). Notably, in dust mite-sensitized mice, 5-HTP increased the expression of IL-13 but did not further exacerbate other pathologies (Supplementary Fig. S13F), suggesting that the effects of 5-HTP and allergen exposure converge on shared downstream pathways. Molecular analyses confirmed that 5-HT directly upregulated the expression of classical T2I effector molecules, including F13A1 and IL-10, further substantiating its proinflammatory role (Fig. 6V and Supplementary Fig. S13G–I). In summary, our multilevel data establish the 5-HT-TGM2-H3Q5Ser axis as a fundamental epigenetic mechanism driving alternative macrophage activation and T2I.

### Platelet-derived 5-HT fuels macrophage H3Q5 serotonylation to drive type 2 inflammation

**Fig. 7:**
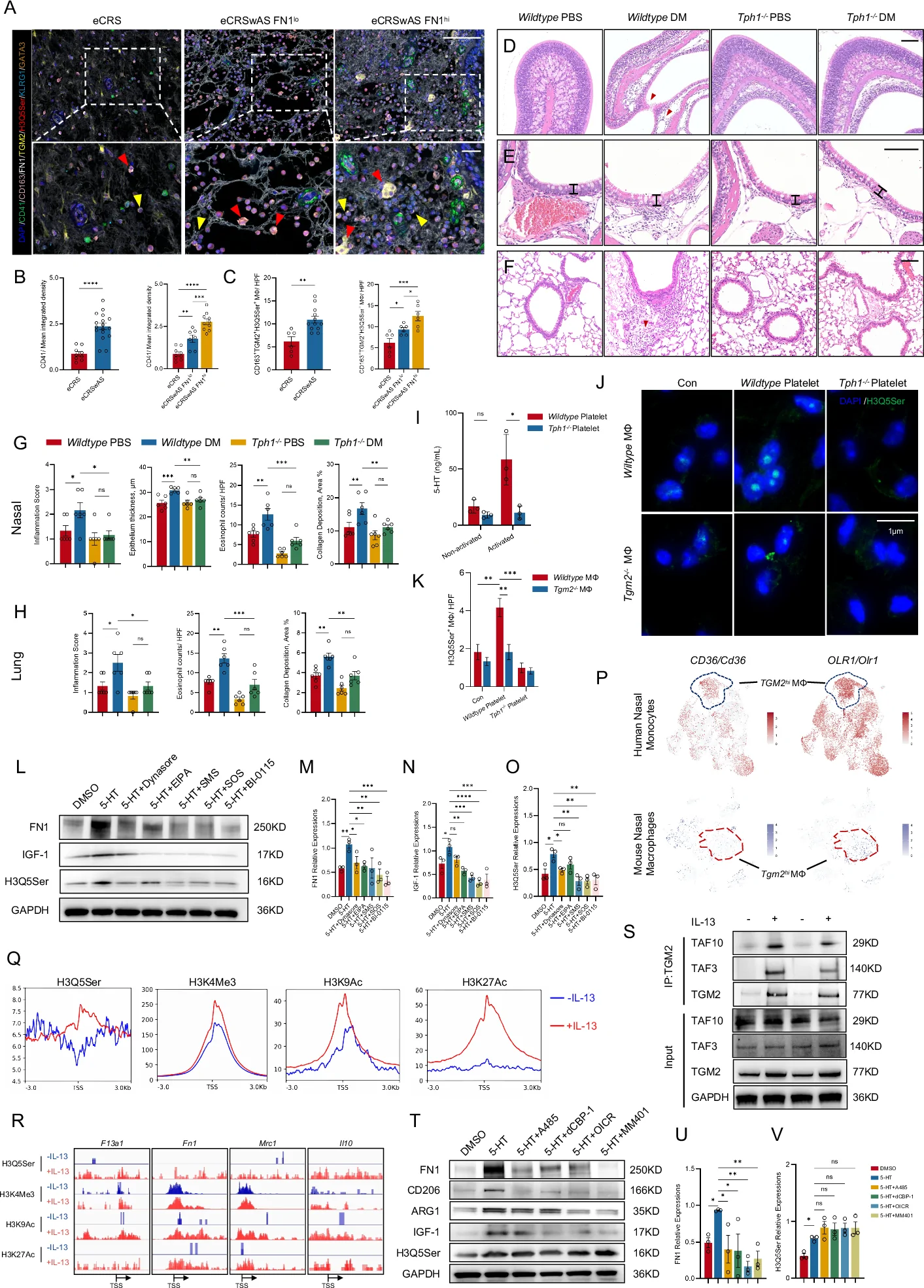
Platelet-derived 5-HT fuels H3Q5 serotonylation to establish a chromatin landscape driving type 2 inflammation. **A** Multiplex immunohistochemistry revealed an increased density of CD41⁺ platelets (green) in FN1^high^ (white) stromal regions with prominent vascular structures. Notably, CD163⁺ (pink), TGM2⁺ (yellow), H3Q5Ser⁺ (red) macrophages (indicated by the red arrow) and GATA3^+^ (orange), KLRG1^+^ (cyan) cells (indicated by the yellow arrow) were frequently located adjacent to these platelet-enriched zones. Scale bar: 100 µm. Quantitative analysis revealed the expansion of platelets **B** and CD163^+^ TGM2^+^ H3Q5Ser^+^ macrophages **C** in nasal polyp tissues from eCRSwAS patients compared with those from eCRS controls, especially in FN1-abundant areas. Representative HE staining images revealing inflammation and remodeling in nasal mucosa **D**, **E** and lung **F** tissues from dust mite-challenged wild-type and *Tph1*^−/−^ mice. Scale bar: 100 µm. Quantitative comparison of inflammation scores, epithelial thickness, eosinophil infiltration and collagen deposition in nasal mucosa **G** and lung tissues **H** between dust mite-treated wild-type and *Tph1*^−/−^ mice. **I** ELISA detection of 5-HT in the supernatant of platelets isolated from wild-type and *Tph1*^−/−^ mice with or without low-dose thrombin activation. **J**, **K** Immunofluorescence staining revealed that thrombin-activated platelets isolated from wild-type mice, but not *Tph1*^−/−^ mice, induced H3Q5Ser modification in wild-type macrophages, whereas *Tgm2*^−/−^ macrophages remained unresponsive. Scale bar: 2 µm. **L**–**O** Western blot analysis revealed that the addition of dynasore (80 μM), EIPA (50 μM), SMS121 (CD36 inhibitor, 20 μM), SOS (CD36 inhibitor, 20 μM) and BI-0115 (OLR1/LOX-1 inhibitor, 5 μM) altered the levels of FN1 **M**, IGF-1 **N** and H3Q5Ser **O** expression in macrophages induced by 5-HT. **P** Feature plots showing the expression patterns of *CD36/Cd36* and *OLR1/Olr1* across monocytes from human nasal polyps (top) or macrophages from dust mite-challenged mouse nasal mucosa (bottom), with dashed circles highlighting the most prominent *TGM2*^hi^ or *Tgm2*^hi^ macrophage population. **Q** Metagene profiles revealed significant enrichment of H3Q5Ser, H3K4Me3, H3K9Ac, and H3K27Ac at transcriptional start sites (TSSs) after IL-13 stimulation. **R** Genome browser tracks of H3Q5Ser, H3K4Me3, H3K9Ac and H3K27Ac binding at the *F13a1*, *Fn1*, *Mrc1*, and *Il10* gene loci in the control and IL-13-treated groups. **S** Coimmunoprecipitation assays demonstrating enhanced interactions between TGM2 and TAF10 or TAF3 in IL-13-treated primary macrophages from wild-type mice. GAPDH served as the loading control. **T**–**V** Western blot analysis of H3Q5Ser and the indicated AAM effector proteins in macrophages treated with 5-HT in the presence or absence of epigenetic inhibitors. Cells were cotreated with 5-HT and the H3K4 methyltransferase complex inhibitor OICR-9429 (OICR, 1 μM) or MM-401 (1 μM) or the CBP/EP300 inhibitor A485 (1 μM) or dCBP-1 (1 μM). Data: mean ± SEM; *p < 0.05, **p < 0.01, ***p < 0.001 (one-way ANOVA with Dunnett’s test)

Peripheral serotonin (5-HT) is synthesized primarily by TPH1 in gut enterochromaffin cells and is stored in circulating platelets [36]. We therefore hypothesized that this platelet-derived 5-HT pool could be mobilized to fuel inflammatory processes. To address this hypothesis, we first examined platelet distribution in nasal polyp tissues from eCRS and eCRSwAS patients. mIHC results revealed that, compared with eCRS samples, eCRSwAS tissues exhibited a significant enrichment of CD41⁺ platelets, particularly within FN1-high (FN1^hi^) stromal regions surrounding prominent vascular structures (Fig. 7A, B). These platelets frequently formed aggregates in perivascular zones, suggesting localized activation or recruitment. Notably, the densities of CD163⁺ TGM2⁺ H3Q5Ser⁺ macrophages and GATA3^+^ KLRG1^+^ cells, which were often spatially clustered adjacent to CD41⁺ platelet aggregates, were elevated in the FN1hi regions of eCRSwAS (Fig. 7A–C and Supplementary Fig. S14A). While CD41 primarily indicates the presence of platelets rather than their activation status, this consistent colocalization implies potential crosstalk; intravascular platelets may modulate the perivascular microenvironment through paracrine signals, including 5-HT, to neighboring macrophages.

We then challenged *Tph1*^*−/−*^ mice, which lack peripheral 5-HT production, with dust mites. Compared with wild-type control mice, *Tph1*^*−/−*^ mice exhibited decreased 5-HT levels, less nasal pathology and attenuated inflammation scores, eosinophil infiltration and collagen deposition in nasal and lung tissues following dust mite exposure (Fig. 7D–H and Supplementary Fig. S14B–D), suggesting that peripheral 5-HT contributes critically to the development of type 2 airway inflammation. Consistently, ELISA confirmed a drastic reduction in 5-HT in the supernatant of low-dose thrombin-activated platelets isolated from *Tph1*^*−/−*^ mice (Fig. 7I). Using a coculture system, we demonstrated that these activated wild-type platelets, but not *Tph1*^*−/−*^ platelets, significantly induced H3Q5Ser modification in wild-type macrophages, while *Tgm2*^−/−^ macrophages remained unresponsive (Fig. 7J, K).

We therefore sought to determine the mechanism by which macrophages internalize 5-HT. Interrogation of our scRNA-seq data revealed that macrophages in both human and mouse nasal mucosa largely lacked expression of canonical 5-HT transporters or receptors (Supplementary Fig. S15A, B), ruling out the classical uptake pathway. Instead, we found that dynasore, a potent inhibitor of dynamin-dependent endocytosis, markedly suppressed the increase in H3Q5Ser, FN1, and IGF-1 induced by 5-HT (Fig. 7L–O). This finding revealed that 5-HT entry requires a dynamin-dependent endocytic process. Since scavenger receptors are master regulators that can initiate the endocytosis of diverse soluble molecules, we focused on CD36 and OLR1. Both were highly expressed in *TGM2*^*hi*^ macrophages in human and mouse nasal tissues (Fig. 7P). Molecular docking further predicted stable interactions between 5-HT and CD36 or OLR1, primarily driven by strong van der Waals forces (Supplementary Fig. S15C, D). Functionally, pharmacological blockade of CD36 or OLR1 significantly inhibited 5-HT-induced H3Q5Ser and the expression of alternative activation markers (Fig. 7L–O). Thus, CD36 and OLR1 serve as critical scavenger receptors that mediate the endocytic uptake of 5-HT, enabling its subsequent role in epigenetic reprogramming.

### H3Q5Ser collaborates with active chromatin marks and the TFIID complex to drive AAM gene expression

To explore the epigenetic basis of H3Q5Ser-mediated AAM programming, we performed CUT&Tag profiling on IL-13-stimulated macrophages. Genome-wide analysis revealed that IL-13 stimulation induced substantial enrichment of H3Q5Ser, H3K9Ac, and H3K27Ac around transcriptional start sites, a pattern observed both across the genome and with pronounced enrichment of canonical AAM marker genes (Fig. 7Q and Supplementary Fig. S16A–F). Although the overall enrichment of H3K4Me3 remained stable in contrast to these global increases, differential peak analysis revealed that IL-13 selectively increased H3K4Me3 signaling at key AAM-associated loci, such as *Mrc1* and *Arg1* (Supplementary Fig. S16B), suggesting a targeted increase in promoter permissiveness at these genes. Notably, H3Q5Ser peaks were strongly colocalized with active chromatin markers (H3K4Me3, H3K9Ac, and H3K27Ac), as well as TGM2, at the promoter regions of AAM genes (*F13a1*, *Fn1*, *Mrc1*, and *Il10*) (Fig. 7R and Supplementary Fig. S16G, H), suggesting that H3Q5Ser facilitates the transcriptional initiation and activation of AAM-associated genes through the establishment of a permissive chromatin state.

To determine the impact of *Tgm2* deficiency on the epigenetic landscape, we performed ATAC-seq and found no significant alterations in global chromatin accessibility (Supplementary Fig. 16I). However, quantitative PCR analysis of CUT&Tag-enriched DNA (CUT&Tag-qPCR) focusing on *Mrc1*, a representative AAM gene with prominent H3Q5Ser enrichment in our CUT&Tag data, revealed that *Tgm2*^−/−^ macrophages had markedly reduced levels of H3Q5Ser, H3K4Me3, H3K9Ac, and H3K27Ac at its promoter (Supplementary Fig. 16J), which is consistent with the downregulation of *Mrc1* mRNA expression upon *Tgm2* deficiency. These results demonstrate that TGM2 does not broadly remodel chromatin architecture but instead regulates local histone modifications, particularly H3Q5 serotonylation, to establish transcriptionally permissive loci.

Given this locus-specific regulation, we next examined whether TGM2-mediated H3Q5Ser facilitates AAM effector transcription via the H3K4me3-TFIID axis. Single-cell RNA-seq datasets revealed the coexpression of *TGM2* with the TFIID subunit *TAF10*, but not *TAF3*, in macrophages from both human eCRS nasal polyps and dust mite-exposed mouse nasal mucosa (Supplementary Fig. S16K). Consistently, coimmunoprecipitation demonstrated enhanced interactions between TGM2 and TAF10 or TAF3 in IL-13-treated macrophages (Fig. 7S). Most importantly, pharmacological inhibition of the H3K4 methyltransferase complex (using OICR-9429 or MM-401) or the acetyltransferases CBP/EP300 (using A485 or dCBP-1) markedly suppressed 5-HT-induced, H3Q5Ser-dependent alternative macrophage activation, with little effect on H3Q5Ser itself (Fig. 7T–V and Supplementary Fig. S16L). Collectively, these findings define a model in which H3Q5ser cooperates with the H3K4Me3-TFIID axis to establish an epigenetic framework essential for alternative macrophage activation.

### Pharmacologic inhibition of the 5-HT-TGM2-H3Q5Ser axis attenuates type 2 airway inflammation

**Fig. 8:**
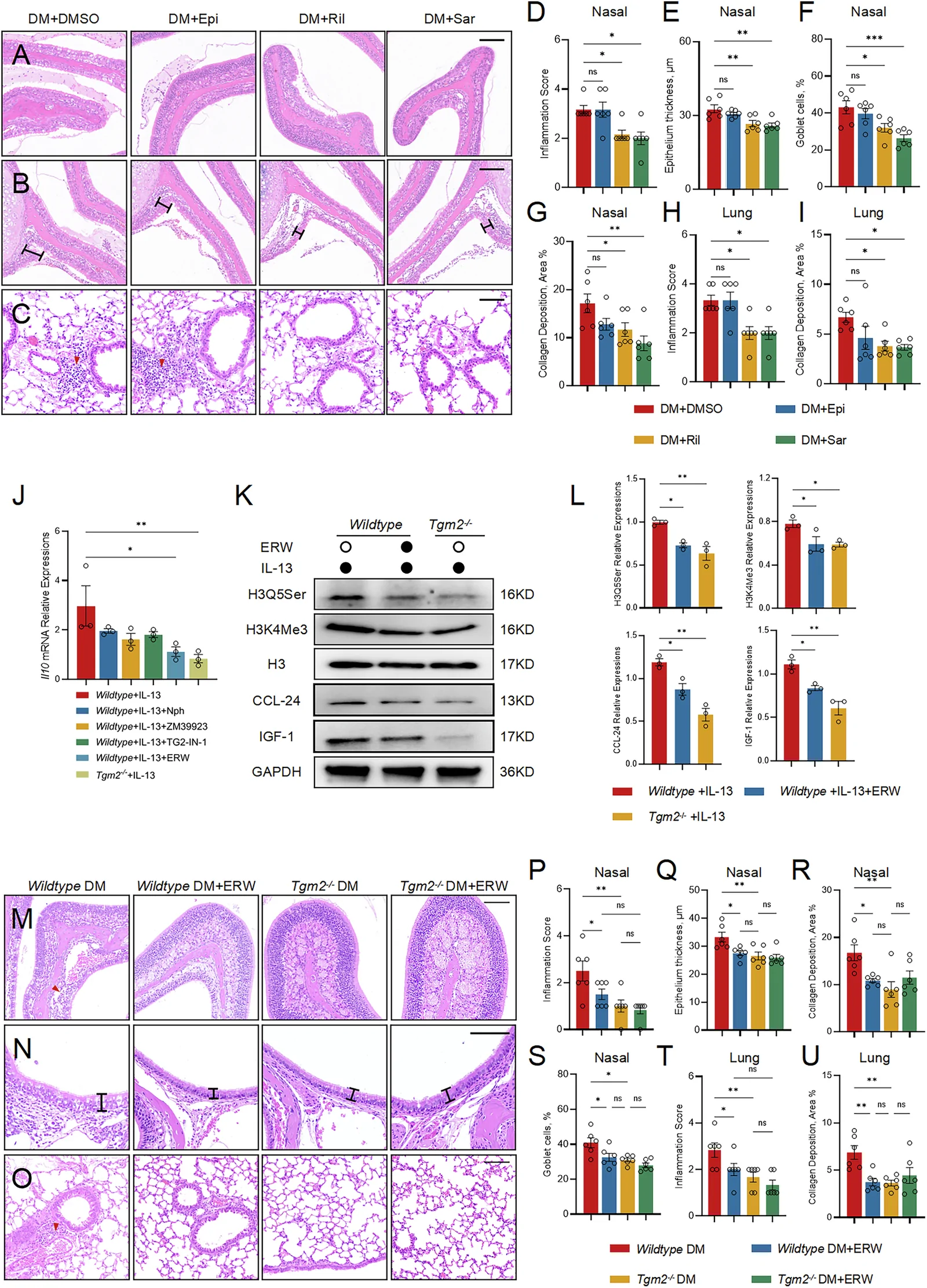
Pharmacological inhibition of the 5-HT–TGM2–H3Q5Ser axis attenuates type 2 airway inflammation. **A**–**C** Representative HE staining revealed inflammation and remodeling in nasal mucosa **A**, **B** and lung C tissues, indicating a comparative evaluation of the therapeutic efficacy of (-)-epicatechin (Epi, 1 mg/kg i.n.), sarpogrelate (Sar, 5 mg/kg i.n.) and rilapladib (Ril, 0.2 mg/kg i.n.) in dust mite-challenged wild-type mice. Scale bar: 100 µm. Quantitative comparison of inflammation scores **D**, epithelial thickness **E**, goblet cell hyperplasia **F** and collagen deposition **G** in the nasal mucosa and inflammation scores **H** and collagen deposition **I** in lung tissues from dust mite-challenged wild-type mice treated with the indicated compounds. **J** qRT‒PCR analysis of the effects of TGM2 inhibitors (10 μM NPT, 10 μM TG2-IN-1, 10 μM ZM39923, and 10 μM ERW1041E) on *Il10* expression in IL-13-treated macrophages, with ERW1041E showing maximal inhibition (*p* < 0.01). **K**–**L** Western blot analysis demonstrated that ERW1041E (10 μM) recapitulates *Tgm2* deletion by reducing H3Q5Ser and H3K4Me3 levels, as well as CCL-24 and IGF-1 expression levels, in IL-13-stimulated macrophages. Representative HE staining revealed inflammation and remodeling in nasal mucosa **M, N** and lung **O** tissues, indicating the therapeutic efficacy of ERW1041E (10 mg/kg/day i.n.) in dust mite-challenged wild-type and *Tgm2*^−/−^ mice. Quantitative comparison of inflammation scores **P**, epithelial thickness **Q**, goblet cell hyperplasia **R** and collagen deposition **S** in the nasal mucosa and inflammation scores **T** and collagen deposition **U** in lung tissues. Scale bar: 100 µm. Data: mean ± SEM; *p < 0.05, **p < 0.01, ***p < 0.001 (one-way ANOVA with Dunnett’s test)

We next asked whether the 5-HT-TGM2-H3Q5Ser axis is pharmacologically tractable in vivo. To test this hypothesis, we treated dust mite-challenged mice with compounds that modulate platelet-derived 5-HT release intranasally. Both sarpogrelate hydrochloride (Sar), a selective 5-HT2A receptor antagonist that suppresses 5-HT release [37], and rilapladib (Ril), a platelet-activating factor receptor (PAFR) inhibitor that blocks platelet activation [38], significantly reduced dust mite-induced eosinophil infiltration and H3Q5Ser levels (Fig. 8A-I and Supplementary Fig. S17A-D). In contrast, (-)-epicatechin (Epi), a COX-1 inhibitor targeting platelet aggregation [39], exhibited minimal efficacy. These results suggest that targeting upstream components of the platelet–5-HT pathway can effectively blunt H3Q5 serotonylation and downstream inflammatory responses in mice.

To investigate the therapeutic potential of targeting TGM2 in type 2 airway inflammation, we first screened four specific TGM2 inhibitors (NPh, TG2-IN-1, ZM39923, and ERW1041E) [40] in IL-13-stimulated macrophages. All the compounds significantly suppressed IL-10 production, with ERW1041E emerging as the most potent (Fig. 8J). ERW1041E treatment significantly reduced H3Q5Ser and H3K4Me3 levels, accompanied by decreased CCL-24 and IGF-1 protein expression induced by IL-13 (Fig. 8K, L). Intranasal administration of ERW1041E also significantly ameliorated dust mite-induced airway inflammation in wild-type mice but had no additional effect on *Tgm2*^−/−^ mice, confirming its TGM2-dependent mechanism of action (Fig. 8M–U and Supplementary Fig. 17E, F). In summary, these findings establish the 5-HT-TGM2-H3Q5Ser axis as a pharmacologically tractable pathway in which targeting either platelet-derived serotonin release or TGM2 enzymatic activity effectively attenuates T2I.

## Discussion

Our study reveals a transcellular circuit in which platelet-derived 5-HT epigenetically reprograms macrophages to drive T2I. While AAMs are integral to the pathogenesis of eCRS and asthma, the systemic signals that instruct their proinflammatory functions remain poorly defined [12]. Here, we identify activated platelets as key upstream regulators of AAMs. We demonstrate that 5-HT, which is stored and released by platelets, is internalized by macrophages and fuels the epigenetic machinery for alternative activation. This process is orchestrated by TGM2, which we established as a key marker of profibrotic AAMs. Mechanistically, platelet-derived 5-HT serves as the essential substrate for TGM2-mediated H3Q5ser, thereby directly coupling vascular activation to intracellular epigenetic rewiring in macrophages. This platelet-5-HT-TGM2 signaling cascade, which is spatially organized within perivascular niches, functions as a crucial epigenetic checkpoint for activating AAM-associated gene programs. Our work thus defines a central axis for AAM-driven pathology that is under the control of platelet activation, highlighting the therapeutic potential of targeting this interface between coagulation and inflammation.

Our findings suggest that serotonin (5-HT) acts not only as a neurotransmitter but also as a systemic immunometabolic messenger, translating upstream signals from the gut microbiome and immune system into downstream airway pathology. The levels of the circulating metabolite of the gut are regulated by intestinal homeostasis [41–43]. For example, *Turicibacter*, a bacterial genus linked to eosinophilic infiltration in CRSwNP, can modulate 5-HT production [44]. In this pathway, platelets function as systemic distributors, delivering 5-HT to inflammatory sites where it drives the epigenetic reprogramming of macrophages. Our work thus defines a gut-platelet-macrophage axis, revealing how upstream metabolic disturbances in the gut can shape T2I. Therefore, targeting the production or activity of this gut-derived messenger may represent a novel therapeutic strategy.

Importantly, the regulatory mechanism defined herein is complementary to previously established platelet- and 5-HT-dependent immune signaling pathways. Previous work has demonstrated that platelets expressing CD154 can skew type 2 immune responses by interacting with CD40 on dendritic cells (DCs) or eosinophils [45–48]. In parallel, 5-HT has been functionally implicated in enhancing the DC-driven differentiation of T helper 17 (Th17) cells [49]. Notably, however, the administration of 5-hydroxytryptophan (5-HTP), the upstream precursor of 5-HT, has yielded pleiotropic biological outcomes. For instance, independent studies have reported that exogenous 5-HTP administration has inhibitory effects on allergic inflammation [50, 51]. Conversely, other investigations have shown that 5-HTP can promote IL-13-driven intestinal inflammation [52]. Moreover, 5-HTP has a well-documented risk of promoting eosinophil expansion and infiltration in humans [53]. These multifaceted, context-dependent effects may stem from its high sensitivity to experimental variables, including dosing regimens, purity, potential contaminants, and animal models. Unlike prior pathways, our integrated array of multidimensional data systematically demonstrated that the endogenous platelet-5-HT-macrophage axis functions as an indispensable positive driver of TGM2-dependent alternative macrophage activation, confirming that the primary in vivo net effect of this signaling pathway is pathogenic rather than protective in dust mite-induced type 2 mucosal immunity.

Notably, this macrophage 5-HT uptake does not rely on classical membrane receptors or transporter pathways but instead occurs through CD36/OLR1-mediated endocytosis to deliver 5-HT into the intracellular compartment for H3Q5Ser modification. In type 2 immune responses, CD36 is a well-established marker of alternative macrophage activation [54, 55], and its role in promoting airway inflammation aligns consistently with the functional direction of the 5-HT-TGM2-H3Q5Ser axis in our model [56]. Furthermore, the capacity of CD36 and OLR1 to mediate receptor-mediated endocytosis [57, 58] supports the plausibility of this cellular entry route. These insights suggest that 5-HT utilizes distinct but complementary pathways across time and space, while classical membrane receptors sense rapid microenvironmental changes to trigger acute responses [59], the internalization route via CD36/OLR1-mediated endocytosis allows 5-HT to drive persistent, long-term epigenetic remodeling via TGM2, together regulating the full spectrum of macrophage activation from immediate adaptation to sustained tissue pathology.

Our findings reveal that TGM2 promotes type 2 airway inflammation and fibrosis progression through a coordinated, synergistic multicellular mechanism across both myeloid and structural compartments. Previous research has underscored the multifunctionality of TGM2, with extensive evidence supporting its pivotal role in fibrosis during chronic inflammatory disease, primarily through its ability to maintain ECM integrity through transglutaminase activity and interactions with TGF-β-SMAD signaling to promote fibroblast activation [60–62]. Our study reveals an evolutionarily conserved role of TGM2 as a master regulator of pro-fibrotic macrophage fate with high expression of *FN1*. This population, which exists on a continuous trajectory of *TGM2*^hi^ and *FN1*^hi^ differentiation in humans and as a coexpressing population in mice, is a major contributor to fibrosis across organs (e.g., pulmonary, kidney or hepatic fibrosis) [63–65]. In our study of type 2 inflammation, we not only confirmed that FN1 originates predominantly from macrophages but also identified TGM2 as the molecular linchpin that drives this program epigenetically through H3Q5 serotonylation. The conservation of this TGM2-FN1 axis from murine models to human eCRS suggests its broader relevance in fibrotic diseases [66].

Furthermore, multidimensional changes in macrophage functions represent key aspects of macrophage cellular adaptation to different pathophysiological environments [67]. Our results revealed that *TGM2*^+^ macrophages represent a specialized AAM subset; within this population, TGM2 is not merely a passive marker associated with AAM polarization but also acts as a specific core regulator of this program by controlling the expression of key AAM genes, including those profibrotic factors. Indeed, transcriptomic analysis revealed that the role of TGM2 extends beyond alternative activation, as ECM production, the complement cascade and MHC-II expression are also significantly impaired upon *TGM2* deficiency. Although classical M1-associated signaling remained largely unchanged or showed a minor compensatory increase, our findings are derived from a dust mite-induced model, which represents a typical type 2 airway inflammation microenvironment. Therefore, the exact role and underlying mechanisms of TGM2 in classical M1-dominant inflammatory conditions remain unclear. Owing to differences in tissue baselines and microenvironments, further independent studies using nontype 2 inflammation models are needed to fully clarify its broader functions.

Importantly, the profibrotic function of TGM2 is not confined to macrophages. The milder protective effect observed with myeloid-specific *Tgm2* deletion than with global *Tgm2* knockout aligns with our detection of high *TGM2* expression in airway structural cells, including fibroblasts and epithelial and endothelial cells. We thus infer that *TGM2* in these nonmyeloid compartments contributes to disease pathogenesis. These complementary, cell-specific *TGM2* functions collectively drive type 2 airway inflammatory disease, with their relative contributions varying dynamically across disease stages and pathological contexts. Further dissection of cell type-specific *TGM2* functions will therefore enable the development of more precise therapeutic strategies for eosinophilic airway disorders.

Recent research has highlighted the significance of TGM2-mediated protein modifications, particularly the monoamination of H3Q5, including serotonylation, dopaminylation, and histaminylation, as novel epigenetic regulatory mechanisms [68]. Among these modifications, H3Q5Ser has been extensively studied because of its role in facilitating the interaction between TFII and H3K4Me3, a process implicated in the accessibility of transcription initiation sites [30]. This modification has demonstrated functional significance across various disease contexts, including cancer, neurocognitive disorders, and metabolic diseases [31–34]. In this study, we observed a significant increase in TGM2-dependent H3Q5Ser levels in AAMs in eCRS, with pronounced enrichment at the promoter regions of AAM-associated genes. These findings define a pathogenic role for H3Q5Ser in AAM-mediated processes, particularly in T2I and fibrosis. Notably, among other monoamine modifications, both dopamine and histamine have been strongly associated with T2I development, suggesting that monoamine modification is a broad signaling mechanism. However, the potential role of H3Q5Ser and these modifications in the coregulation of T2I initiation remains to be elucidated.

In recent years, advances in the molecular biology of T2I have led to the development of biotherapeutic drugs targeting core T2I molecules, such as omalizumab (anti-IgE), dupilumab (anti-IL-4Rα), mepolizumab (anti-IL-5), and tezepelumab (anti-TSLP) [69–72]. These have been applied in T2I-related diseases, including asthma, allergic rhinitis, atopic dermatitis, and CRSwNP. However, their high cost and limited efficacy against complications such as fibrosis underscore the urgent need for novel targets and cost-effective therapies that confer both anti-inflammatory and antifibrotic effects. Previous studies have demonstrated that targeted inhibition of TGM2 is highly promising for treating cancer, fibrosis, and neurodegenerative diseases [73]. Most of these studies, however, focus on the mechanism through which TGM2 affects protein cross-linking. Our research revealed that targeted inhibition of TGM2 can alleviate T2I symptoms through the H3Q5Ser-profibrotic AAM axis, further expanding the potential applications of TGM2-targeted therapy. Additionally, inhibition of 5-HT release from platelets similarly alleviated the T2I symptoms induced by dust mites. Notably, the efficacy of inhibiting 5-HT release aligns with and provides a mechanistic explanation for the documented benefits of aspirin in desensitized patients, as outlined in European treatment guidelines; thus, the antiplatelet strategy should be repurposed as a promising and rational therapeutic approach for type 2 inflammatory diseases. These findings underscore the therapeutic potential of targeting the 5-HT-TGM2-H3Q5Ser pathway, either alone or as an adjunct to biological therapies. Nevertheless, further in-depth exploration of its molecular mechanisms and optimization of drug design are needed to promote its clinical translation and application.

## Materials and methods

### Subjects

Patients with eCRS with or without asthma and neCRS were enrolled. CRS and asthma were diagnosed in accordance with international guidelines [74, 75]. eCRS was defined as an eosinophil count of 10 or more per high-power field (HPF, × 400 magnification) in NP tissue [74]. Healthy controls (HC) who underwent septoplasty for anatomical variations had no other sinus conditions. In this study, we collectively refer to all CRS cases as “CRS” without further stratification by nasal polyp status, as NP tissues were consistently used for tissue-based analyses across patient subgroups. NP tissues were collected from CRS patients, and middle/inferior turbinate mucosal tissues were obtained from HCs. All participants provided written informed consent under the Shanghai Sixth People’s Hospital IRB (protocol 2019-KY-039(K)). Owing to the limited sample availability, not all the samples were used in every experimental protocol. Further details are available in Table E1 in the Online Repository.

### Animals

*Tgm2*^−/−^, *Tph1*^−/−^ and *Tgm2*^fl/fl^; *Lyz2*-Cre (macrophage-specific *Tgm2* knockout) mice were generated by Cyagen Biotechnology Co., Ltd. (Suzhou, China) and bred under pathogen-free conditions. The mice were housed in a specific-pathogen-free animal facility under standard temperature and light control conditions. Six- to eight-week-old male mice were used in this experiment. All animal experiments were conducted in compliance with the guidelines of the Institutional Animal Care and Use Committee (IACUC) of Shanghai Sixth People’s Hospital (Shanghai, China) (No: 2022–0107).

### Single-cell RNA sequencing (scRNA-seq) analysis and visualization

Fresh nasal polyp tissues from eCRS patients were dissociated using the Multitissue Dissociation Kit 2 (Miltenyi Biotec, USA), followed by erythrocyte removal, viability assessment (Countstar® Rigel S2, Shanghai, China), and resuspension of viable cells (1 × 10^6^ cells/mL in 1×PBS and 0.04% bovine serum albumin). Single-cell capture, barcoding, and cDNA library preparation were performed using the SeekOne®MM microfluidic platform (SeekGene, China). Libraries were sequenced on an Illumina NovaSeq 6000 platform (150 bp paired-end). The raw sequencing data were processed with *CellRanger* (v7.1.0) for demultiplexing, alignment (GRCh38 reference), and UMI counting. Downstream single-cell analysis was performed using *Seurat* (v4.3.0), implementing standard workflows for dimensionality reduction, clustering, and cell type annotation. Differential gene expression between disease states was assessed using the Wilcoxon rank-sum test with thresholds of |log2FC| >0.25 and adjusted *p* value < 0.01. GSEA and GO analysis were performed with the *ClusterProfiler* package in *Bioconductor* under default parameters, and the results were visualized using the *enrichplot* package from the same framework. Pseudotime trajectory analysis was performed using the *monocle3* package.

Gene signature scores were calculated using the *AddModuleScore* function in *Seurat* for the following processes: classical activation (*TNF*, *IL1B*, *NOS2*, *CXCL9*, *CXCL10*, *CXCL11*, *STAT1*, *IRF5*, *CD86*, *HLA-DRA*, *IFI16*, *GBP1*, *GBP2*, and *VCAN*), alternative activation (*MRC1, CD163, ARG1, IL10, TGFB1, PPARG, CCL17, CCL22, TREM1, TREM2, F13A1, STAB1, and SPP1*), intermediate activation (*CD44, ITGAM, ITGAX, CD86, FBP1, TREM1, TREM2, CD52, FCER2, FCGR1A, and SLC17A11*), and ECM organization (*TGFB1, SMAD3, IGF1, FSTL1, MMP2, MMP9, MMP12, FLT1, MMP19, COL1A1, FN1, F13A1, TIMP1, and TIMP4*).

To construct a continuous CRS disease spectrum, we integrated publicly available scRNA-seq data from the National Genomics Data Center (accession HRA000772) with our own experimental datasets. These data were generated using 10x Genomics Chromium technology. Following standardized quality control, normalization, and log-transformation procedures, the Harmony linear batch-correction algorithm was applied to effectively remove batch effects across distinct cohorts while preserving true biological variations. Sample annotations were also harmonized across datasets: our local “eCRS” samples were merged with public eCRSwNP samples into the eCRSwNP group, while our “eCRSwAS” samples were designated as a separate group, eCRSwNP with asthma (eCRSwNPwAS). This integrated dataset was subsequently used for downstream dimensional reduction, clustering, and comparative expression trajectory analyses across multiple CRS subtypes.

### Single-molecular FISH to detect *FN1* mRNA in macrophages

FISH probes complementary to the *FN1* mRNA sequence were designed to cover the region of *FN1* (GD Pinpoease Biotech Co., Ltd.). FISH was performed using a PinpoRNATM RNA in situ hybridization kit according to the manufacturer’s instructions (GD Pinpoease Biotech Co. Ltd., PIF3000). Briefly, the paraffin sections were dewaxed and rehydrated, after which endogenous peroxidase activity was inhibited by Pre-A solution at room temperature. The target RNA molecules were exposed to protease treatment and hybridized with probes for 2 h at 40 °C. The signal was then amplified sequentially by reactions 1, 2, and 3. Finally, a tyramide fluorescent substrate (Runnerbio, AF647, 1:50) was added to the tissues, and the target RNA was then fluorescently labeled by a Tyramide signal amplification assay. The paraffin sections were then blocked with 1% BSA for 1 h at room temperature and incubated overnight at 4 °C with primary antibodies against TGM2 (Proteintech, 15100-1-AP, 1:200) and CD163 (Santa Cruz, sc-20066, 1:200), followed by incubation with a fluorescent secondary antibody for 2 hours at 37 °C in the dark. The nuclei were stained with DAPI for 15 min. Images were captured using a Carl Zeiss confocal microscope.

### Mouse primary peritoneal macrophage isolation

Mice from different genotypes were intraperitoneally injected with 2 mL of 4% Hanahan’s broth medium (Sigma‒Aldrich, H8032). After 3 days, peritoneal lavage was performed using 10 mL of cold PBS, and primary macrophages were isolated. Cells were plated in 12-well plates at 1 × 10⁶ cells/well in RPMI-1640 medium supplemented with 10% FBS and penicillin/streptomycin and then incubated for 2 hours at 37 °C with 5% CO₂. Nonadherent cells were removed by three PBS washes, and the adherent cells were considered primary peritoneal macrophages [76]. The cytokines and inhibitors used in our research are listed in Table E2.

### Bulk RNA sequencing (RNA-seq) analysis

Total RNA was extracted from primary peritoneal macrophages from wild-type or *Tgm2*^−/−^ mice, both with and without IL-13 stimulation, using TRIzol (ABclonal, RK30129). RNA-seq libraries were subsequently constructed, sequenced, and mapped. Differential gene expression analysis was performed using the DESeq R package (v1.18.0). Genes with a *p* value < 0.05 and an absolute |log2(fold change)| ≥ 2 were identified as significantly differentially expressed.

### Induction of type 2 inflammation in an animal model

House dust mites (1.25 μg/μl * 20 µl, i.n., 10 µl for each nose; GREER, XPB91D3A2.5) or an equivalent volume of PBS (20 µl, i.n., 10 µl for each nose) alone was administered intranasally following isoflurane anesthesia on a daily basis for the initial 10 days, and the mice were subsequently challenged with dust mites after a period of rest (Fig. 3A) [77]. Euthanasia occurred 24 h after the final dose. In some experiments, exogenous 5-HTP was administered intraperitoneally on days 1–10. ERW1041E, Epi, Ril, or Sar was administered intranasally on days 15–25.

### Tissue collection and processing

After the tracheas of the euthanized mice were cannulated, 1 mL of PBS was instilled into the lungs to retrieve the BLF. Afterward, a 22-gauge catheter was inserted through the tracheotomy hole into the posterior choana. One milliliter of PBS was instilled into the sinonasal cavity to collect the NLF. The protein levels of IL-5, IL-10, IL-13, IGF-1 and CCL-24 in the NLF and BLF were quantified by ELISA. Nasal and lung tissues were collected for subsequent western blotting and qRT‒PCR analysis.

### Histology and immunofluorescence

Fresh tissue samples were fixed in 4% paraformaldehyde (PFA) and embedded in paraffin. Thin sections (4 μm) were cut, rehydrated, and stained with hematoxylin and eosin (HE) to assess pathological features and inflammatory cell infiltration. The extent of cellular infiltration was graded on a scale of 0–4, with 0 indicating no infiltration and 4 indicating severe infiltration. Periodic acid–Schiff (PAS) and Giemsa–Wright staining were performed to determine the goblet cell and eosinophil counts, respectively. Sirius Red and Masson staining were used to assess collagen deposition. Three discrete tissue sections from each sample and 10 HPFs from each tissue section were randomly selected and analyzed by 2 independent physicians who were blinded to the clinical data as previously described.

For immunofluorescence, deparaffinized sections were blocked with 1% BSA for 1 h at room temperature, incubated overnight at 4 °C with a primary antibody, and treated with a fluorescent secondary antibody for 2 hours at 37 °C in the dark. The nuclei were stained with DAPI for 15 mins. Images were captured using a ZEISS LSM 710 META confocal laser-scanning microscope. The images were analyzed using ZEN 2011 software (Zeiss). The antibody details are listed in Table E3.

For multiplex immunofluorescence staining, the nasal polyp sample sections were dewaxed with conventional xylene and hydrated with gradient alcohol. mIHC of the tissue sections was performed using an eight-color multiplex immunofluorescence staining kit (AiFang Biological, AFIHC027). In accordance with the manufacturer’s instructions, the antigens were subjected to microwave repair. The sections were incubated with a 3% hydrogen peroxide solution at room temperature for 15 mins and then with 10% goat serum for blocking for 15 mins. Primary antibody A was added, and the samples were incubated overnight at 4 °C and then washed three times with PBST. Polymer-HRP anti-mouse/rabbit universal secondary antibody IgG (AiFang Biological, AFIHC001) was added, and the sections were incubated at room temperature for 30 mins. The sections were subsequently washed with PBST, after which the TYR fluorescent dye was added, and the mixture was allowed to react for 8 min, followed by three washes with PBST. Antibody A was removed by microwave treatment, and the sections were washed three times with PBST. After the sections were blocked with goat serum, primary antibody B was added, and all the antigens, including TGM2 (Santa Cruz, sc-73612, 1:2000), CD163 (Santa Cruz, sc-20066, 1:4000), CD41 (Aifang Biological, AFRM81216, 1:3000), H3Q5Ser (ABclonal, A20210, 1:2000), FN1 (Abmart, T59537, 1:4000), GATA3 (AiFang Biological, AF20240, 1:2000), and KLRG1 (Proteintech, 84785-4-RR, 1:3000), were completely visualized. DAPI staining solution was added, and the sections were incubated at room temperature in the dark for 10 min, followed by three washes with PBST. The sections were mounted with anti-fluorescence quenching mounting medium, and the mIHC slides were imaged using an eight-channel fluorescence digital slide scanner (model AF-KL-20-8; AiFang Biological, China).

### RNA preparation, reverse transcription–PCR, and quantitative real-time PCR

Total RNA from human tissues and mouse macrophages, noses, or lungs was extracted using TRIzol reagent (ABclonal, RK30129) following the manufacturer’s instructions. Reverse transcription was conducted with a Color Reverse Transcription Kit (EZBioscience, A0010CGQ-L), and qRT‒PCR was performed using 2× EZ Color SYBR Green qPCR Master Mix (EZBioscience, CQ20). Relative mRNA expression levels were calculated using the 2^(−△△Ct)^ method, with glucuronidase beta (GUSB) as the reference gene. The primer sequences are listed in Tables E4–5.

### Western blotting

Briefly, proteins were extracted from tissues and cells. The protein concentration was determined before the proteins were equally loaded and separated as described in polyacrylamide gels. The proteins were then transferred to a nitrocellulose filter membrane. The membranes were blocked in 5% non-fat milk and then incubated with diluted primary antibodies at 4 °C overnight. The antibodies used are listed in Table E3. HRP-conjugated secondary antibodies were then applied to the membrane, and the western blotting signal was detected using autoradiographic film after incubation with an Omni-ECL™ Femto Light Chemiluminescence Kit (Epizyme, SQ201).

### ELISA

ELISAs were performed using a selection of ELISA kits in strict accordance with the manufacturer’s protocols. Optical density (OD) readings at 450 nm, along with background correction at 630 nm, were measured using a Bio Tek Synergy^TM^ H1 microplate reader (Biotek, USA). Kits for the ELISA experiments are listed in Table E6.

### Flow cytometry

A total of 100 μL of cell suspension, which had been crushed and filtered to adjust the cell concentration, was mixed with 2.5 μL of CD45-PE-CY7 (BioLegend, 103114, 1:200), 2.5 μL of CD11b-BV605 (BioLegend, 101257, 1:200), and 2.5 μL of anti-CD14-APC (BioLegend, 325608, 1:200) flow antibodies for surface staining. The mixture was incubated on ice in the dark for 20 min. An aliquot of 1 mL of 1× permeabilization buffer was added to each tube before centrifugation at 350 × *g* at room temperature for 5 min, and the supernatant was carefully discarded. The cells were then mixed with 2.5 μL of TGM2-FITC/AL488 (Proteintech, CL488-68006, 1:200) flow antibodies for intracellular staining for 30 min at 4 °C. An aliquot of 1 ml of 1 × flow cytometry staining buffer was added to each tube for staining detection within 3 h. Flow cytometry data were collected using BD Canto plus (version 8) and analyzed with FlowJo (version 10).

### CUT&Tag assay

Approximately 10^5^ cells were collected for each sample, and primary and secondary antibodies were applied sequentially. The primary antibodies used were rabbit anti-H3Q5Ser (Sigma‒Aldrich, ABE1791, 1:50), anti-H3K4Me3 (ABclonal, A22264, 1:50), H3K9Ac (ABclonal, A21107, 1:50), H3K27Ac (ABclonal, A22264, 1:50) and mouse anti-TGM2 (Santa Cruz, sc-73612, 1:50). The secondary antibody was goat anti-rabbit IgG (H + L) (ABclonal, AS070, 1:100). The pAG-Tn5 transposase, supplied by ABclonal, was utilized to construct the CUT&Tag library according to the protocol specified in the CUT&Tag Assay Kit (pAG-Tn5) for Illumina (ABclonal, RK20265). The finalized library was subsequently processed for quantification, sequencing, and alignment. Data were processed and presented using the Integrative Genomics Viewer (IGV, version 2). The primers used for cut-tag qPCR are listed in Table E7.

### Platelet isolation

Whole blood was collected from the abdominal aortas of anesthetized wild-type and *Tph1*^*−/−*^ mice (8–12 weeks old) using a 2.5 mL syringe prefilled with 10% white’s buffer. The blood was gently mixed and transferred to a 15 mL tube and then immediately incubated in a 37 °C water bath to prevent premature platelet activation. Afterward, 0.9% saline, prostaglandin E1 (0.1 μg/mL), and apyrase (1 U/mL) were added, and the mixture was gently inverted. Platelet-rich plasma (PRP) was isolated by centrifugation at 1200 rpm for 10 min. The PRP was transferred to a new tube and supplemented with additional apyrase (1 U/mL) and EDTA (5 mmol/L). After a second centrifugation step at 2100 rpm for 10 min, the supernatant was discarded, and the platelet pellet was resuspended in 200 μL of modified Tyrode’s buffer. The platelet concentration was quantified using an automated cell counter (HEMAVET®950FS, US). Primary peritoneal macrophages were isolated from wild-type and *Tgm2*^*−/−*^ mice and then stimulated for 6 h with 3 × 108/mL platelets isolated from wild-type and *Tph1*^*−/−*^ mice.

### Statistical analysis

No statistical methods were applied to determine the sample size prior to the experiment. The number of samples included in each analysis is specified in the figure legends. For the scRNA-seq and RNA-seq data, statistical analysis and figure generation were conducted using R version 3.6.1 (R Foundation for Statistical Computing).

For the experimental data, statistical analyses and graphical representation were carried out using GraphPad Prism 10. Descriptive statistics for categorical and continuous variables are presented as frequencies (percentages) and means ± standard errors of the means (SEMs), respectively. The detailed statistical methods are listed in the figure legends. Statistical significance was defined as a *p* value of less than 0.05.

## Supplementary information

Supplementary Text and Figures

## Data Availability

The raw sequencing data from the scRNA-seq of human nasal polyp samples generated in this study have been deposited in the National Omics Data Encyclopedia (NODE) repository under the accession code OEP00006178. The raw sequencing data from bulk RNA-seq, CUT&Tag, and ATAC-sequencing analyses of mouse macrophages in this study are deposited in the Gene Expression Omnibus (GEO) under the accession codes GSE295022, GSE295021, and GSE299151, respectively. The raw sequencing data from the scRNA-seq analysis of the mouse nasal mucosa in this study are deposited in GEO under the accession code GSE298738. The publicly available scRNA transcriptomic datasets from different CRS subtypes used for data mining and validation in this study were obtained from the National Genomics Data Center (NGDC; https://ngdc.cncb.ac.cn/gsa-human/browse/HRA000772), which has been previously published. All other data are available in the article and its Supplementary files or from the corresponding author upon request.

## Declarations

Declarations During the preparation of this work, the authors used DeepSeek to improve the language. After using this tool, the authors reviewed and edited the content as needed and take full responsibility for the content of this publication.

## References

[1] Kolkhir P, Akdis CA, Akdis M, Bachert C, Bieber T, Canonica GW, et al. Type 2 chronic inflammatory diseases: targets, therapies and unmet needs. Nat Rev Drug Discov. 2023;22:743–67. 10.1038/s41573-023-00750-1 37528191

[2] Ogulur I, Mitamura Y, Yazici D, Pat Y, Ardicli S, Li M, et al. Type 2 immunity in allergic diseases. Cell Mol Immunol. 2025;22:211–42. 10.1038/s41423-025-01261-2 PMC11868591 39962262

[3] Kratchmarov R, Dharia T, Buchheit K. Clinical efficacy and mechanisms of biologics for chronic rhinosinusitis with nasal polyps. J Allergy Clin Immunol. 2025;155:1401–10. 10.1016/j.jaci.2025.03.011 PMC12058411 40132672

[4] Hopkins C. Chronic rhinosinusitis with nasal polyps. N Engl J Med. 2019;381:55–63. 10.1056/NEJMcp1800215 31269366

[5] Hoggard M, Wagner Mackenzie B, Jain R, Taylor MW, Biswas K, Douglas RG. Chronic rhinosinusitis and the evolving understanding of microbial ecology in chronic inflammatory mucosal disease. Clin Microbiol Rev. 2017;30:321–48. 10.1128/CMR.00060-16 PMC5217796 27903594

[6] Maselli DJ, Sherratt J, Adams SG. Comorbidities and multimorbidity in asthma. Curr Opin Pulm Med. 2025;31:270–8. 10.1097/MCP.0000000000001162 40047208

[7] Ledford DK, Kim T-B, Ortega VE, Cardet JC. Asthma and respiratory comorbidities. J Allergy Clin Immunol. 2025;155:316–26. 10.1016/j.jaci.2024.11.006 PMC12180284 39542142

[8] Elahi S, Peters AT, Kato A, Stevens WW. Clinical and mechanistic advancements in aspirin exacerbated respiratory disease. J Allergy Clin Immunol. 2025;155:1411–9. 10.1016/j.jaci.2025.03.006 PMC12058404 40113018

[9] Gordon S. Alternative activation of macrophages. Nat Rev Immunol. 2003;3:23–35. 10.1038/nri978 12511873

[10] Gordon S, Martinez FO. Alternative activation of macrophages: mechanism and functions. Immunity. 2010;32:593–604. 10.1016/j.immuni.2010.05.007 20510870

[11] Murray PJ, Wynn TA. Protective and pathogenic functions of macrophage subsets. Nat Rev Immunol. 2011;11:723–37. 10.1038/nri3073 PMC3422549 21997792

[12] Nishide M, Shimagami H, Kumanogoh A. Single-cell analysis in rheumatic and allergic diseases: insights for clinical practice. Nat Rev Immunol. 2024;24:781–97. 10.1038/s41577-024-01043-3 38914790

[13] Van Dyken SJ, Locksley RM. Interleukin-4- and interleukin-13-mediated alternatively activated macrophages: roles in homeostasis and disease. Annu Rev Immunol. 2013;31:317–43. 10.1146/annurev-immunol-032712-095906 PMC3606684 23298208

[14] Rabe KF, Rennard S, Martinez FJ, Celli BR, Singh D, Papi A, et al. Targeting type 2 inflammation and epithelial alarmins in chronic obstructive pulmonary disease: a biologics outlook. Am J Respir Crit Care Med. 2023;208:395–405. 10.1164/rccm.202303-0455CI 37348121

[15] Ryan DG, O’Neill LAJ. Krebs cycle reborn in macrophage immunometabolism. Annu Rev Immunol. 2020;38:289–313. 10.1146/annurev-immunol-081619-104850 31986069

[16] Odegaard JI, Chawla A. Alternative macrophage activation and metabolism. Annu Rev Pathol. 2011;6:275–97. 10.1146/annurev-pathol-011110-130138 PMC3381938 21034223

[17] Chawla A, Nguyen KD, Goh YPS. Macrophage-mediated inflammation in metabolic disease. Nat Rev Immunol. 2011;11:738–49. 10.1038/nri3071 PMC3383854 21984069

[18] Wang W, Xu Y, Wang L, Zhu Z, Aodeng S, Chen H, et al. Single-cell profiling identifies mechanisms of inflammatory heterogeneity in chronic rhinosinusitis. Nat Immunol. 2022;23:1484–94. 10.1038/s41590-022-01312-0 36138182

[19] Imoto Y, Kato A, Takabayashi T, Stevens W, Norton JE, Suh LA, et al. Increased thrombin-activatable fibrinolysis inhibitor levels in patients with chronic rhinosinusitis with nasal polyps. J Allergy Clin Immunol. 2019;144:1566–1574.e6. 10.1016/j.jaci.2019.08.040 PMC6900453 31562871

[20] Al-Amer OM. The role of thrombin in hemostasis. Blood Coagul Fibrinolysis. 2022;33:145–8. 10.1097/MBC.0000000000001130 35239615

[21] Takabayashi T, Schleimer RP. Formation of nasal polyps: The roles of innate type 2 inflammation and deposition of fibrin. J Allergy Clin Immunol. 2020;145:740–50. 10.1016/j.jaci.2020.01.027 PMC7263055 32145873

[22] Yue M, Hu M, Fu F, Ruan H, Wu C. Emerging roles of platelets in allergic asthma. Front Immunol. 2022;13:846055. 10.3389/fimmu.2022.846055 PMC9010873 35432313

[23] Gremmel T, Frelinger AL, Michelson AD. Platelet physiology. Semin Thromb Hemost. 2016;42:191–204. 10.1055/s-0035-1564835 26926581

[24] Pavón-Romero GF, Serrano-Pérez NH, García-Sánchez L, Ramírez-Jiménez F, Terán LM. Neuroimmune pathophysiology in asthma. Front Cell Dev Biol. 2021;9:663535. 10.3389/fcell.2021.663535 PMC8155297 34055794

[25] Shajib MS, Khan WI. The role of serotonin and its receptors in activation of immune responses and inflammation. Acta Physiol). 2015;213:561–74. 10.1111/apha.12430 25439045

[26] Sandyk R. Serotonergic neuronal sprouting as a potential mechanism of recovery in multiple sclerosis. Int J Neurosci. 1999;97:131–8. 10.3109/00207459908994307 10681122

[27] Karmakar S, Lal G. Role of serotonin receptor signaling in cancer cells and anti-tumor immunity. Theranostics. 2021;11:5296–312. 10.7150/thno.55986 PMC8039959 33859748

[28] Zhang Y, Wang Y. The dual roles of serotonin in antitumor immunity. Pharmacol Res. 2024;205:107255. 10.1016/j.phrs.2024.107255 38862071

[29] Hwang YK, Oh JS. Interaction of the vagus nerve and serotonin in the gut-brain axis. Int J Mol Sci. 2025;26:1160. 10.3390/ijms26031160 PMC11818468 39940928

[30] Farrelly LA, Thompson RE, Zhao S, Lepack AE, Lyu Y, Bhanu NV, et al. Histone serotonylation is a permissive modification that enhances TFIID binding to H3K4me3. Nature. 2019;567:535–9. 10.1038/s41586-019-1024-7 PMC6557285 30867594

[31] Dong R, Wang T, Dong W, Zhang H, Li Y, Tao R, et al. TGM2-mediated histone serotonylation promotes HCC progression via MYC signaling pathway. J Hepatol. 2025;83:105–18. 10.1016/j.jhep.2024.12.038 39788430

[32] Chen H-C, He P, McDonald M, Williamson MR, Varadharajan S, Lozzi B, et al. Histone serotonylation regulates ependymoma tumorigenesis. Nature. 2024;632:903–10. 10.1038/s41586-024-07751-z PMC11951423 39085609

[33] Sardar D, Cheng Y-T, Woo J, Choi D-J, Lee Z-F, Kwon W, et al. Induction of astrocytic Slc22a3 regulates sensory processing through histone serotonylation. Science. 2023;380:eade0027. 10.1126/science.ade0027 PMC10874521 37319217

[34] Al-Kachak A, Di Salvo G, Fulton SL, Chan JC, Farrelly LA, Lepack AE, et al. Histone serotonylation in dorsal raphe nucleus contributes to stress- and antidepressant-mediated gene expression and behavior. Nat Commun. 2024;15:5042. 10.1038/s41467-024-49336-4 PMC11176395 38871707

[35] Kristjansson RP, Benonisdottir S, Davidsson OB, Oddsson A, Tragante V, Sigurdsson JK, et al. A loss-of-function variant in ALOX15 protects against nasal polyps and chronic rhinosinusitis. Nat Genet. 2019;51:267–76. 10.1038/s41588-018-0314-6 30643255

[36] Semple JW, Italiano JE, Freedman J. Platelets and the immune continuum. Nat Rev Immunol. 2011;11:264–74. 10.1038/nri2956 21436837

[37] Jin J, Xu F, Zhang Y, Guan J, Liang X, Zhang Y, et al. Renal ischemia/reperfusion injury in rats is probably due to the activation of the 5-HT degradation system in proximal renal tubular epithelial cells. Life Sci. 2021;285:120002. 10.1016/j.lfs.2021.120002 34599937

[38] Maher-Edwards G, De’Ath J, Barnett C, Lavrov A, Lockhart A. A 24-week study to evaluate the effect of rilapladib on cognition and cerebrospinal fluid biomarkers of Alzheimer’s disease. Alzheimers Dement. 2015;1:131–40. 10.1016/j.trci.2015.06.003 PMC5975052 29854933

[39] Nogueira L, Ramirez-Sanchez I, Perkins GA, Murphy A, Taub PR, Ceballos G, et al. (-)-Epicatechin enhances fatigue resistance and oxidative capacity in mouse muscle. J Physiol. 2011;589:4615–31. 10.1113/jphysiol.2011.209924 PMC3208228 21788351

[40] Shinde AV, Su Y, Palanski BA, Fujikura K, Garcia MJ, Frangogiannis NG. Pharmacologic inhibition of the enzymatic effects of tissue transglutaminase reduces cardiac fibrosis and attenuates cardiomyocyte hypertrophy following pressure overload. J Mol Cell Cardiol. 2018;117:36–48. 10.1016/j.yjmcc.2018.02.016 PMC5892840 29481819

[41] Qin H-Y, Xavier Wong HL, Zang K-H, Li X, Bian Z-X. Enterochromaffin cell hyperplasia in the gut: factors, mechanism and therapeutic clues. Life Sci. 2019;239:116886. 10.1016/j.lfs.2019.116886 31678286

[42] Steingold K, Stumpf P, Kreiner D, Liu HC, Navot D, Rosenwaks Z. Estradiol and progesterone replacement regimens for the induction of endometrial receptivity. Fertil Steril. 1989;52:756–60. 10.1016/s0015-0282(16)61027-0 2806616

[43] Lund ML, Egerod KL, Engelstoft MS, Dmytriyeva O, Theodorsson E, Patel BA, et al. Enterochromaffin 5-HT cells - a major target for GLP-1 and gut microbial metabolites. Mol Metab. 2018;11:70–83. 10.1016/j.molmet.2018.03.004 PMC6001397 29576437

[44] Haq S, Wang H, Grondin J, Banskota S, Marshall JK, Khan II, et al. Disruption of autophagy by increased 5-HT alters gut microbiota and enhances susceptibility to experimental colitis and Crohn’s disease. Sci Adv. 2021;7:eabi6442. 10.1126/sciadv.abi6442 PMC8570609 34739317

[45] Tian J, Zhu T, Liu J, Guo Z, Cao X. Platelets promote allergic asthma through the expression of CD154. Cell Mol Immunol. 2015;12:700–7. 10.1038/cmi.2014.111 PMC4716619 25418472

[46] Nakanishi T, Inaba M, Inagaki-Katashiba N, Tanaka A, Vien PTX, Kibata K, et al. Platelet-derived RANK ligand enhances CCL17 secretion from dendritic cells mediated by thymic stromal lymphopoietin. Platelets. 2015;26:425–31. 10.3109/09537104.2014.920081 24867354

[47] Ulfman LH, Joosten DPH, van Aalst CW, Lammers J-WJ, van de Graaf EA, Koenderman L, et al. Platelets promote eosinophil adhesion of patients with asthma to endothelium under flow conditions. Am J Respir Cell Mol Biol. 2003;28:512–9. 10.1165/rcmb.4806 12654641

[48] Pitchford SC, Momi S, Giannini S, Casali L, Spina D, Page CP, et al. Platelet P-selectin is required for pulmonary eosinophil and lymphocyte recruitment in a murine model of allergic inflammation. Blood. 2005;105:2074–81. 10.1182/blood-2004-06-2282 15528309

[49] Yang G, Wu G, Yao W, Guan L, Geng X, Liu J, et al. 5-HT is associated with the dysfunction of regulating T cells in patients with allergic rhinitis. Clin Immunol. 2022;243:109101. 10.1016/j.clim.2022.109101 36029976

[50] Abdala-Valencia H, Berdnikovs S, McCary CA, Urick D, Mahadevia R, Marchese ME, et al. Inhibition of allergic inflammation by supplementation with 5-hydroxytryptophan. Am J Physiol Lung Cell Mol Physiol. 2012;303:L642–L660. 10.1152/ajplung.00406.2011 PMC3469632 22842218

[51] Walker MT, Bloodworth JC, Kountz TS, McCarty SL, Green JE, Ferrie RP, et al. 5-HTP inhibits eosinophilia via intracellular endothelial 5-HTRs; SNPs in 5-HTRs associate with asthmatic lung function. Front Allergy. 2024;5:1385168. 10.3389/falgy.2024.1385168 PMC11153829 38845678

[52] Shajib MS, Wang H, Kim JJ, Sunjic I, Ghia J-E, Denou E, et al. Interleukin 13 and serotonin: linking the immune and endocrine systems in murine models of intestinal inflammation. PLoS ONE. 2013;8:e72774. 10.1371/journal.pone.0072774 PMC3755966 24015275

[53] Turner EH, Loftis JM, Blackwell AD. Serotonin a la carte: supplementation with the serotonin precursor 5-hydroxytryptophan. Pharmacol Ther. 2006;109:325–38. 10.1016/j.pharmthera.2005.06.004 16023217

[54] Huang SC-C, Smith AM, Everts B, Colonna M, Pearce EL, Schilling JD, et al. Metabolic reprogramming mediated by the mTORC2-IRF4 signaling axis is essential for macrophage alternative activation. Immunity. 2016;45:817–30. 10.1016/j.immuni.2016.09.016 PMC5535820 27760338

[55] Martinez FO, Helming L, Milde R, Varin A, Melgert BN, Draijer C, et al. Genetic programs expressed in resting and IL-4 alternatively activated mouse and human macrophages: similarities and differences. Blood. 2013;121:e57–69. 10.1182/blood-2012-06-436212 23293084

[56] Zhou Z-R, Fang S-B, Liu X-Q, Li C-G, Xie Y-C, He B-X, et al. Serum amyloid A1 induced dysfunction of airway macrophages via CD36 pathway in allergic airway inflammation. Int Immunopharmacol. 2024;142:113081. 10.1016/j.intimp.2024.113081 39244902

[57] Silverstein RL, Febbraio M. CD36, a scavenger receptor involved in immunity, metabolism, angiogenesis, and behavior. Sci Signal. 2009;2:re3. 10.1126/scisignal.272re3 PMC2811062 19471024

[58] Chen Y, Zhang J, Cui W, Silverstein RL. CD36, a signaling receptor and fatty acid transporter that regulates immune cell metabolism and fate. J Exp Med. 2022;219:e20211314. 10.1084/jem.20211314 PMC9022290 35438721

[59] de Las Casas-Engel M, Corbí AL. Serotonin modulation of macrophage polarization: inflammation and beyond. Adv Exp Med Biol. 2014;824:89–115. 10.1007/978-3-319-07320-0_9 25038996

[60] Poole LG, Kopec AK, Flick MJ, Luyendyk JP. Cross-linking by tissue transglutaminase-2 alters fibrinogen-directed macrophage proinflammatory activity. J Thromb Hemost. 2022;20:1182–92. 10.1111/jth.15670 PMC9035112 35158413

[61] Tatsukawa H, Hitomi K. Role of transglutaminase 2 in cell death, survival, and fibrosis. Cells. 2021;10:1842. 10.3390/cells10071842 PMC8307792 34360011

[62] Fuchshofer R, Birke M, Welge-Lussen U, Kook D, Lütjen-Drecoll E. Transforming growth factor-beta 2 modulated extracellular matrix component expression in cultured human optic nerve head astrocytes. Investig Ophthalmol Vis Sci. 2005;46:568–78. 10.1167/iovs.04-0649 15671284

[63] Dou F, Liu Q, Lv S, Xu Q, Wang X, Liu S, et al. FN1 and TGFBI are key biomarkers of macrophage immune injury in diabetic kidney disease. Medicine. 2023;102:e35794. 10.1097/MD.0000000000035794 PMC10637504 37960829

[64] Ma Z, Zhou X, Jia W, Tan X, Huang X, Wang J, et al. Setdb1 ablation in macrophages attenuates fibrosis in heart allografts. Proc Natl Acad Sci USA. 2025;122:e2424534122. 10.1073/pnas.2424534122 PMC12232555 40553495

[65] Wang J, Zhang L, Luo L, He P, Xiong A, Jiang M, et al. Characterizing cellular heterogeneity in fibrotic hypersensitivity pneumonitis by single-cell transcriptional analysis. Cell Death Discov. 2022;8:38. 10.1038/s41420-022-00831-x PMC8795750 35091537

[66] Szczesny B, Boorgula MP, Chavan S, Campbell M, Johnson RK, Kammers K, et al. Multiomics in nasal epithelium reveals three axes of dysregulation for asthma risk in the African Diaspora populations. Nat Commun. 2024;15:4546. 10.1038/s41467-024-48507-7 PMC11133339 38806494

[67] Wells SB, Rainbow DB, Mark M, Szabo PA, Ergen C, Caron DP, et al. Multimodal profiling reveals tissue-directed signatures of human immune cells altered with age. Nat Immunol. 2025;26:1612–25. 10.1038/s41590-025-02241-4 PMC12396968 40804529

[68] Zheng Q, Weekley BH, Vinson DA, Zhao S, Bastle RM, Thompson RE, et al. Bidirectional histone monoaminylation dynamics regulate neural rhythmicity. Nature. 2025;637:974–82. 10.1038/s41586-024-08371-3 PMC11754111 39779849

[69] Zhang Y, Li J, Wang M, Li X, Yan B, Liu J et al. Stapokibart for moderate-to-severe seasonal allergic rhinitis: a randomized phase 3 trial. Nat Med. 2025. 10.1038/s41591-025-03651-5. PMC12283386 40186079

[70] Bachert C, Han JK, Desrosiers M, Hellings PW, Amin N, Lee SE, et al. Efficacy and safety of dupilumab in patients with severe chronic rhinosinusitis with nasal polyps (LIBERTY NP SINUS-24 and LIBERTY NP SINUS-52): results from two multicenter, randomized, double-blind, placebo-controlled, parallel-group phase 3 trials. Lancet. 2019;394:1638–50. 10.1016/S0140-6736(19)31881-1 31543428

[71] Lipworth BJ, Han JK, Desrosiers M, Hopkins C, Lee SE, Mullol J, et al. Tezepelumab in adults with severe chronic rhinosinusitis with nasal polyps. N Engl J Med. 2025;392:1178–88. 10.1056/NEJMoa2414482 40106374

[72] Han JK, Bachert C, Fokkens W, Desrosiers M, Wagenmann M, Lee SE, et al. Mepolizumab for chronic rhinosinusitis with nasal polyps (SYNAPSE): a randomized, double-blind, placebo-controlled, phase 3 trial. Lancet Respir Med. 2021;9:1141–53. 10.1016/S2213-2600(21)00097-7 33872587

[73] Siegel M, Khosla C. Transglutaminase 2 inhibitors and their therapeutic role in disease states. Pharmacol Ther. 2007;115:232–45. 10.1016/j.pharmthera.2007.05.003 PMC1975782 17582505

[74] Fokkens WJ, Lund VJ, Hopkins C, Hellings PW, Kern R, Reitsma S, et al. European position paper on rhinosinusitis and nasal polyps 2020. Rhinology. 2020;58:1–464. 10.4193/Rhin20.600 32077450

[75] Venkatesan P. 2023 GINA report for asthma. Lancet Respir Med. 2023;11:589. 10.1016/S2213-2600(23)00230-8 37302397

[76] Chen J, Tang F, Li H, Wu X, Yang Y, Liu Z, et al. *Mycobacterium tuberculosis* suppresses APLP2 expression to enhance its survival in macrophage. Int Immunopharmacol. 2023;124:111058. 10.1016/j.intimp.2023.111058 37844466

[77] Raundhal M, Morse C, Khare A, Oriss TB, Milosevic J, Trudeau J, et al. High IFN-γ and low SLPI mark severe asthma in mice and humans. J Clin Investig. 2015;125:3037–50. 10.1172/JCI80911 PMC4563754 26121748

