# Author Correction: Platelet-derived serotonin epigenetically programs macrophage alternative activation to orchestrate type 2 airway inflammation

**DOI:** 10.1038/s41423-026-01465-0

**Published:** 2026-09-01

**Authors:** Ru Tang, Guofang Xia, Ying Zhu, Jinhong Shen, Zhihan Liu, Song Mao, Jiayao Zhou, Yuelong Gu, Shilei Pu, Zhipeng Li, Hai Lin, Congfeng Xu, Hongqing Xu, Feng Zhang, Yongxu Zhao, Shankai Yin, Weitian Zhang, Feng Liu

**Affiliations:** 1 Department of Otolaryngology Head and Neck Surgery, Otolaryngology Institute of Shanghai Jiao Tong University, Shanghai Sixth People’s Hospital Affiliated with Shanghai Jiao Tong University School of Medicine, Shanghai, China; 2 National Key Specialty of Skull Base Diseases Affiliated with Shanghai Sixth People’s Hospital, Shanghai, China; 3 Center for Sleep Monitoring and Respiratory Therapy Technology, Institute of Medical Equipment and Technology, Shanghai Jiao Tong University School of Medicine, Shanghai, China; 4 Department of Cardiology, Shanghai Sixth People’s Hospital Affiliated with Shanghai Jiao Tong University School of Medicine, Shanghai, China; 5 Department of Otolaryngology Head and Neck Surgery, Shanghai Children’s Hospital, Shanghai Jiao Tong University, Shanghai, China; 6 Department of Cardiovascular Medicine, Quzhou Affiliated Hospital of Wenzhou Medical University, Quzhou, China; 7 Department of Respiratory and Critical Care, Quzhou Key Laboratory of Aging Biology and Longevity Medicine, Zhejiang-Germany Joint Laboratory on Lung Injury Repair and Regeneration, Quzhou Affiliated Hospital of Wenzhou Medical University, Quzhou, China; 8 Shanghai Institute of Materia Medica, Chinese Academy of Sciences, Shanghai, China; 9 Shandong Laboratory of Yantai Drug Discovery, Bohai Rim Advanced Research Institute for Drug Discovery, Yantai, China

**Keywords:** Chronic inflammation, Monocytes and macrophages

Correction to: *Cellular & Molecular Immunology* https://doi.org/10.1038/s41423-026-01455-2, published online 30 July 2026

In this article the author’s name Jinhong Shen was incorrectly written as Jinhong Sheng. The original article has been corrected.

