# Supplementary material for: Platelet-derived serotonin epigenetically programs macrophage alternative activation to orchestrate type 2 airway inflammation
Source: Cell Mol Immunol. 2026 Jul 30;23(10):1368–86. doi: 10.1038/s41423-026-01455-2 (PMC13624266; doi:10.1038/s41423-026-01455-2)

Online Repository

Platelet-Derived Serotonin Epigenetically Programs Macrophage Alternative Activation to Orchestrate Type 2 Airway Inflammation

Authors

Ru Tang [1,2,3], Guofang Xia[4], Ying Zhu [1,2,3], Jinhong Sheng[1,2,3], Zhihan Liu[1,2,3], Song Mao [1,2], Jiayao Zhou [1,2], Yuelong Gu [1,2], Shilei Pu [5], Zhipeng Li [1,2], Hai Lin [1,2], Congfeng Xu[4], Hongqing Xu [6], Feng Zhang [7], Yongxu Zhao [8,9]*, Shankai Yin [1,2,3]*, Weitian Zhang [1,2,3]*, Feng Liu [1,2,3]*

Affiliation

[1] Department of Otolaryngology Head and Neck Surgery, Otolaryngology Institute of Shanghai Jiao Tong University, Shanghai Sixth People's Hospital Affiliated to Shanghai Jiao Tong University School of Medicine, Shanghai 200233, China

[2] National Key Specialty of Skull Base Diseases Affiliated to Shanghai Sixth People's Hospital, Shanghai 200233, China

[3] Center for Sleep Monitoring and Respiratory Therapy Technology, Institute of Medical Equipment and Technology, Shanghai Jiao Tong University School of Medicine, Shanghai, China

[4] Department of Cardiology, Shanghai Sixth People's Hospital Affiliated to Shanghai Jiao Tong University School of Medicine, Shanghai 200233, China

[5] Department of Otolaryngology Head and Neck Surgery, Shanghai Children's Hospital, Shanghai Jiao Tong University, Shanghai, China

[6] Department of Cardiovascular Medicine, Quzhou Affiliated Hospital of Wenzhou Medical University, Quzhou 324000, China

[7] Department of Respiratory and Critical Care, Quzhou Affiliated Hospital of Wenzhou Medical University, Quzhou 324000, China

[8] Shanghai Institute of Materia Medica, Chinese Academy of Sciences, Shanghai 201203, China

[9] Shandong Laboratory of Yantai Drug Discovery, Bohai Rim Advanced Research Institute for Drug Discovery, Yantai, Shandong 264117, China

Correspondence

Feng Liu: (liufeng@sibs.ac.cn); Weitian Zhang: (drzhangwt@163.com); Shankai Yin: (skyin@sjtu.edu.cn); and Yongxu Zhao: (zhaoyongxu@simm.ac.cn);

Methods

Subjects sample collection and analysis

This study was approved by the Ethics Committee of Shanghai Jiao Tong University School of Medicine Affiliated Sixth People's Hospital (protocol 2019-KY-039(K)) and was conducted with written informed consent from each patient. A total of 145 individuals including 46 patients with eosinophilic chronic rhinosinusitis with nasal polyps (eCRS), 16 patients with eCRS with asthma (eCRSwAS), 37 patients with noneosinophilic chronic rhinosinusitis with nasal polyps (neCRS), and 46 healthy controls (HC) undergoing septoplasty because of anatomic variations and without other sinonasal diseases were enrolled in this study. All experiments were performed in accordance with the approved guidelines and regulations. The diagnosis of chronic rhinosinusitis with nasal polyps was made according to the European Position Paper on Rhinosinusitis and Nasal Polyps 2020.[1] eCRS was defined as eCRS was defined as an eosinophil count of 10 or more per high-power field in nasal polyp tissue.[2] The diagnosis of asthma was based on disease history and airway responsiveness test according to Global Initiative for Asthma guideline.[3] Allergic rhinitis (AR) was diagnosed based on the International consensus statement on allergy and rhinology: Allergic rhinitis 2023.[4] Patients with antrochoanal polyps, cystic fibrosis, pregnancy, primary ciliary dyskinesia, systemic vasculitis, fungal sinusitis, acute upper respiratory tract infection, acute asthma episode within 4 weeks of entering the study, and under immunotherapy were excluded from the study. Patients with

aspirin-exacerbated respiratory disease were excluded. Oral and intranasal steroids were discontinued at least 3 months and 1 month before the surgery, respectively. Antileukotrienes were discontinued at least 1 month before inclusion.

Nasal polyp tissues and middle/inferior turbinate mucosal tissues were obtained during surgery from chronic rhinosinusitis with nasal polyps (CRS) and HC, respectively. Tissue samples were used for histology, immunofluorescence, western blotting, qRT-PCR, enzyme-linked immunosorbent assay (ELISA), and single-cell RNA sequencing. Demographic and clinical characteristics of CRS patients and HC individuals were summarized in **Table E1**. Not all subjects were included in every experiment protocol because of the limited quantity of samples. The numbers of samples for every experiment were indicated in the figure legends.

ELISA

ELISAs were performed using a selection of ELISA kits (**Table E6**) in strict accordance with the manufacturer's protocols. Briefly, standard solutions and samples were pipetted into 96-well plates pre-coated with capture antibodies. The plates were then incubated at 37 °C for 2 hours. After incubation, the wells were emptied, and the plates underwent four cycles of washing using the provided wash buffer. Next, an enzyme-conjugated detection antibody was added to the wells, followed by a 1-hour incubation at 37 °C. Subsequently, the avidin-biotin-peroxidase complex was introduced into each well, with an additional incubation period of 30 minutes at the same temperature. After another series of four washes, a substrate solution for color development was added, and the plates were incubated in the dark at 37 °C for 15 minutes. The enzymatic reaction was terminated by adding a stop solution, and the optical density (OD) at 450 nm, with background correction at 630 nm, was promptly measured Bio Tek Synergy^{TM} H1 microplate reader (Biotek, USA).

Molecular Docking Analysis

Molecular docking was performed to investigate the potential interactions between 5-HT and the scavenger receptors CD36 and OLR1. The three-dimensional structure of the CD36 ectodomain was obtained from the Protein Data Bank (PDB ID: 5LGD).

For OLR1, the crystal structure of the homodimeric lectin-like oxLDL-binding domain was used (PDB ID: 7W5D). The three-dimensional structure of 5-HT was retrieved from the DrugBank database (Accession: DB08839).

Prior to docking, water molecules and heteroatoms were removed from the protein structures. The potential binding pockets on CD36 and OLR1 were predicted using the online tool POCASA 1.1. Docking calculations were then carried out using the DOCK 6.9 program as implemented on the online server of Guangzhou Yinfo Information Technology. The docking search was focused on the predicted binding pockets, and default parameters were used for energy scoring and pose optimization. The resulting docking poses were visualized and analyzed using *UCSF Chimera* software. The binding pose with the most favorable Grid Score was selected for each receptor-ligand pair for subsequent analysis.

Antibody array analysis

Primary peritoneal macrophages from wild-type and *Tgm2*$^{-/-}$ mice were stimulated with IL-13 for 6 hours and lysed. Whole-cell lysates were biotinylated using the Phospho Explorer Antibody Array (PEX100, Full Moon BioSystems, Sunnyvale, CA, USA). The processed samples were analyzed by Wayen Biotechnologies (Shanghai, China).

Briefly, array slides were blocked and incubated with ~100 μg of biotinylated protein lysate in coupling solution for 2 hours. After extensive washing, bound proteins were detected with Cy3-conjugated streptavidin. Fluorescence signals were acquired using an Axon GenePix 4000B microarray scanner (Molecular Devices, San Jose, CA, USA), and image quantification was performed with GenePix Pro 6.0 software.

The phosphorylation level of each protein was expressed as the ratio of its phosphorylated signal to the corresponding non-phosphorylated signal. Proteins exhibiting a fold change greater than 20% between wild-type and *Tgm2*$^{-/-}$ macrophages were considered differentially phosphorylated. Functional pathway enrichment analysis of these proteins was performed using the DAVID bioinformatics platform and the Kyoto Encyclopedia of Genes and Genomes (KEGG) database.

ATAC-Sequencing analysis

Primary peritoneal macrophages from *wild-type* and *Tgm2*$^{-/-}$ mice treated with IL-13 were collected, washed with cold PBS, and then lysed in 200 μL ice-cold lysis buffer. Nuclei were resuspended in 50 μL transposition mix, incubated, and purified using AFTMag NGS DNA Clean Beads. Amplified libraries underwent bead purification with the ATAC-Seq Lib Prep Kit for Illumina (ABclonal, RK21509), and were sequenced and analyzed using Illumina NovaSeq 6000.

Coimmunoprecipitation (Co-IP)

Co-IP was conducted using the Co-IP Kit (absin, abs9649) after cell incubation with IL-13 for 6 hours. Immunoprecipitation was done with TGM2 antibody (Santa Cruze, sc-73612, 2 ug). Immunoblot analyses, which included samples of cell lysates representing the co-immunoprecipitation inputs, were performed using antibodies against TAF3 (absin, abs148707, 1:500) and TAF10 (Proteintech, 20346-1-AP, 1:500).

Reference:

1 Fokkens WJ, Lund VJ, Hopkins C, Hellings PW, Kern R, Reitsma S *et al.* European Position Paper on Rhinosinusitis and Nasal Polyps 2020. *Rhinology* 2020; **58**: 1–464.

2 Wang W, Xu Y, Wang L, Zhu Z, Aodeng S, Chen H *et al.* Single-cell profiling identifies mechanisms of inflammatory heterogeneity in chronic rhinosinusitis. *Nat Immunol* 2022; **23**: 1484–1494.

3 Venkatesan P. 2023 GINA report for asthma. *Lancet Respir Med* 2023; **11**: 589.

4 Wise SK, Damask C, Roland LT, Ebert C, Levy JM, Lin S *et al.* International consensus statement on allergy and rhinology: Allergic rhinitis - 2023. *Int Forum Allergy Rhinol* 2023; **13**: 293–859.

Table E1. Clinical characteristics of included human subjects.

	HC (n = 46)	neCRS (n = 37)	eCRS (n = 46)	eCRSwAS (n = 16)
Age, years	43.84±12.90	41.71±15.10	49.46±12.80	55.25±10.27
Female sex, n (%)	20 (43.48%)	9 (24.32%)	10 (21.74%)	7 (43.75%)
Total IgE (kIU/L)	79.47±93.65	82.59±126.00	227.70±534.90	647.50±648.80[abc]
Eosinophils count, x 10^9/L	0.14±0.11	0.17±0.13	0.39±0.27[*#]	0.44±0.30[ab]
Eosinophils percent, %	2.30±1.82	2.70±2.35	6.04±4.31[*#]	7.17±5.30[ab]
Smoking history, n (%)	9 (19.57%)	13 (35.14%)	17 (36.96%)	4 (25.00%)
AR history, n (%)	4 (8.70%)	5 (13.51%)	8 (17.39%)	7 (43.75%)[a]
HDM positive, n (%)	2 (4.35%)	4 (10.81%)	6 (13.04%)	7 (43.75%)[a]
Recurrence, n (%)	/	5 (13.51%)	8 (17.39%)	7 (43.75%)
FEV1 (% predicted)	93.36±14.14	86.31±24.48	87.93±16.34	74.14±29.71[a]
FEV1/FVC (% predicted)	107.65±8.98	106.57±11.814	104.03±17.10	81.09±25.12[abc]
PEF (% predicted)	79.40±18.87	72.22±14.30	70.51±16.96	67.88±21.09
FEF25 (% predicted)	87.22±23.77	80.15±14.08	74.56±24.09	52.97±40.59[ab]
FEF75 (% predicted)	99.22±44.48	88.47±38.72	91.37±51.28	55.50±51.85[a]

[*] eCRS vs. neCRS, $p < 0.05$;

[#] eCRS vs. HC, $p < 0.05$;

[a] eCRSwAS NP vs. HC, $p < 0.05$;

[b] eCRSwAS vs. neCRS, $p < 0.05$;

[c] eCRSwAS vs. eCRS, $p < 0.05$.

Table E2. Cytokines and inhibitors used in this study.

Reagent	Manufacturer	Clone ID	Concentration
Mouse IL-13	Novoprotein, Suzhou, China	CX57	20 ng/mL
Mouse IL-33	Novoprotein, Suzhou, China	CG73	5 ng/mL
Mouse TSLP	Novoprotein, Suzhou, China	CJ69	10 ng/mL
Mouse IL-4	Novoprotein, Suzhou, China	CK74	20 ng/mL
Mouse TGF-β1	Novoprotein, Suzhou, China	CK33	5 ng/mL
Mouse IL-10	Novoprotein, Suzhou, China	C697	10 ng/mL
Mouse IFN-γ	Novoprotein, Suzhou, China	C746	20 ng/mL
Poly (I:C)	MCE, Shanghai, China	HY-107202	20 μg/mL
LPS	Sigma-Aldrich, US	L4391	100 ng/mL
SEB	Sigma-Aldrich, US	324798	1 μg/mL
HDM	GREER, US	XPB91D3A2.5	1.25 μg/uL
Serotonin	MCE, Shanghai, China	HY-B1473A	100 μM
5-Hydroxytryptophan	MCE, Shanghai, China	HY-N0122	10 mg/Kg
DM-α-KG	MCE, Shanghai, China	HY-44134	10 μM
Itaconate	MCE, Shanghai, China	HY-Y0520	100 μM
TGM2 Inhibitor II,	Merck KGaA, Darmstadt,	5.09522	10 μM

ERW1041E	Germany		
TG-2-IN-1	MCE, Shanghai, China	HY-117678	10 μM
N-Phenylacrylamide	Targetmol, US	2210-24-4	10 μM
ZM39923 hydrochloride	Targetmol, US	1021868-92-7	10 μM
A485	MCE, Shanghai, China	HY-107455	1 μM
dCBP-1	MCE, Shanghai, China	HY-134582	1 μM
OICR-9429	MCE, Shanghai, China	HY-16993	1 μM
MM401 TFA	MCE, Shanghai, China	HY-19554A	1 μM
DNase I	MCE, Shanghai, China	HY-108882	0.2mg/ml
Dispase II	Sigma-Aldrich, US	04942078001	2mg/ml
Collagenase, Type I	MCE, Shanghai, China	HY-E70005A	2mg/ml
Dynasore	TargetMol, US	T1848	80 μM
EIPA	TargetMol, US	TQ0157	50 μM
SMS121	TargetMol, US	T207789	20 μM
SOS	TargetMol, US	T13036L	20 μM
BI-0115	TargetMol, US	T9108	5 μM

144

Antibodies	Species	Manufacturer	Clone ID
TGM2	Rabbit	Proteintech, Wuhan, China	15100-1-AP
TGM2	Mouse	Santa Cruze, US	sc-73612
CD163	Mouse	Santa Cruze, US	sc-20066

FN1	Rabbit	Abmart, Shanghai, China	T59537
CD206	Mouse	Santa Cruze, US	sc-58986
CD206	Rabbit	ABclonal, Wuhan, China	A11192
Vimentin	Rabbit	Boster, Wuhan, China	PB0378
F13A1	Rabbit	ABclonal, Wuhan, China	A1461
CC-L24	Rabbit	Proteintech, Wuhan, China	22306-1-AP
IL-10	Mouse	Proteintech, Wuhan, China	60269-1-Ig
IL-10	Mouse	Santa Cruze, US	sc-32815
IGF-1	Mouse	Santa Cruze, US	sc-518040
IGF-1	Rabbit	Proteintech, Wuhan, China	28530-1-AP
ARG1	Rabbit	ABclonal, Wuhan, China	A4923
ARG2	Rabbit	Abmart, Shanghai, China	TB4959S
CHIL3	Mouse	Invitrogen, US	PA5-81356
F4/80	Rabbit	Absin, Shanghai, China	abs137239
CD41	Rabbit	Aifang, Changsha, China	AFRM81216
KLRG1	Rabbit	Proteintech, Wuhan,	84785-4-RR

		China	
GATA3	Rabbit	Aifang, Changsha, China	AF20240
H3	Rabbit	ABclonal, Wuhan, China	A2348
H3Q5Ser	Rabbit	Sigma-Aldrich, US	ABE1791
H3Q5Ser	Rabbit	ABclonal, Wuhan, China	A20210
H3K4Me3	Rabbit	ABclonal, Wuhan, China	A22146
H3K9Ac	Rabbit	ABclonal, Wuhan, China	A21107
H3K27Ac	Rabbit	ABclonal, Wuhan, China	A22264
TAF3	Rabbit	Absin, Shanghai, China	abs148707
TAF10	Rabbit	Proteintech, Wuhan, China	20346-1-AP
GAPDH	Rabbit	Proteintech, Wuhan, China	10494-1-AP
GAPDH	Mouse	ABclonal, Wuhan, China	AC033
PE/Cyanine anti-human CD45	/	Biolegend, San Diego, US	103114
APC anti-human CD14 Antibody	/	Biolegend, San Diego, US	325608

Brilliant Violet 605™ anti-mouse/human CD11b Antibody	/	Biolegend, San Diego, US	101257
CoraLite® Plus 488-conjugated TGM2 Monoclonal antibody	/	Proteintech, Wuhan, China	CL488-68006
Goat Anti-Rabbit IgG(H+L)	Rabbit	ABclonal, Wuhan, China	AS070
Polymer-HRP anti-mouse/rabbit universal secondary antibody IgG	/	Aifang, Changsha, China	AFIHC001

Table E3: Antibodies used in this study.

Table E4. Human primers used in this study.

	Forward (5’ - 3’)	Reverse (5’ - 3’)
TGM2	CAAGGCCCGTTTTCCACTAAG	GAGGCGATACAGGCCGATG
FN1	CGGTGGCTGTCAGTCAAAG	AAACCTCGGCTTCCTCCATAA
IL10	TCAAGGCGCATGTGAACTCC	GATGTCAAACTCACTCATGGCT
CD80	AAACTCGCATCTACTGGCAAA	GGTTCTTGTACTCGGGCCATA
F13A1	CCCAATAACTCTAATGCAGCGG	ACCTTGTTAGTGTCCCATCTCTC
CD163	TTTGTCAACTTGAGTCCCTTC	TCCCGCTACACTTGTTTTCAC

	AC	

Table E5. Mouse primers used in this study.

	Forward (5' - 3')	Reverse (5' - 3')
Tgm2	GACAATGTGGAGGAGGGATCT	CTCTAGGCTGAGACGGTACAG
Fn1	GATGTCCGAACAGCTATTTACCA	CCTTGCGACTTCAGCCACT
Mrc1	CTCTGTTCAGCTATTGGACGC	CGGAATTTCTGGGATTCAGCTTC
F13a1	GAGCAGTCCCGCCCAATAAC	CCCTCTGCGGACAATCAACTTA
Il10	GCTGGACAACATACTGCTAACC	ATTTCCGATAAGGCTTGGCAA
Ccl24	TCTTGCTGCACGTCCTTTATT	GCATCCAGTTTTTGTATGTGCC
Igf1	CTGGACCAGAGACCCTTTGC	GGACGGGGACTTCTGAGTCTT

Table E6. Reagent Kits used in this study.

Reagent kits	Manufacturer	Clone ID
Mouse CCL-24 ELISA Kit	ABclonal, Wuhan, China	PK07180
Mouse IGF-1 ELISA Kit	ABclonal, Wuhan, China	PK00171
Mouse IL-10 ELISA Kit	ABclonal, Wuhan, China	PK00016
Mouse IL-5 ELISA Kit	ABclonal, Wuhan, China	PK00037

Mouse IL-13 ELISA Kit	ABclonal, Wuhan, China	PK00107
General 5-HT ELISA Kit	ABclonal, Wuhan, China	RK09044
CUT&Tag Assay Kit (pAG-Tn5) for Illumina	ABclonal, Wuhan, China	RK20265
Dual DNA Adapter 96Kit for One-step DNA Lib Prep	ABclonal, Wuhan, China	PK20290
ATAC-Seq Lib Prep Kit for Illumina	ABclonal, Wuhan, China	RK21509
PinpoRNA™ 2.0	Pinpoease, Guangdong, China	PIF3000
rProtein A/G Magnetic IP/Co-IP Kit	Absin, Shanghai, China	abs9649

Table E7. Primers for Cut-tag qPCR.

	Forward (5’ - 3’)	Reverse (5’ - 3’)
Mrc1-1	TCCCACCAAACACTCCCTTT	AGCCCCTGTAGCACATCATT
Mrc1-2	TTTTCCTCCTCGTTCCCCTC	CATACGTGTGCCTATGTGCC
Mrc1-3	TACCTGGTTTGTGCAATGCC	AGGGCGGACTTTCTAGCAAT

Supplementary Figures

Figure S1. Single-cell Transcriptomic Characterization of Nasal Polyp Cellular Heterogeneity in eCRS. (**A**) Heatmap analysis of top differentially expressed marker genes (rows) across identified cell clusters (columns), with z-score normalized expression highlighting cell-type-specific signatures. (**B**) Feature plots showing indicated markers across different cell types.

Figure S2. Monocytes Heterogeneity Distinguished eCRS With And Without Asthma. (**A**) Manhattan volcano plot of differentially expressed genes (DEGs) across all cell clusters comparing eCRS versus eCRSwAS patients (FDR < 0.01, |log2FC| > 0). (**B**) Violin plots quantifying expression levels of classical and alternative

macrophage activation markers in monocytes, stratified by disease states (eCRS vs eCRSwAS). (**C**) Enriched Gene Set Enrichment Analysis (GSEA) pathways (FDR < 0.05) from DEGs between monocytes from different disease states, ranked by normalized enrichment score (NES). (**D**) UMAP visualization of 13,387 monocytes from nasal polyp samples, colored by 12 transcriptionally distinct subclusters and stratified by disease state (left: eCRS; right: eCRSwAS).

Figure S3. Pro-fibrotic Activity of AAMs Aassociated with eCRS Disease Severity. (**A, B**) GO analysis revealed enriched biological processes associated with pro-fibrotic activities in *FN1*^{hi} (**A**) and *TGM2*^{hi} (**B**) macrophages subsets. (**C**) Pseudo-time analysis showed that *TGM2*^{hi} and *FN1*^{hi} macrophages follow a unified differentiation trajectory toward fibrosis-promoting states. (**D**) Locuszoom plots showing the association between *TGM2* variants rs200331470 and rs11696730 with CRSwNP or asthma, respectively. (**E**) Feature plots showing several other macrophage subset markers (*MRC1*, *TREM2* etc.) across monocytes subpopulations on UMAP plot, with blue circles highlighting the most prominent *TGM2*^{hi} macrophage population.

Figure S4. Single-cell Transcriptomic Atlas Reveals Disease-Specific Alternative Macrophage Activation Across CRS Subtypes. (**A**) UMAP visualization of single-cell clusters integrating our local datasets with a public scRNA-seq dataset (HRA000772) from different CRS subtypes, showing disease-specific cellular distributions. (**B**) Dot plot quantifying expression levels (dot size) and prevalence (color intensity) of canonical marker genes for identified cell types. (**C**) Feature plots demonstrating expression patterns of alternative macrophage activation markers (*TGM2*, *FN1*, *MRC1*, *ALOX15 etc.*) across the UMAP embedding. (**D**) Violin plots quantifying disease-specific expression differences of AAM markers (*TGM2*, *FN1*, *MRC1*, *F13A1*, *IGF1*, *IL10*) in macrophage clusters, with kernel density estimation and median values indicated.

Figure S5. TGM2 Is Upregulated in eCRS and Correlates with AAM Markers and Disease Severity. (**A**) Western blot showed TGM2 protein expression in

representative samples from nasal mucosa from healthy controls (HC, n=25) and nasal polyp tissues from neCRS (n=13), eCRS (n=10), and eCRSwAS (n=8) patients. (**B**) Representative images of flow cytometry revealed a significantly expanded TGM2^{+} pro-fibrotic AAM population in eosinophil-rich samples relative to other groups. (**C**, **D**) *TGM2* mRNA positively correlated with *IL10* mRNA expression levels (**C**; n=30), while showing no significant correlation with the type 1 inflammatory marker *CD80* (**D**; n=28). (**E, F**) Clinically, TGM2 protein expression negatively correlating with lung function parameters including forced expiratory flow at 25%-75% (**E**; n=26), while showing no significant correlation with FEV1/FVC (**F**; n=26). Data were presented as mean ± SEM. $*p < 0.05$, $**p < 0.01$, $***p < 0.001$, $****p < 0.0001$; ns, not significant (one-way ANOVA with Dunnett's test for difference analysis, Spearman's correlation analysis for correlation analysis).

Figure S6. *Tgm2* Deficiency Alters Tissue Pathology and Macrophage Landscapes in Dust Mite-Induced Inflammation. (**A**) Quantitative RT-PCR analysis of *Tgm2* mRNA levels in nasal mucosa and lung tissues from dust mite-challenged wild-type mice versus unchallenged controls (n=6/group). (**B, C**) Representative histochemical staining and quantification analysis revealing elevated infiltration of TGM2^{+} macrophages in nasal mucosa of dust mite treated wild-type mice. Scale bar: 100μm. (**D-G**) Representative histochemical staining (Giemsa for granulocytes, Sirius Red for collagen) demonstrating reduced eosinophil infiltration (**D** for nasal mucosa, **E** for lung tissues) and collagen deposition (**F** for nasal mucosa, **G** for lung tissues) in dust mite-exposed *Tgm2*$^{-/-}$ mice versus wild-type controls. (**H**) UMAP projection of 64,662 single cells from nasal mucosa from dust mite-challenged wild-type and *Tgm2*$^{-/-}$ mice, colored by 10 transcriptionally defined cell types. (**I**) Grouped bar plot quantifying proportional changes in major cell populations between genotypes, highlighting a substantial reduction in macrophage subsets in *Tgm2*$^{-/-}$ mice. (**J**) *t*-SNE plot of 21,808 macrophages from nasal mucosa of dust mite-treated wild-type and *Tgm2*$^{-/-}$ mice, colored by 10 distinct macrophage clusters, defined by their signature gene which expression levels significantly higher than the population

average. (**K**) Feature plots visualizing the expression of the other AAM markers across myeloid cells in *t*-SNE space, supplemented to **Figure 3U**. Data: mean ± SEM; *p<0.05, **p<0.01, ***p<0.001.

Figure S7. *Tgm2* Governs Alternative Macrophage Effector Functions and Tissue Remodeling in Allergic Inflammation. (**A, B**) Quantification of Western blot analysis confirms reduced protein expression of canonical AAM markers (CD206, FN1, F13A1, IL-10, ARG1, ARG2) in both nasal mucosa and lung tissues of dust mite-challenged *Tgm2*$^{-/-}$ mice compared to wild-type controls, supplemented to **Figure 4C**. (**C**) Measurement of IL-10, IGF-1, CCL-24 concentrations in NLF from wild-type and *Tgm2*$^{-/-}$ mice after dust mite challenge. Data: mean ± SEM; *p<0.05, **p<0.01, ***p<0.001.

Figure S8. *Tgm2* Is Selectively Induced by Type 2 Inflammatory Cytokines in Macrophages. (**A**) Western blot analysis demonstrated TGM2 protein expression dynamics in primary peritoneal macrophages stimulated for indicated time points within 24 hours with different PAMPs and cytokines, including LPS (100 ng/mL), Poly (I:C) (20 μg/mL), SEB (1 μg/mL), LPS (100 ng/mL) + IFN-γ (20 ng/mL), IL-4 (20 ng/mL), IL-13 (20 ng/mL), TGF-β1 (5 ng/mL), IL-10 (20 ng/mL), DM-α-KG (10 μM), IL-33 (50 ng/mL), TSLP (50 ng/mL), and Itaconate (100 μM). (**B**) Quantification revealed significant TGM2 induction specifically by type 2 cytokines compared to unstimulated controls. Data: mean ± SEM; *p<0.05, **p<0.01, ***p<0.001.

Figure S9. *Tgm2* Integrates Extracellular Matrix Remodeling and Immune Signaling During Alternative Macrophage Activation. (**A**-**C**)Heatmap analysis demonstrated significantly altered expression of genes associated with macrophage lineage identity, classical and alternative macrophage activation (**A**), extracellular matrix organization (**B**), and complement cascade and MHC-II complex (**C**) in IL-13-treated *Tgm2*$^{-/-}$ macrophages compared to wild-type controls, revealing broad impairment of type 2 response pathways. (**D-F**) Functional enrichment analysis of DEGs showed that *Tgm2* deficiency leads to downregulation of type 2 immune

response pathways and extracellular matrix organization, as evidenced by GSEA pathway (**D**), and biological processes (**E**: upregulated; **F**: downregulated).

Figure S10. *Tgm2* Serves as an Integrative Signaling Hub Critical for Alternative Macrophage Activation. (**A**-**C**) Quantification of western blot analysis revealed significant reduced protein expression of TGM2, CD206, FN1 and CCL-24 in *Tgm2*$^{-/-}$ macrophages treated with IL-13 (**A**), IL-33 (**B**) and TSLP (**C**), supplemented to **Figure 5D**. (**D**) Immunofluorescence staining confirmed the selective deletion of *Tgm2* in CD206^{+} macrophages. (**E-I**) HE and Giemsa images of nasal mucosa (**E, F** for HE; and **H** for Giemsa) and lung (**G** for HE; and **I** for Giemsa) tissues from dust mite-exposed wild-type versus macrophage-specific *Tgm2* knockout (*Tgm2-mKO*) mice, showing attenuated pathology in knockouts. **E, G**: red arrowhead indicates prominent inflammatory foci. **F**: black I-bars indicates the epithelial thickness. **H, I**: yellow arrowhead indicates the eosinophils infiltration. Scale bar: 100μm. Data: mean ± SEM; *p<0.05, **p<0.01, ***p<0.001.

Figure S11. *Tgm2* Deficiency Minimally Affects Intracellular Signaling Pathways During Alternative Macrophage Activation. (**A**) Volcano plot analysis of phospho-protien array data revealed no significant differences (FDR < 0.05, |log2FC| > 1) in phosphorylation levels of 160 well-characterized signaling proteins between IL-13-treated (20 ng/mL, 4 hours) wild-type and *Tgm2*$^{-/-}$ macrophages, suggesting *Tgm2* regulates AAM polarization through mechanisms largely independent of canonical intracellular signaling pathway activation.

Figure S12. *Tgm2* Is Required for H3Q5Ser Induced by T2I Factors. (**A**, **B**) Quantification of western blot analysis revealed significant reduced activation levels of H3Q5Ser and H3K4Me3 in *Tgm2*$^{-/-}$ macrophages treated with IL-13, IL-33 and TSLP, supplemented to **Figure 6A**. (**C**, **D**) Multicolor immunofluorescence staining and quantification analysis demonstrated co-localization of H3Q5Ser with CD206 in nasal mucosa from dust mite-treated wild-type versus *Tgm2*$^{-/-}$ mice, supplemented to **Figure 6B**. (**E**) Western blotting of H3Q5Ser levels in nasal mucosa samples from HC, neCRS, eCRS and eCRSwAS. (**F, G**) Multicolor immunofluorescence staining

and quantification analysis confirmed the elevated H3Q5Ser levels in macrophages from eCRS and eCRSwAS samples, supplemented to **Figure 6E**. (**H**) Quantificative analysis of CD206, TGM2 and CCL-24 expression in wild-type and *Tgm2*$^{-/-}$ macrophages stimulated with 5-HT (0.1 mM) at indicated time points within 24 hours (by multiple unpaired *t*-test with Benjamini-Hochberg (FDR) correction), supplemented to **Figure 6N**. Data: mean ± SEM; *$p<0.05$, **$p<0.01$, ***$p<0.001$.

Figure S13. Exogenous Serotonin Exacerbates Type 2 Inflammation. (**A-D**) Representative histochemical staining revealed reduced eosinophil infiltration (**A** for nasal mucosa, **B** for lung tissues) and collagen deposition (**C** for nasal mucosa, **D** for lung tissues) from 5-HTP and dust mite co-treated mice, along with the groups with 5-HTP and dust mite alone. Scale bar: 100μm. (**E**) Measurement of 5-HT concentrations in NLF, BLF and serum from 5-HTP and dust mite co-treated mice, along with the groups with 5-HTP and dust mite alone. (**F**) ELISA quantification revealed no significant alterations of IL-5 in NLF from mice co-exposed to dust mite and 5-HTP. (**G**) Western blot quantitative analysis showed amplified expression of AAM markers (CD206, TGM2, F13A1, IL-10) in nasal mucosa from 5-HTP+dust mite co-treated mice, supplemented to **Figure 6V**. (**H**, **I**) qRT-PCR analysis showed the expression levels of AAM and profibrotic markers (*Tgm2*, *Il10*, *Fn1*, *F13a1*) in nasal mucosa and lung tissues from 5-HTP+dust mite co-treated mice. Data: mean ± SEM; *$p<0.05$, **$p<0.01$, ***$p<0.001$.

Figure S14. Supplementary Evidence for that 5-HT Aggravates Type 2 Inflammation. (A) Quantitative analysis revealed the expansion of GATA3^{+}KLRG1^{+} cells in nasal polyp tissues from eCRSwAS patients than eCRS samples, especially in FN1-abundant areas. (**B**, **C**) Representative Sirius red staining of (**B**) nasal mucosa and (**C**) lung tissues from house dust mite-challenged wild-type and *Tph1*$^{-/-}$ mice, showing attenuated collagen deposition in the absence of peripheral 5-HT. Scale bars: 100 μm. (**D**) Measurement of 5-HT concentrations in NLF, BLF and serum from in dust mite-exposed *Tph1*$^{-/-}$ mice versus wild-type controls.

Figure S15. Supplementary Evidence for the Serotonergic Regulation of Macrophage Activation. (**A**, **B**) Violin plots showing the expression levels of canonical serotonin transporters and receptors across major cell types in (**A**) human nasal polyps and (**B**) dust mite -challenged mouse nasal mucosa, as determined by scRNA-seq. Macrophages or Monocytes notably lack expression of these canonical genes. (**C**, **D**) Molecular docking models predicting the binding of 5-HT (shown as sticks) to (**C**) CD36 and (**D**) OLR1. For each receptor, the panels show the overall protein structure (ribbon), the molecular surface of the binding pocket, and a detailed view of the interacting residues.

Figure S16. TGM2-mediated H3Q5Ser facilitates AAM transcription through the H3K4Me3-TFIID axis. (**A**) Metagene profiles (top) and corresponding heatmaps (bottom) of H3Q5Ser and active histone marks (H3K4Me3, H3K9Ac, H3K27Ac) surrounding transcriptional start sites (TSS, ± 3 kb) in macrophages, demonstrating IL-13-induced (20 ng/mL, 24 hours) genome-wide redistribution of these epigenetic modifications. (**B**) Differential peak analysis revealed localized increases in H3K4Me3 signal at key AAM-associated loci such as *Mrc1* and *Arg1*. (**C**-**F**) Volcano plots illustrating genes with statistically significant differential enrichment in H3Q5Ser, H3K4Me3, H3K9Ac, and H3K27Ac binding upon IL-13 stimulation. (**G**) Parallel analysis of TGM2 binding patterns revealed IL-13-dependent recruitment to promoter-proximal regions. (**H**) Genome browser tracks of TGM2 occupancy at canonical AAM-associated loci (*Fn1*, *Mrc1*, *Il10*, *F13a1*). (**I**) ATAC-sequencing analysis revealed no significant alterations in global chromatin accessibility upon *Tgm2* knockout. (**J**) CUT&Tag-qPCR analysis showed that *Tgm2*$^{-/-}$ macrophages exhibited markedly reduced H3Q5Ser, H3K4Me3, H3K9Ac, and H3K27Ac occupancy at the *Mrc1* promoter. (**K**) Co-expression of *TGM2* with the TFIID subunit *TAF10*, but not *TAF3*, in macrophages from human eCRS nasal polyps (left) and dust mite challenged mouse nasal mucosa (right), as shown in single-cell RNA-sequencing datasets. (**L**) Quantitative analysis of western blots of AAM gene expression (CD206, ARG1, IGF-1) in macrophages treated with 5-HT in the presence or absence of

epigenetic inhibitors. Cells were co-treated with 5-HT and the H3K4 methyltransferase complex inhibitors OICR-9429 (OICR, 1 μM) or MM-401 (1 μM), or the CBP/EP300 inhibitors A485 (1 μM) or dCBP-1 (1 μM).

Figure S17. Pharmacologic Inhibition of the 5-HT-TGM2-H3Q5Ser Axis Attenuates Type 2 Airway Inflammation. (**A, B**) Representative Sirus red staining revealed the remodeling in nasal mucosa (**A**) and lung (**B**) tissues, indicating the comparative evaluation of the therapeutic efficacy of (-)-epicatechin (Epi, 1 mg/kg i.n.), sarpogrelate (Sar, 5 mg/kg i.n.) and rilapladib (Ril, 0.2 mg/kg i.n.) in dust mite-challenged wild-type and *Tgm2*$^{-/-}$ mice. Scale bar: 100μm. (**C, D**) Immunofluorescence staining and quantification analysis confirmed Sar (a selective 5-HT2A receptor antagonist) and Ril (a PAFR inhibitor) attenuated H3Q5Ser levels of macrophages in dust mite induced-T2I mouse model. Scale bar: 100μm. (**E, F**) Representative Sirus red staining revealed the remodeling in nasal mucosa (**E**) and lung (**F**) tissues, indicating the therapeutic efficacy of ERW1041E (10 mg/kg/day i.n.) in dust mite-challenged wild-type and *Tgm2*$^{-/-}$ mice. Scale bar: 100μm.

Figure S18. (Diagram) Platelet-Derived 5-HT Epigenetically Primes Profibrotic Macrophages to Orchestrate Type 2 Airway Inflammation.

Suppl. Fig. S1

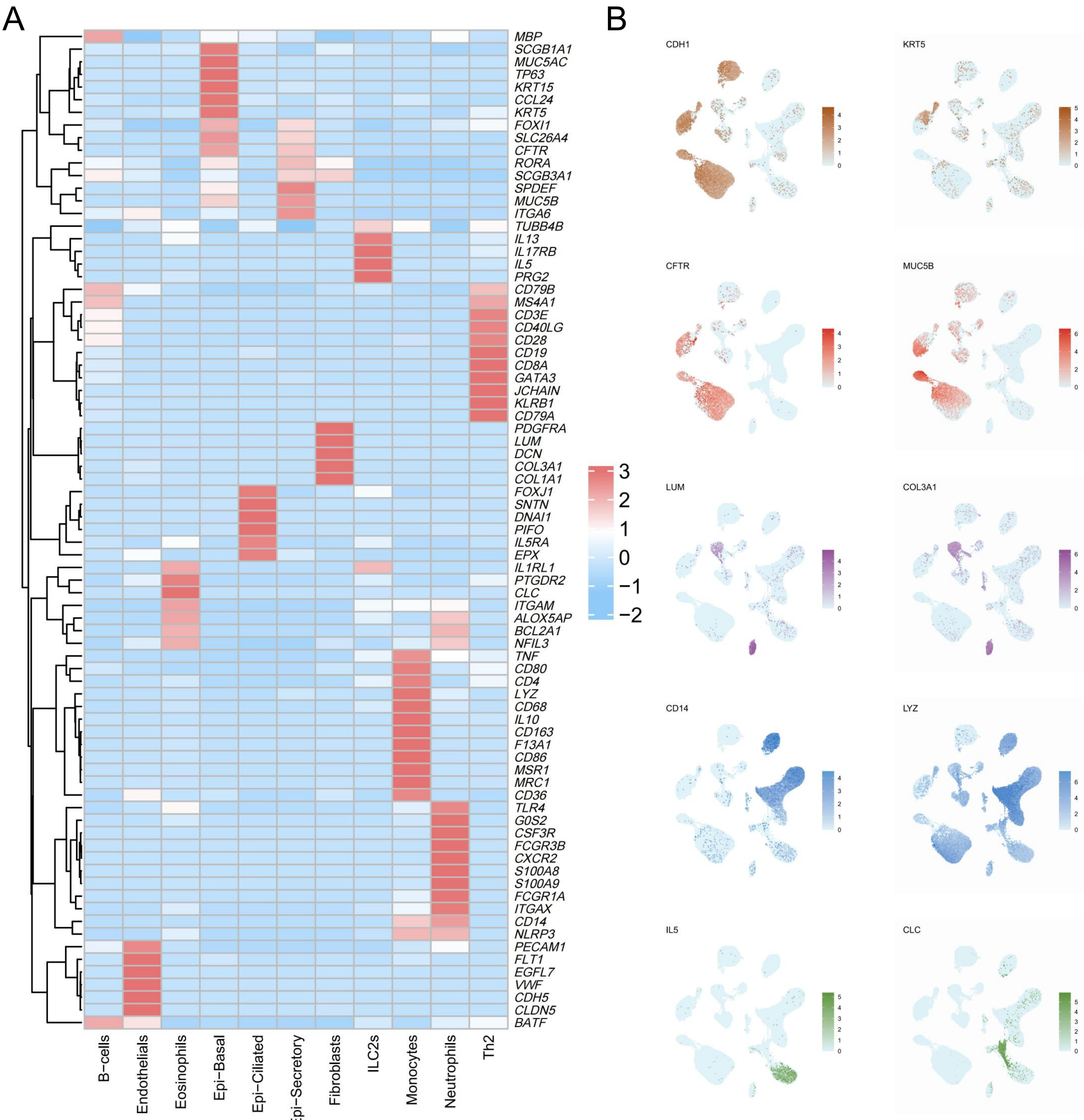
A
B
MBP
SCGB1A1
MUC5AC
TP63
KRT15
CCL24
KRT5
FOXI1
SLC26A4
CFTR
RORA
SCGB3A1
SPDEF
MUC5B
ITGA6
TUBB4B
IL13
IL17RB
IL5
PRG2
CD79B
MS4A1
CD3E
CD40LG
CD28
CD19
CD8A
GATA3
JCHAIN
KLRB1
CD79A
PDGFRA
LUM
DCN
COL3A1
COL1A1
FOXJ1
SNTN
DNAI1
PIFO
IL5RA
EPX
IL1RL1
PTGDR2
CLC
ITGAM
ALOX5AP
BCL2A1
NFIL3
TNF
CD80
CD4
LYZ
CD68
IL10
CD163
F13A1
CD86
MSR1
MRC1
CD36
TLR4
G0S2
CSF3R
FCGR3B
CXCR2
S100A8
S100A9
FCGR1A
ITGAX
CD14
NLRP3
PECAM1
FLT1
EGFL7
VWF
CDH5
CLDN5
BATF
B−cells
Endothelials
Eosinophils
Epi−Basal
Epi−Ciliated
Epi−Secretory
Fibroblasts
ILC2s
Monocytes
Neutrophils
Th2
3
2
1
0
−1
−2
CDH1
KRT5
CFTR
MUC5B
LUM
COL3A1
CD14
LYZ
IL5
CLC

Suppl. Fig. S2

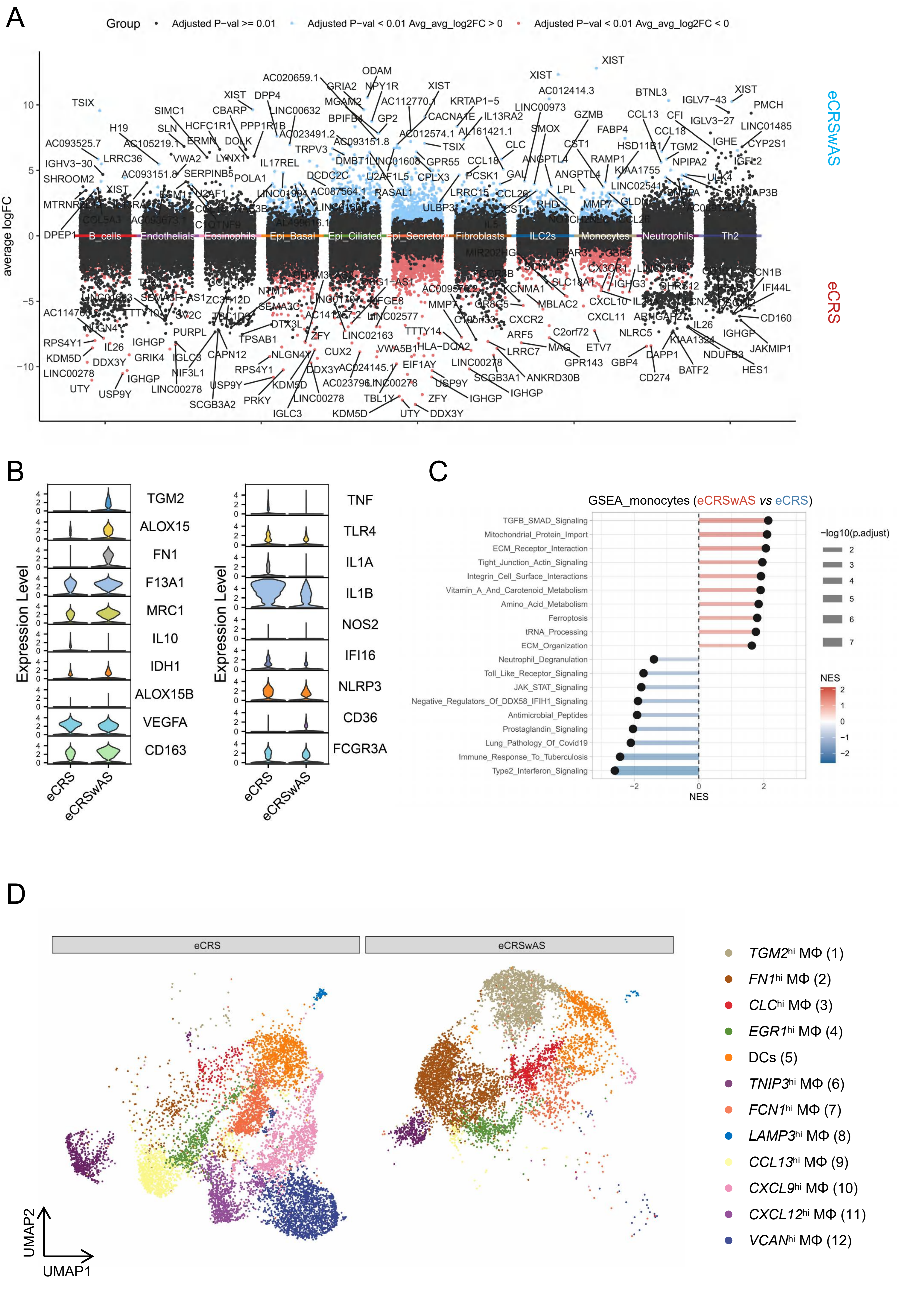

A
Group
Adjusted P−val >= 0.01
Adjusted P−val < 0.01 Avg_avg_log2FC > 0
Adjusted P−val < 0.01 Avg_avg_log2FC < 0
average logFC
eCRSwAS
eCRS
B_cells
Endothelials
Eosinophils
Epi_Basal
Epi_Ciliated
Epi_Secretory
Fibroblasts
ILC2s
Monocytes
Neutrophils
Th2
B
Expression Level
TGM2
ALOX15
FN1
F13A1
MRC1
IL10
IDH1
ALOX15B
VEGFA
CD163
TNF
TLR4
IL1A
IL1B
NOS2
IFI16
NLRP3
CD36
FCGR3A
eCRS
eCRSwAS
C
GSEA_monocytes (eCRSwAS vs eCRS)
TGFB_SMAD_Signaling
Mitochondrial_Protein_Import
ECM_Receptor_Interaction
Tight_Junction_Actin_Signaling
Integrin_Cell_Surface_Interactions
Vitamin_A_And_Carotenoid_Metabolism
Amino_Acid_Metabolism
Ferroptosis
tRNA_Processing
ECM_Organization
Neutrophil_Degranulation
Toll_Like_Receptor_Signaling
JAK_STAT_Signaling
Negative_Regulators_Of_DDX58_IFIH1_Signaling
Antimicrobial_Peptides
Prostaglandin_Signaling
Lung_Pathology_Of_Covid19
Immune_Response_To_Tuberculosis
Type2_Interferon_Signaling
NES
−log10(p.adjust)
D
eCRS
eCRSwAS
UMAP1
UMAP2
TGM2hi MΦ (1)
FN1hi MΦ (2)
CLChi MΦ (3)
EGR1hi MΦ (4)
DCs (5)
TNIP3hi MΦ (6)
FCN1hi MΦ (7)
LAMP3hi MΦ (8)
CCL13hi MΦ (9)
CXCL9hi MΦ (10)
CXCL12hi MΦ (11)
VCANhi MΦ (12)

Suppl. Fig. S3

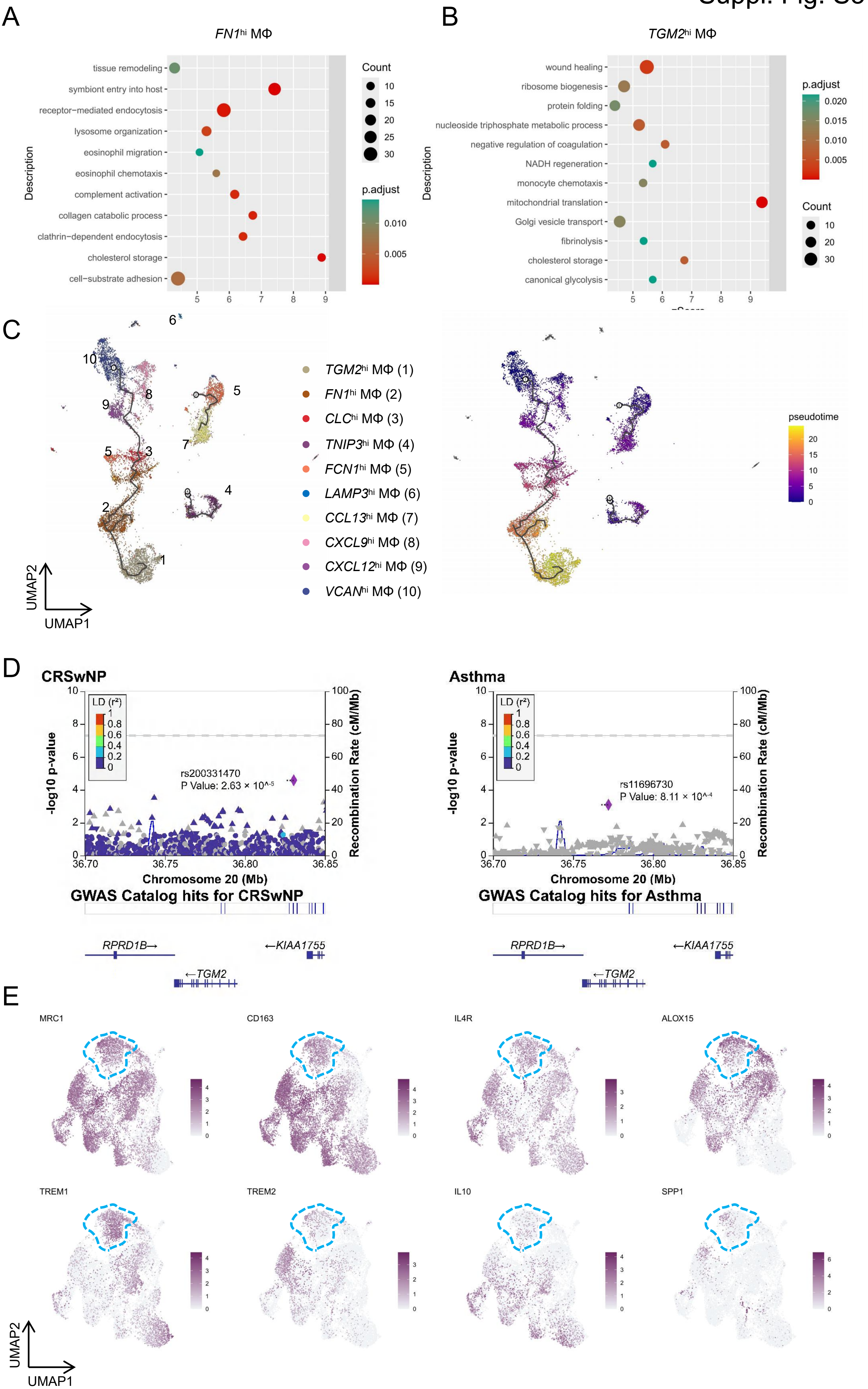

A
FN1hi MΦ
tissue remodeling
symbiont entry into host
receptor−mediated endocytosis
lysosome organization
eosinophil migration
eosinophil chemotaxis
complement activation
collagen catabolic process
clathrin−dependent endocytosis
cholesterol storage
cell−substrate adhesion
Description
Count
10
15
20
25
30
p.adjust
0.010
0.005
5
6
7
8
9
B
TGM2hi MΦ
wound healing
ribosome biogenesis
protein folding
nucleoside triphosphate metabolic process
negative regulation of coagulation
NADH regeneration
monocyte chemotaxis
mitochondrial translation
Golgi vesicle transport
fibrinolysis
cholesterol storage
canonical glycolysis
Description
p.adjust
0.020
0.015
0.010
0.005
Count
10
20
30
5
6
7
8
9
C
TGM2hi MΦ (1)
FN1hi MΦ (2)
CLChi MΦ (3)
TNIP3hi MΦ (4)
FCN1hi MΦ (5)
LAMP3hi MΦ (6)
CCL13hi MΦ (7)
CXCL9hi MΦ (8)
CXCL12hi MΦ (9)
VCANhi MΦ (10)
UMAP2
UMAP1
pseudotime
20
15
10
5
0
D
CRSwNP
LD (r²)
1
0.8
0.6
0.4
0.2
0
rs200331470
P Value: 2.63 × 10^-5
-log10 p-value
Recombination Rate (cM/Mb)
Chromosome 20 (Mb)
36.70
36.75
36.80
36.85
GWAS Catalog hits for CRSwNP
RPRD1B→
←KIAA1755
←TGM2
Asthma
rs11696730
P Value: 8.11 × 10^-4
GWAS Catalog hits for Asthma
E
MRC1
CD163
IL4R
ALOX15
TREM1
TREM2
IL10
SPP1
UMAP2
UMAP1

Suppl. Fig. S4

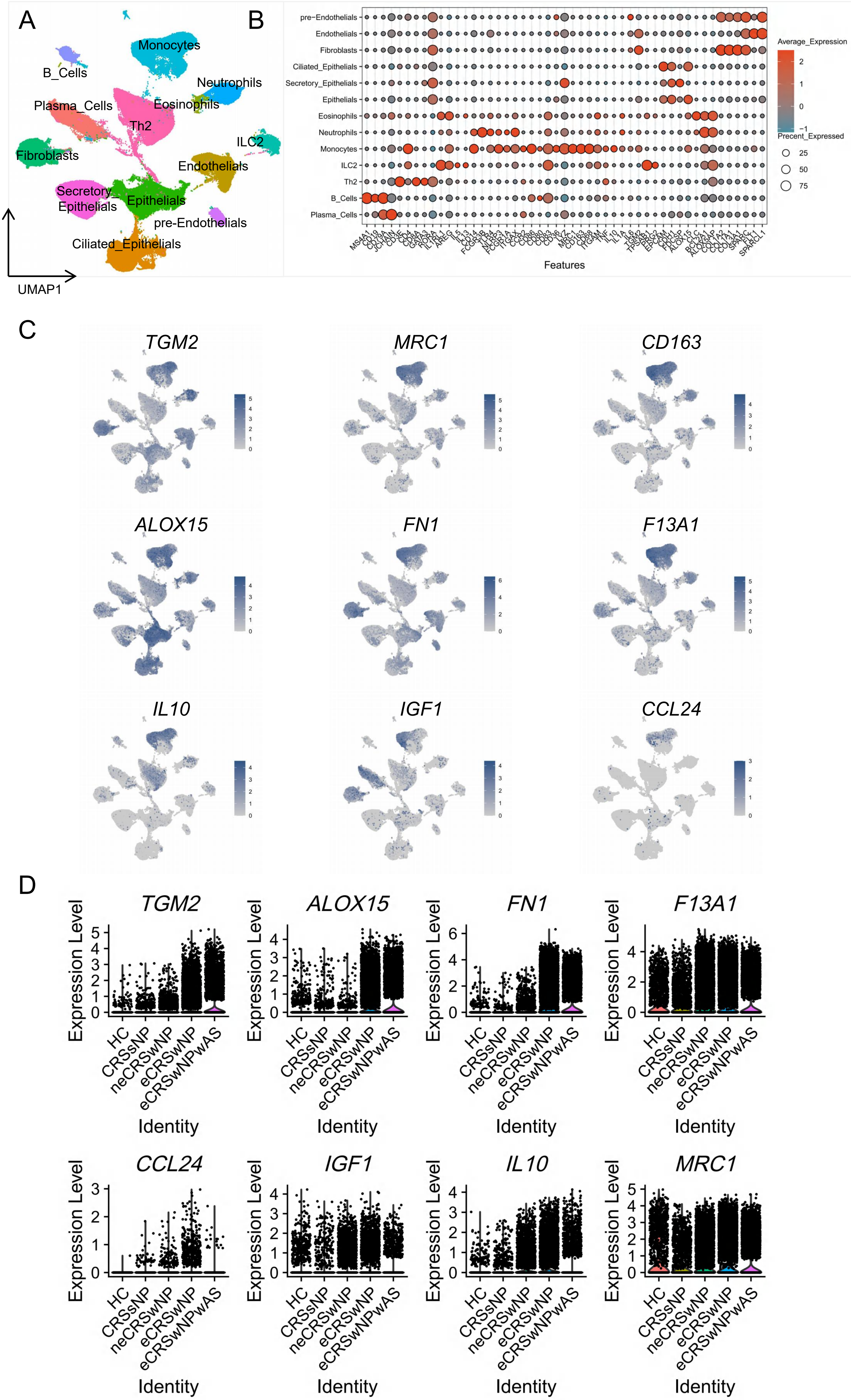
A
Monocytes
B_Cells
Neutrophils
Plasma_Cells
Eosinophils
Th2
ILC2
Fibroblasts
Endothelials
Secretory_Epithelials
Epithelials
pre-Endothelials
Ciliated_Epithelials
UMAP2
UMAP1
B
pre−Endothelials
Endothelials
Fibroblasts
Ciliated_Epithelials
Secretory_Epithelials
Epithelials
Eosinophils
Neutrophils
Monocytes
ILC2
Th2
B_Cells
Plasma_Cells
Features
Average_Expression
Precent_Expressed
25
50
75
C
TGM2
MRC1
CD163
ALOX15
FN1
F13A1
IL10
IGF1
CCL24
D
TGM2
ALOX15
FN1
F13A1
CCL24
IGF1
IL10
MRC1
Expression Level
HC
CRSsNP
neCRSwNP
eCRSwNP
eCRSwNPwAS
Identity

Suppl. Fig. S5

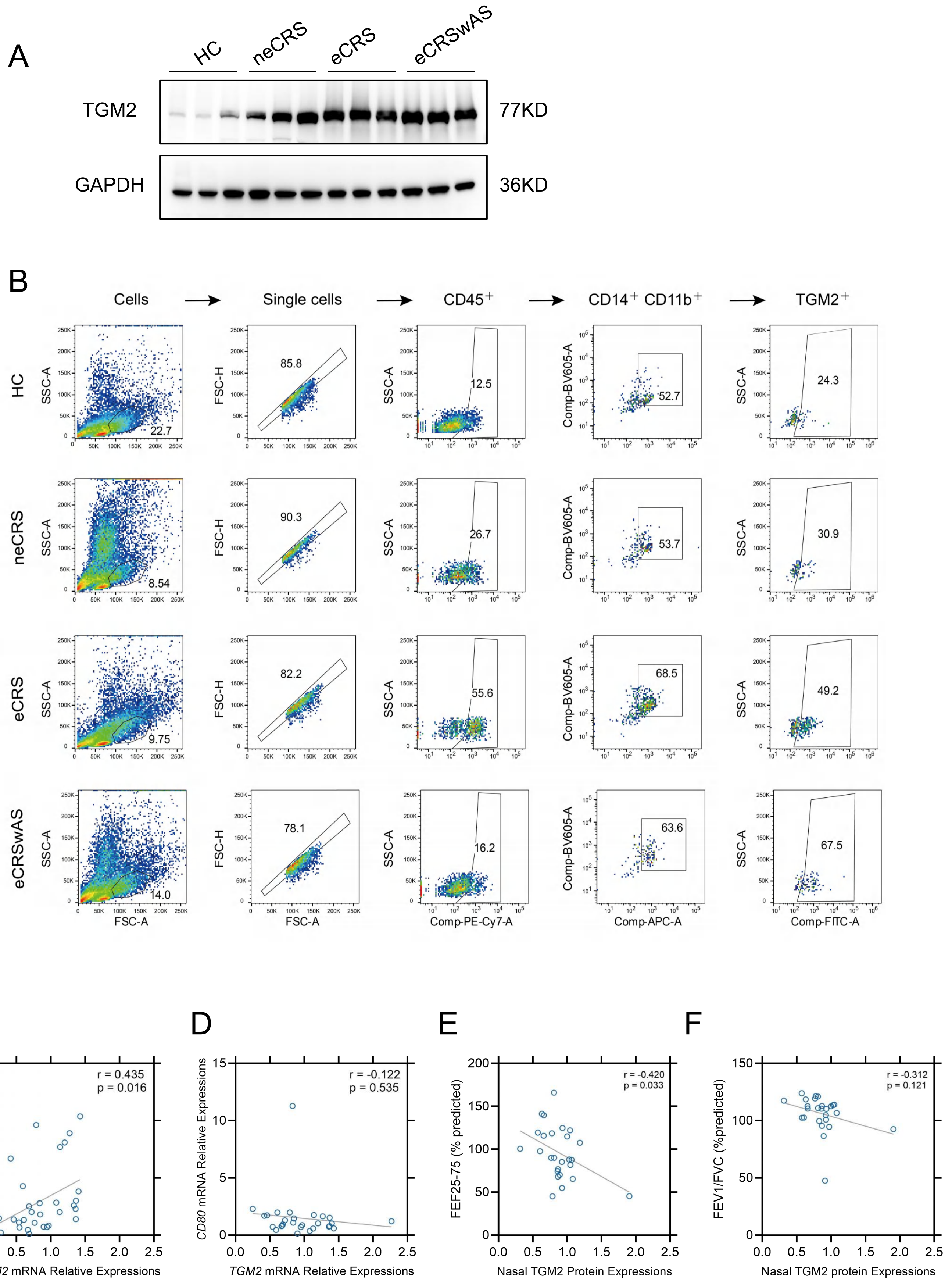
A
HC
neCRS
eCRS
eCRSwAS
TGM2
77KD
GAPDH
36KD
B
Cells
Single cells
CD45+
CD14+ CD11b+
TGM2+
HC
neCRS
eCRS
eCRSwAS
SSC-A
FSC-H
Comp-BV605-A
FSC-A
Comp-PE-Cy7-A
Comp-APC-A
Comp-FITC-A
22.7
85.8
12.5
52.7
24.3
8.54
90.3
26.7
53.7
30.9
9.75
82.2
55.6
68.5
49.2
14.0
78.1
16.2
63.6
67.5
C
r = 0.435
p = 0.016
IL10 mRNA Relative Expressions
TGM2 mRNA Relative Expressions
D
r = -0.122
p = 0.535
CD80 mRNA Relative Expressions
TGM2 mRNA Relative Expressions
E
r = -0.420
p = 0.033
FEF25-75 (% predicted)
Nasal TGM2 Protein Expressions
F
r = -0.312
p = 0.121
FEV1/FVC (%predicted)
Nasal TGM2 protein Expressions

Suppl. Fig. S6

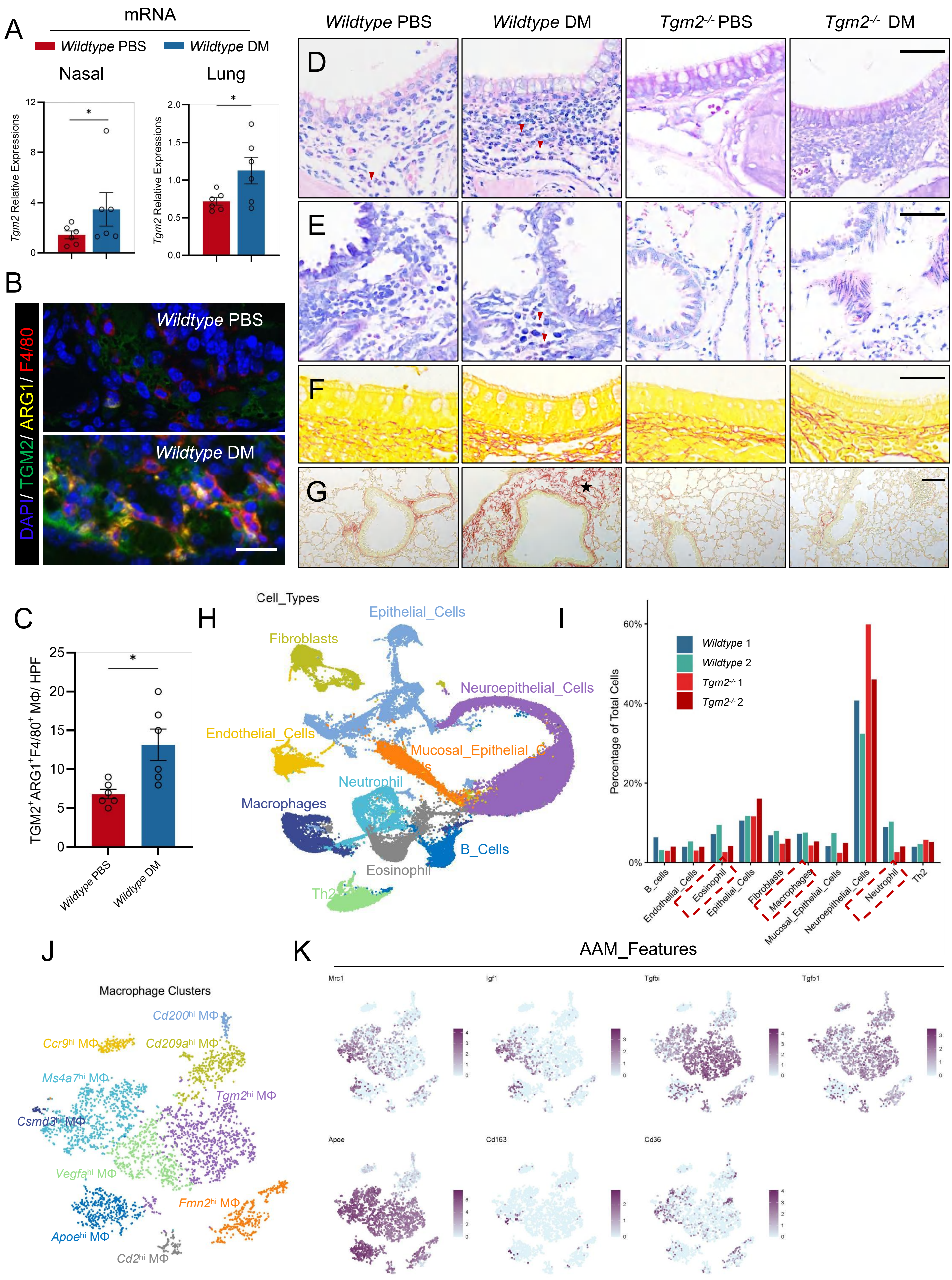
A
mRNA
Wildtype PBS
Wildtype DM
Nasal
Lung
Tgm2 Relative Expressions
B
DAPI/ TGM2/ ARG1/ F4/80
Wildtype PBS
Wildtype DM
C
TGM2+ARG1+F4/80+ MΦ/ HPF
Wildtype PBS
Wildtype DM
Wildtype PBS
Wildtype DM
Tgm2-/- PBS
Tgm2-/- DM
D
E
F
G
H
Cell_Types
Epithelial_Cells
Fibroblasts
Neuroepithelial_Cells
Endothelial_Cells
Mucosal_Epithelial_Cells
Neutrophil
Macrophages
B_Cells
Eosinophil
Th2
I
Wildtype 1
Wildtype 2
Tgm2-/- 1
Tgm2-/- 2
Percentage of Total Cells
B_cells
Endothelial_Cells
Eosinophil
Epithelial_Cells
Fibroblasts
Macrophages
Mucosal_Epithelial_Cells
Neuroepithelial_Cells
Neutrophil
Th2
J
Macrophage Clusters
Cd200hi MΦ
Ccr9hi MΦ
Cd209ahi MΦ
Ms4a7hi MΦ
Tgm2hi MΦ
Csmd3hi MΦ
Vegfahi MΦ
Fmn2hi MΦ
Apoehi MΦ
Cd2hi MΦ
K
AAM_Features
Mrc1
Igf1
Tgfbi
Tgfb1
Apoe
Cd163
Cd36

Suppl. Fig. S7

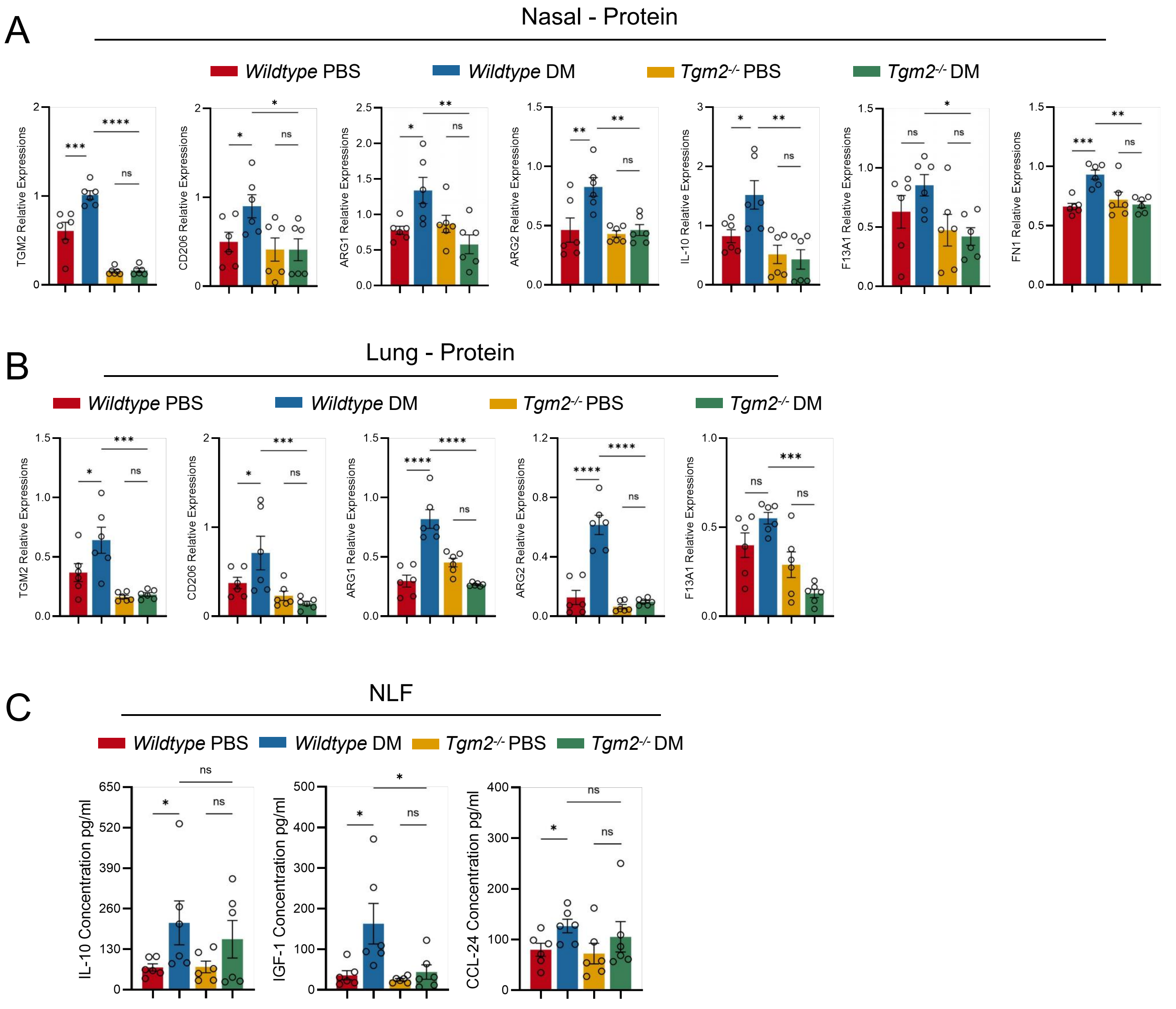
A
Nasal - Protein
Wildtype PBS
Wildtype DM
Tgm2-/- PBS
Tgm2-/- DM
TGM2 Relative Expressions
CD206 Relative Expressions
ARG1 Relative Expressions
ARG2 Relative Expressions
IL-10 Relative Expressions
F13A1 Relative Expressions
FN1 Relative Expressions
B
Lung - Protein
Wildtype PBS
Wildtype DM
Tgm2-/- PBS
Tgm2-/- DM
TGM2 Relative Expressions
CD206 Relative Expressions
ARG1 Relative Expressions
ARG2 Relative Expressions
F13A1 Relative Expressions
C
NLF
Wildtype PBS
Wildtype DM
Tgm2-/- PBS
Tgm2-/- DM
IL-10 Concentration pg/ml
IGF-1 Concentration pg/ml
CCL-24 Concentration pg/ml

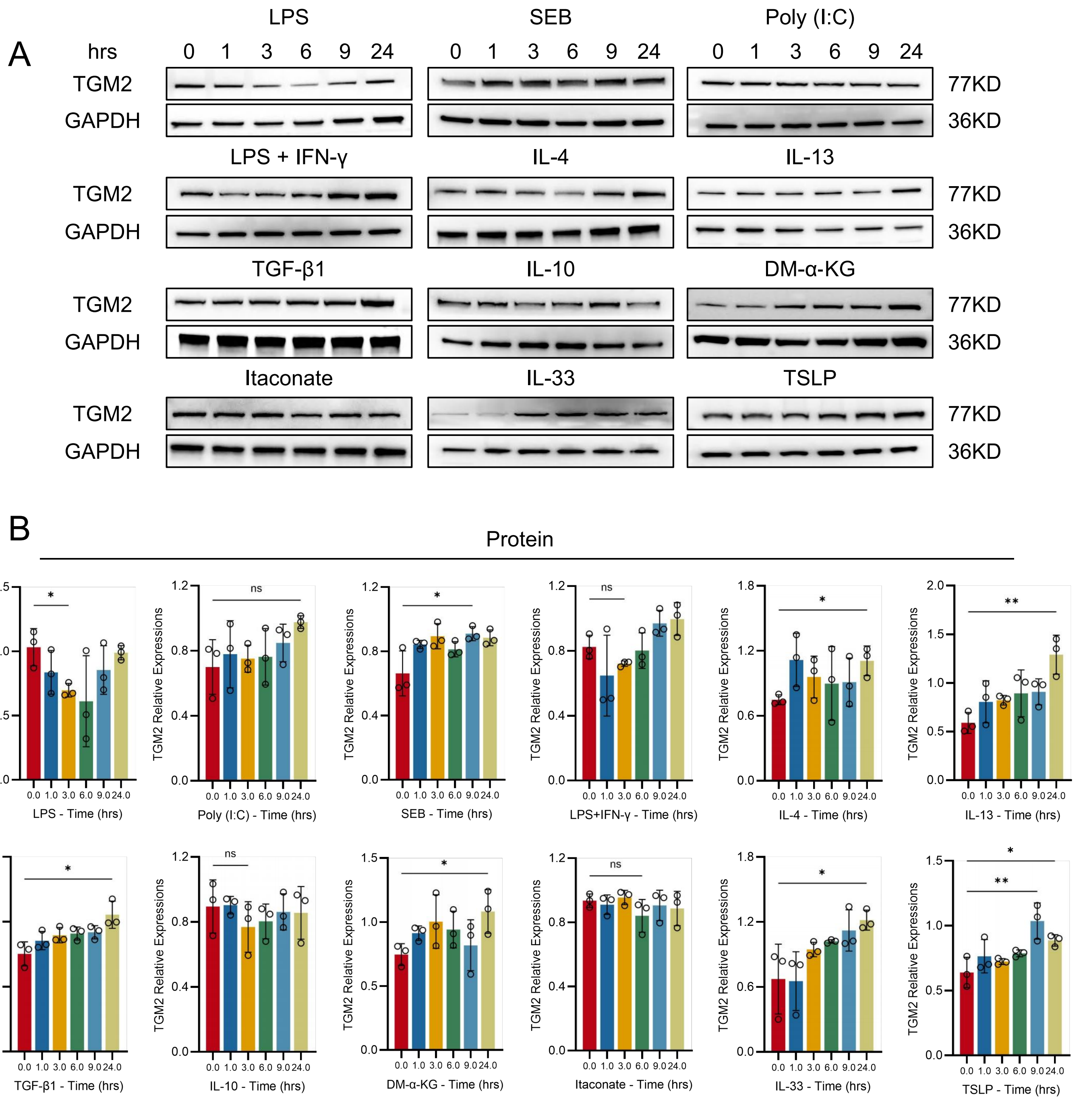
A
LPS
SEB
Poly (I:C)
hrs
0 1 3 6 9 24
TGM2
GAPDH
77KD
36KD
LPS + IFN-γ
IL-4
IL-13
TGF-β1
IL-10
DM-α-KG
Itaconate
IL-33
TSLP
B
Protein
TGM2 Relative Expressions
0.0 1.0 3.0 6.0 9.0 24.0
LPS - Time (hrs)
Poly (I:C) - Time (hrs)
SEB - Time (hrs)
LPS+IFN-γ - Time (hrs)
IL-4 - Time (hrs)
IL-13 - Time (hrs)
TGF-β1 - Time (hrs)
IL-10 - Time (hrs)
DM-α-KG - Time (hrs)
Itaconate - Time (hrs)
IL-33 - Time (hrs)
TSLP - Time (hrs)
ns
*
**

Suppl. Fig. S9

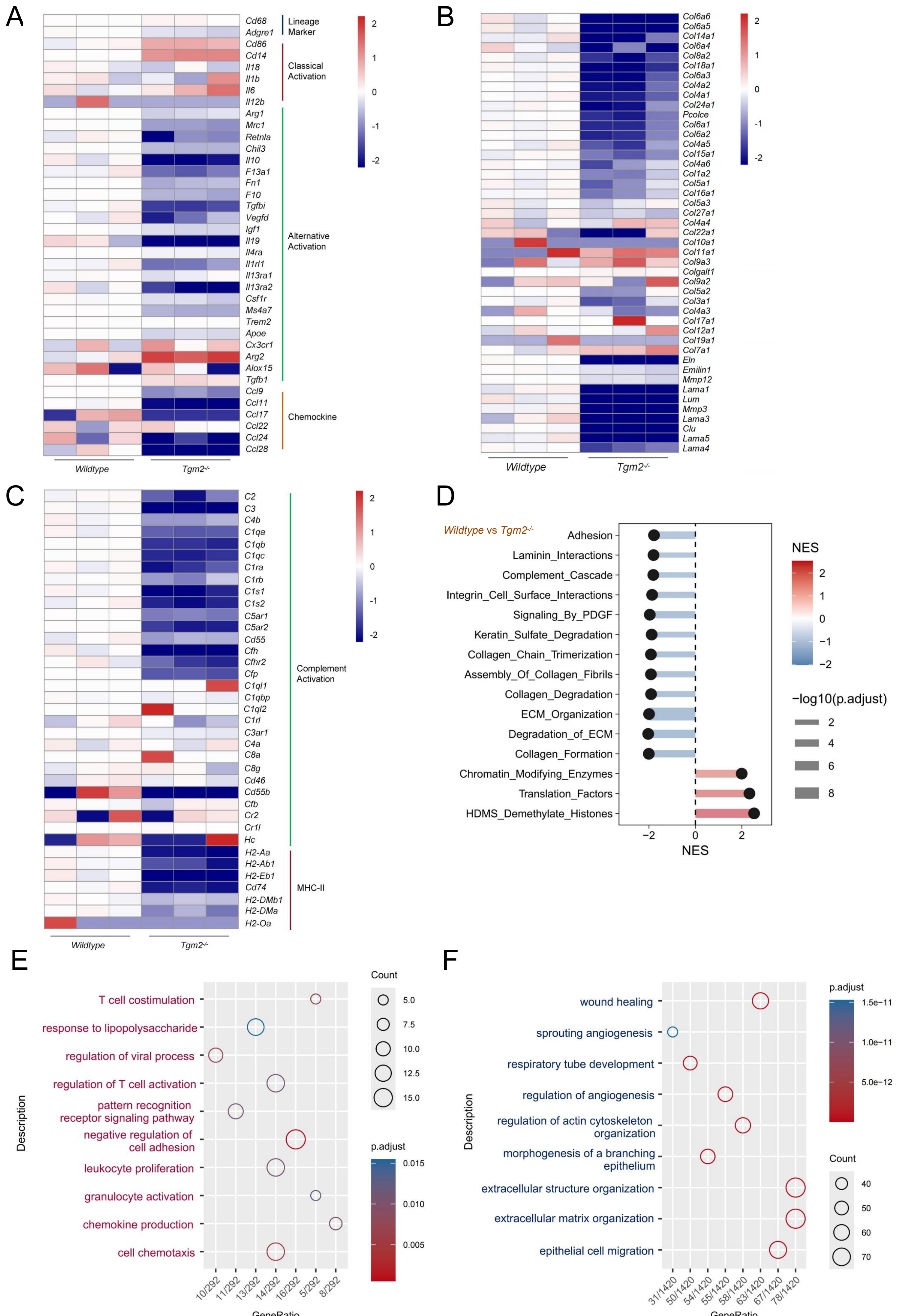

A
Cd68
Adgre1
Cd86
Cd14
Il18
Il1b
Il6
Il12b
Arg1
Mrc1
Retnla
Chil3
Il10
F13a1
Fn1
F10
Tgfbi
Vegfd
Igf1
Il19
Il4ra
Il1rl1
Il13ra1
Il13ra2
Csf1r
Ms4a7
Trem2
Apoe
Cx3cr1
Arg2
Alox15
Tgfb1
Ccl9
Ccl11
Ccl17
Ccl22
Ccl24
Ccl28
Lineage Marker
Classical Activation
Alternative Activation
Chemockine
Wildtype
Tgm2-/-
2
1
0
-1
-2
B
Col6a6
Col6a5
Col14a1
Col6a4
Col8a2
Col18a1
Col6a3
Col4a2
Col4a1
Col24a1
Pcolce
Col6a1
Col6a2
Col4a5
Col15a1
Col4a6
Col1a2
Col5a1
Col16a1
Col5a3
Col27a1
Col4a4
Col22a1
Col10a1
Col11a1
Col9a3
Colgalt1
Col9a2
Col5a2
Col3a1
Col4a3
Col17a1
Col12a1
Col19a1
Col7a1
Eln
Emilin1
Mmp12
Lama1
Lum
Mmp3
Lama3
Clu
Lama5
Lama4
Wildtype
Tgm2-/-
2
1
0
-1
-2
C
C2
C3
C4b
C1qa
C1qb
C1qc
C1ra
C1rb
C1s1
C1s2
C5ar1
C5ar2
Cd55
Cfh
Cfhr2
Cfp
C1ql1
C1qbp
C1ql2
C1rl
C3ar1
C4a
C8a
C8g
Cd46
Cd55b
Cfb
Cr2
Cr1l
Hc
H2-Aa
H2-Ab1
H2-Eb1
Cd74
H2-DMb1
H2-DMa
H2-Oa
Complement Activation
MHC-II
Wildtype
Tgm2-/-
2
1
0
-1
-2
D
Wildtype vs Tgm2-/-
Adhesion
Laminin_Interactions
Complement_Cascade
Integrin_Cell_Surface_Interactions
Signaling_By_PDGF
Keratin_Sulfate_Degradation
Collagen_Chain_Trimerization
Assembly_Of_Collagen_Fibrils
Collagen_Degradation
ECM_Organization
Degradation_of_ECM
Collagen_Formation
Chromatin_Modifying_Enzymes
Translation_Factors
HDMS_Demethylate_Histones
−2
0
2
NES
NES
2
1
0
−1
−2
−log10(p.adjust)
2
4
6
8
E
T cell costimulation
response to lipopolysaccharide
regulation of viral process
regulation of T cell activation
pattern recognition receptor signaling pathway
negative regulation of cell adhesion
leukocyte proliferation
granulocyte activation
chemokine production
cell chemotaxis
Description
10/292
11/292
13/292
14/292
16/292
5/292
8/292
GeneRatio
Count
5.0
7.5
10.0
12.5
15.0
p.adjust
0.015
0.010
0.005
F
wound healing
sprouting angiogenesis
respiratory tube development
regulation of angiogenesis
regulation of actin cytoskeleton organization
morphogenesis of a branching epithelium
extracellular structure organization
extracellular matrix organization
epithelial cell migration
Description
31/1420
50/1420
54/1420
55/1420
58/1420
63/1420
67/1420
78/1420
GeneRatio
p.adjust
1.5e−11
1.0e−11
5.0e−12
Count
40
50
60
70

Suppl. Fig. S10

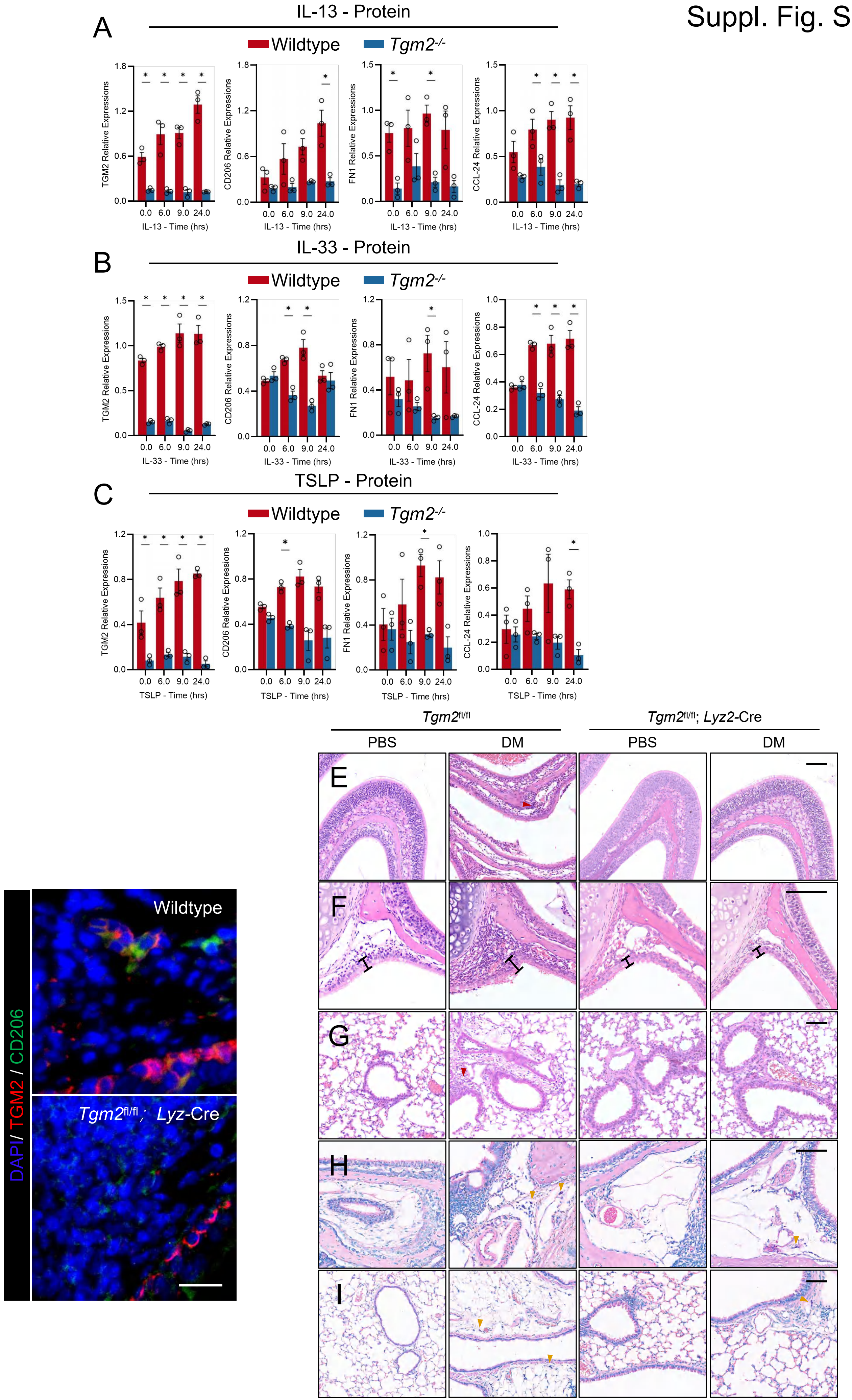

A

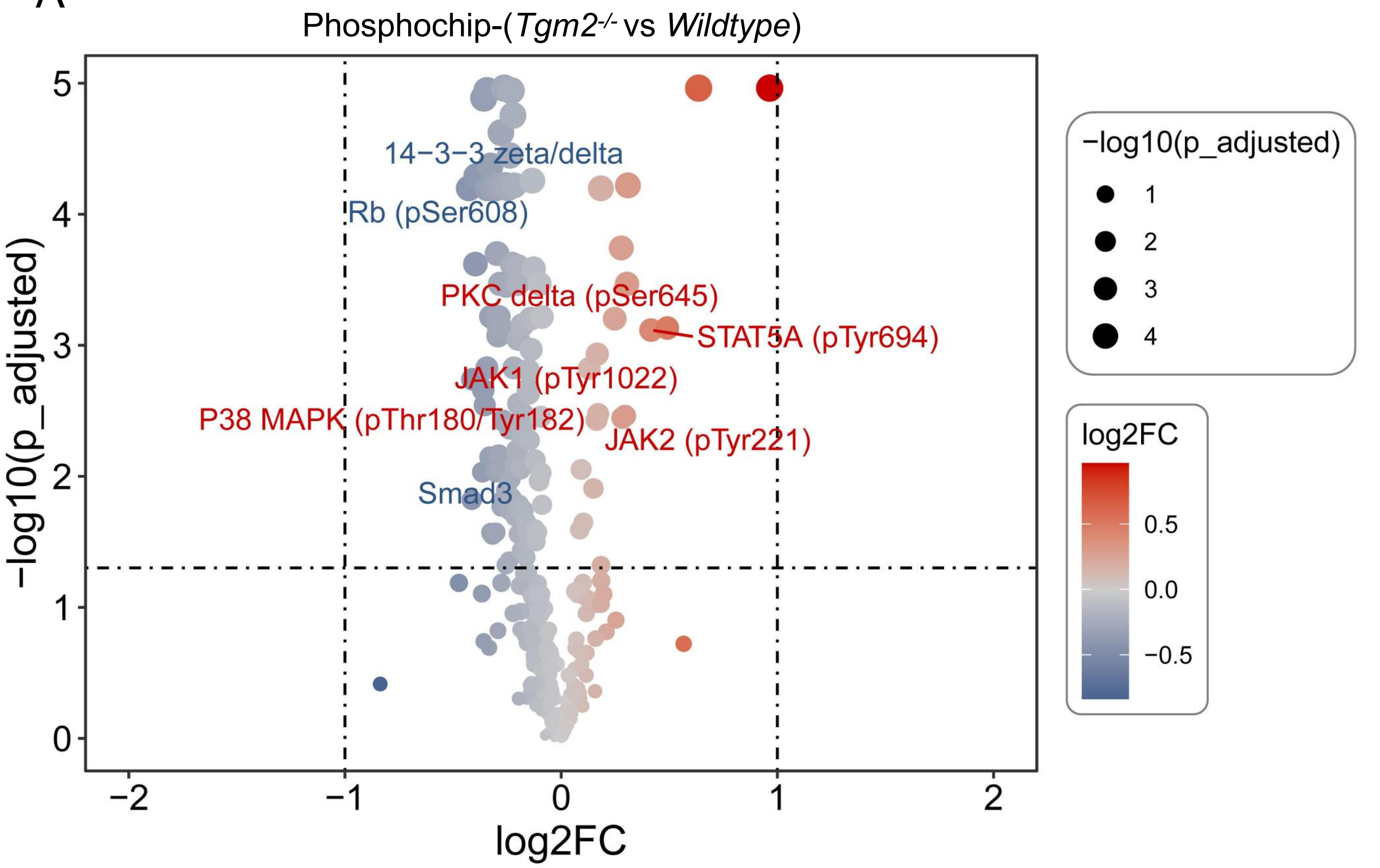
Phosphochip-(Tgm2-/- vs Wildtype)
14−3−3 zeta/delta
Rb (pSer608)
PKC delta (pSer645)
STAT5A (pTyr694)
JAK1 (pTyr1022)
P38 MAPK (pThr180/Tyr182)
JAK2 (pTyr221)
Smad3
−log10(p_adjusted)
log2FC
−log10(p_adjusted)
1
2
3
4
log2FC
0.5
0.0
−0.5

Suppl. Fig. S12

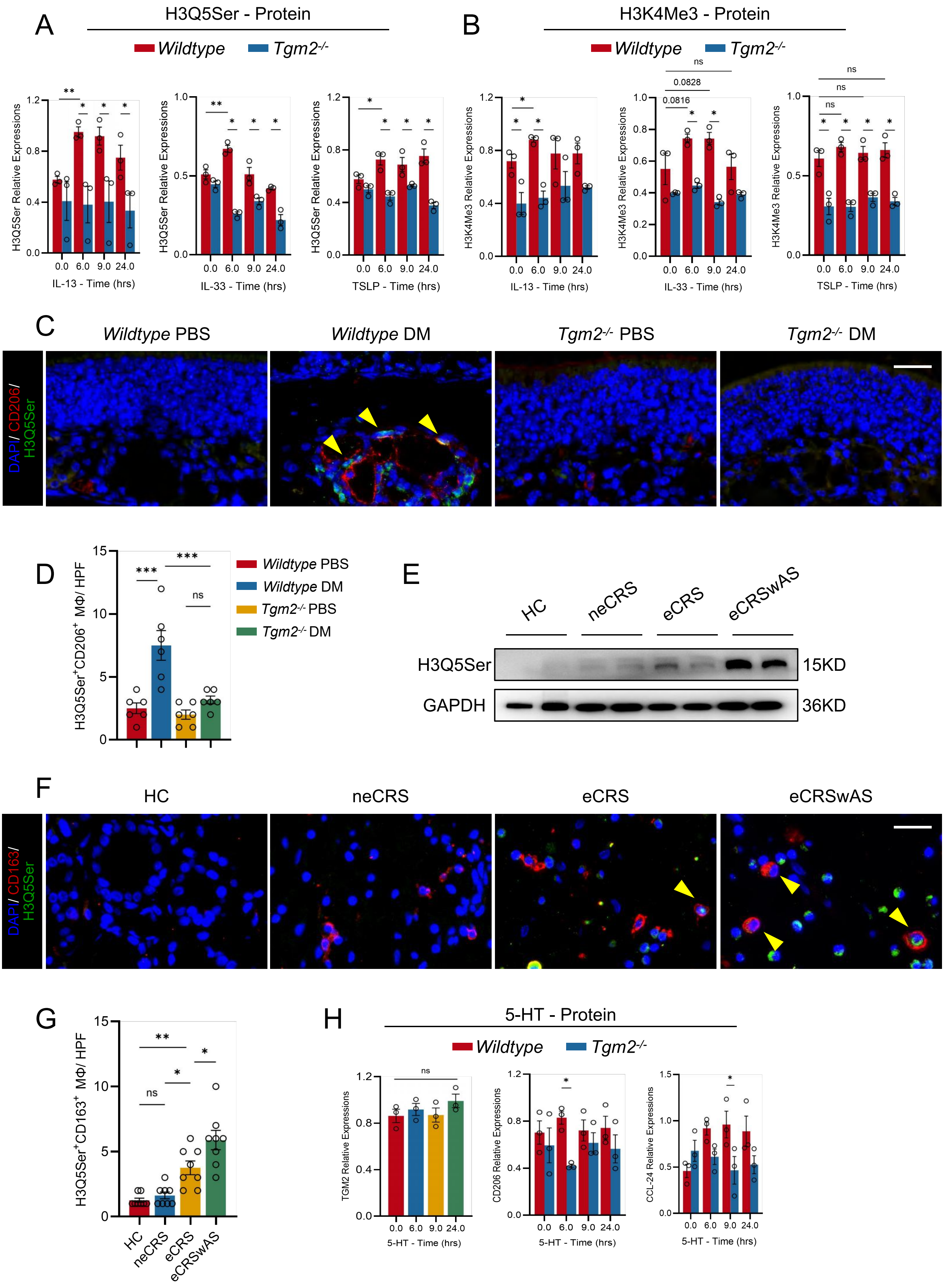

Suppl. Fig. S13

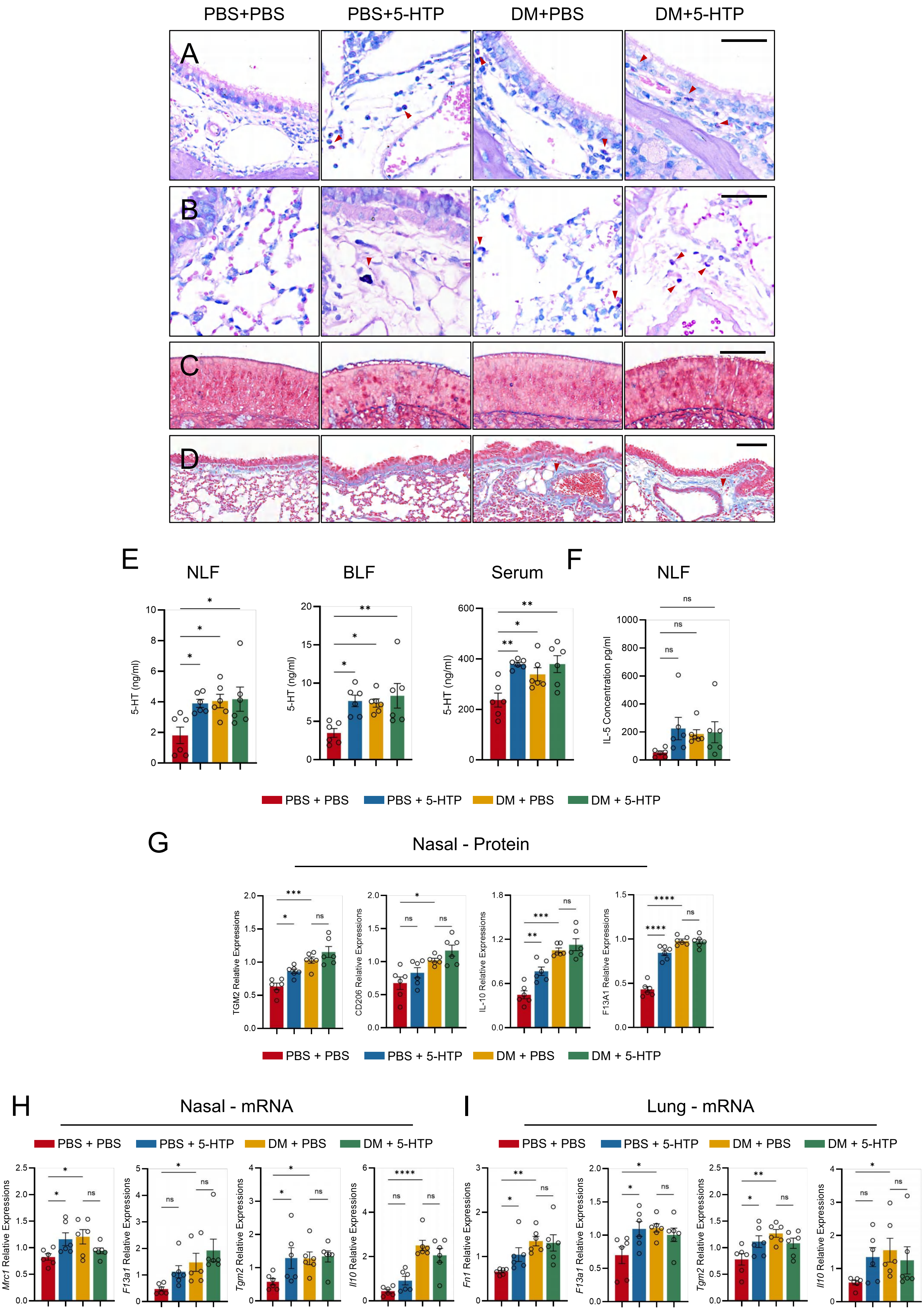

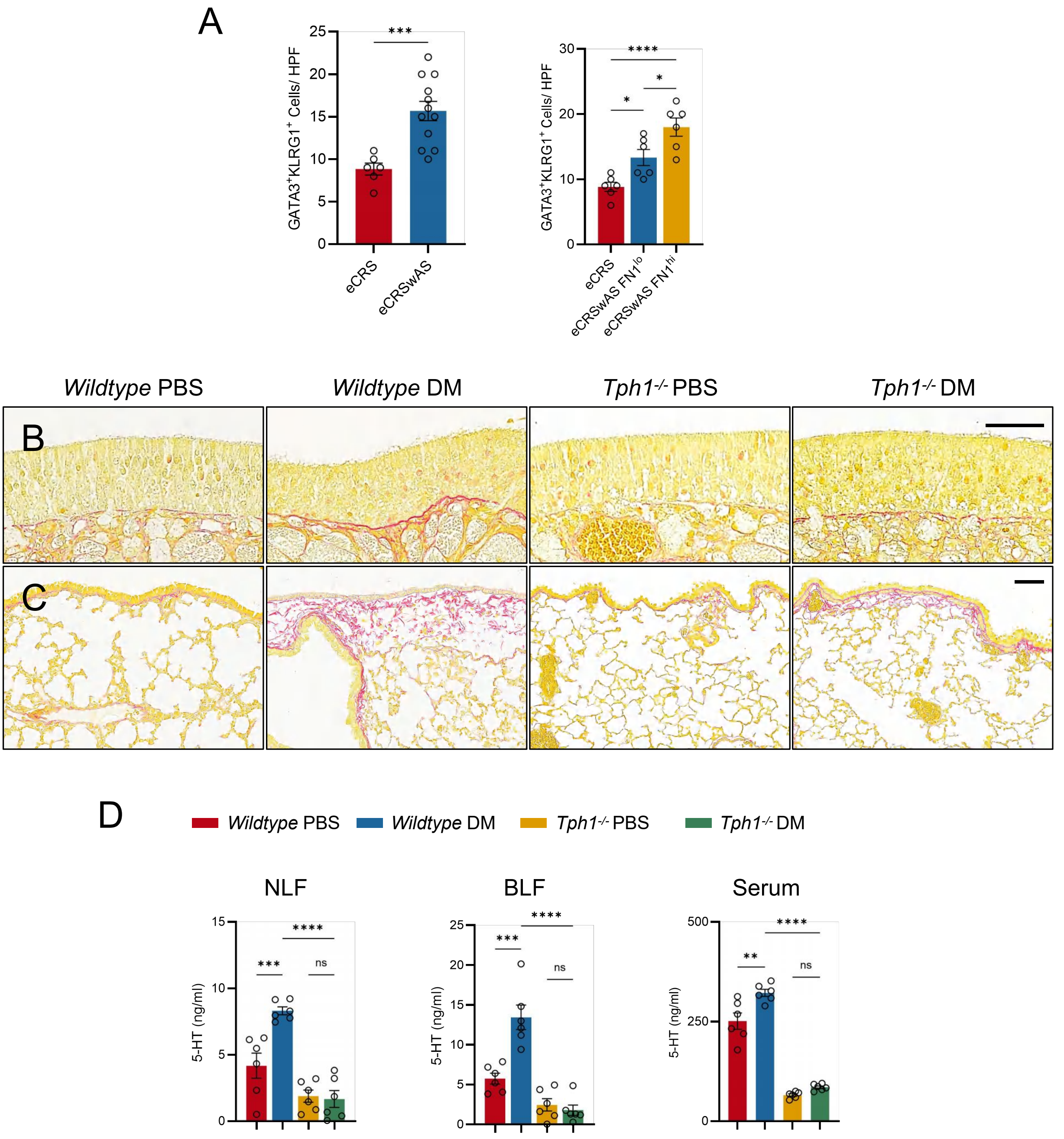
A
GATA3+KLRG1+ Cells/ HPF
***
eCRS
eCRSwAS
****
*
*
eCRS
eCRSwAS FN1lo
eCRSwAS FN1hi
Wildtype PBS
Wildtype DM
Tph1-/- PBS
Tph1-/- DM
B
C
D
Wildtype PBS
Wildtype DM
Tph1-/- PBS
Tph1-/- DM
NLF
BLF
Serum
5-HT (ng/ml)
***
****
**
ns

Suppl. Fig. S15

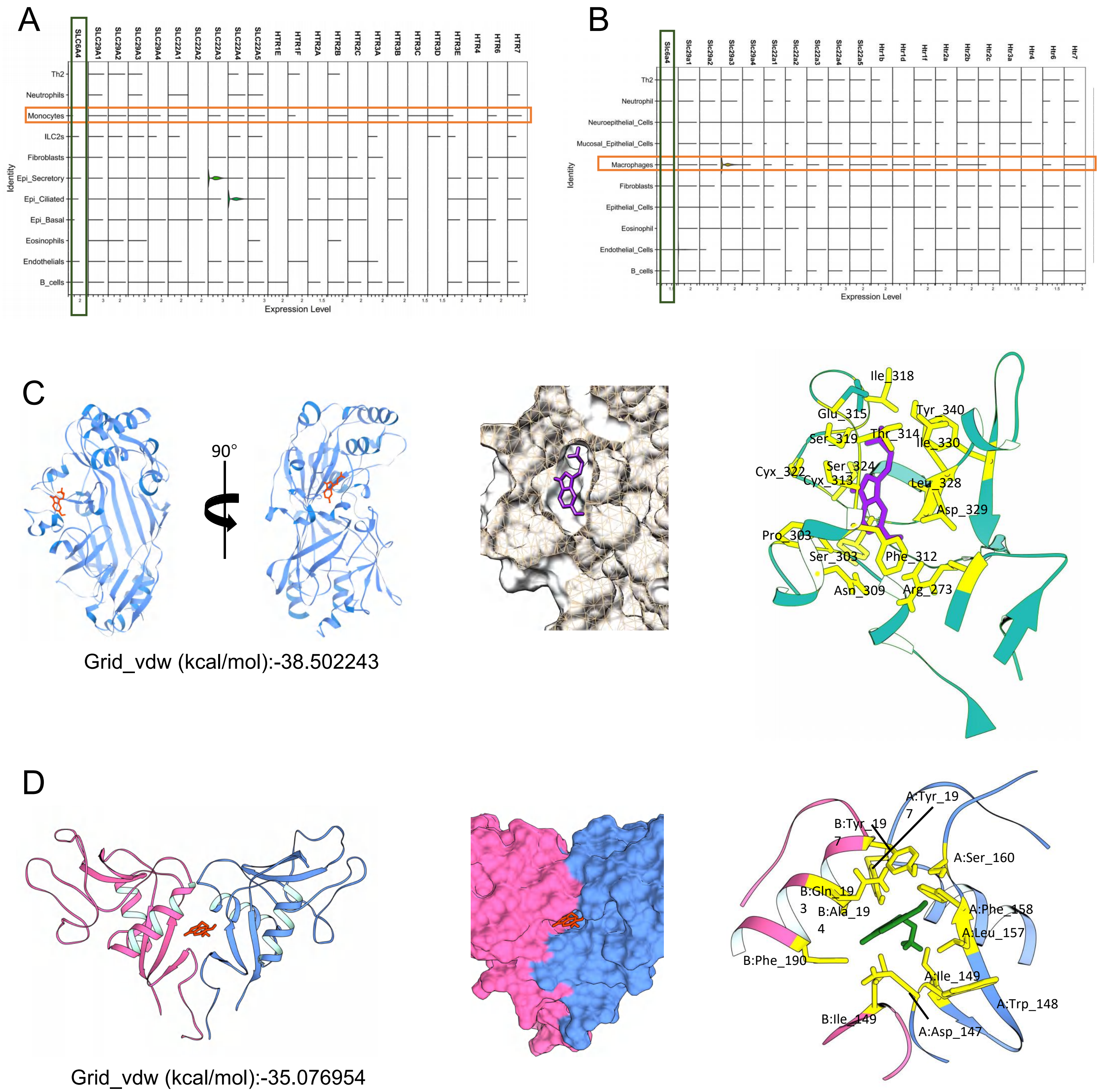

Suppl. Fig. S16

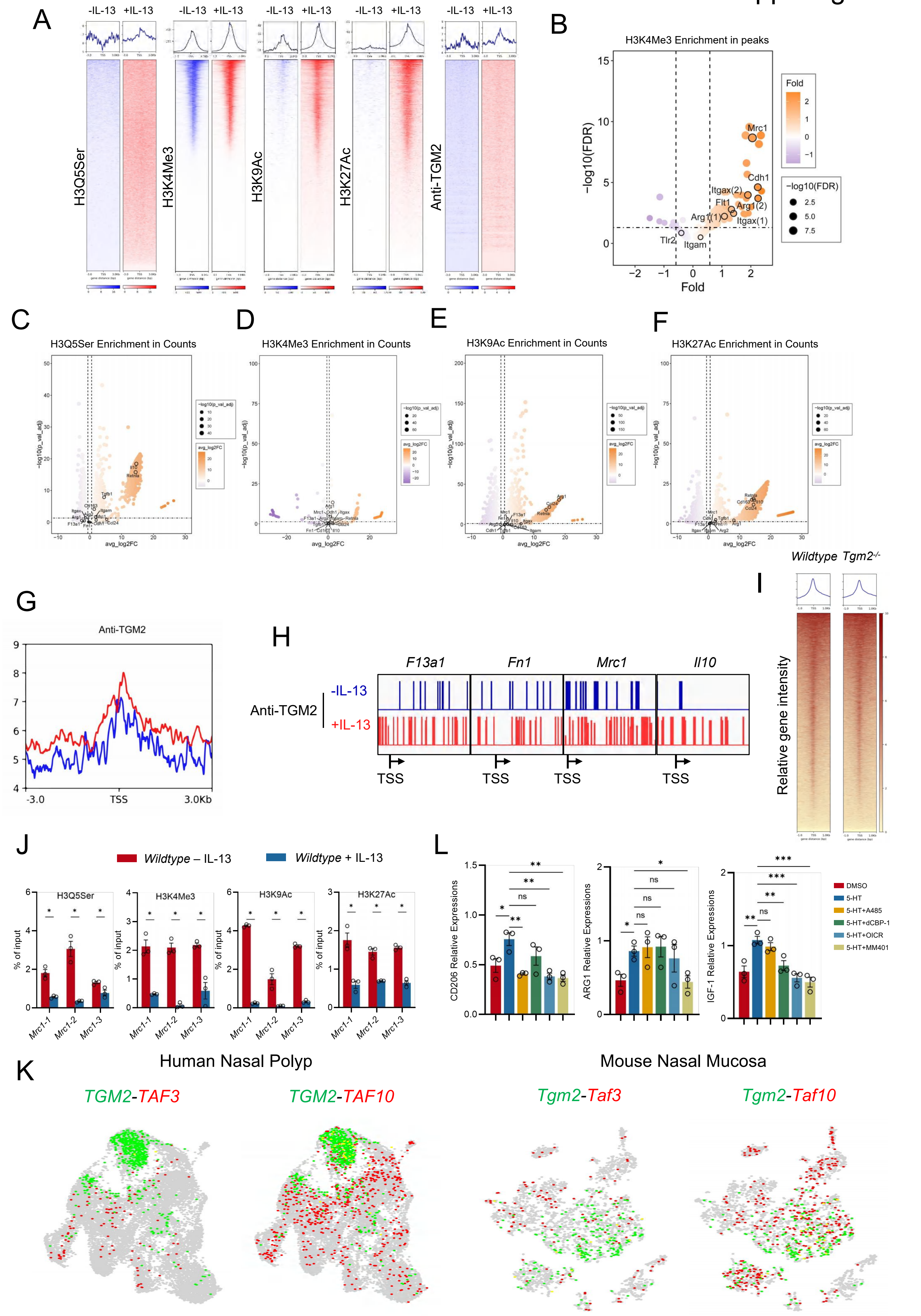

Suppl. Fig. S17

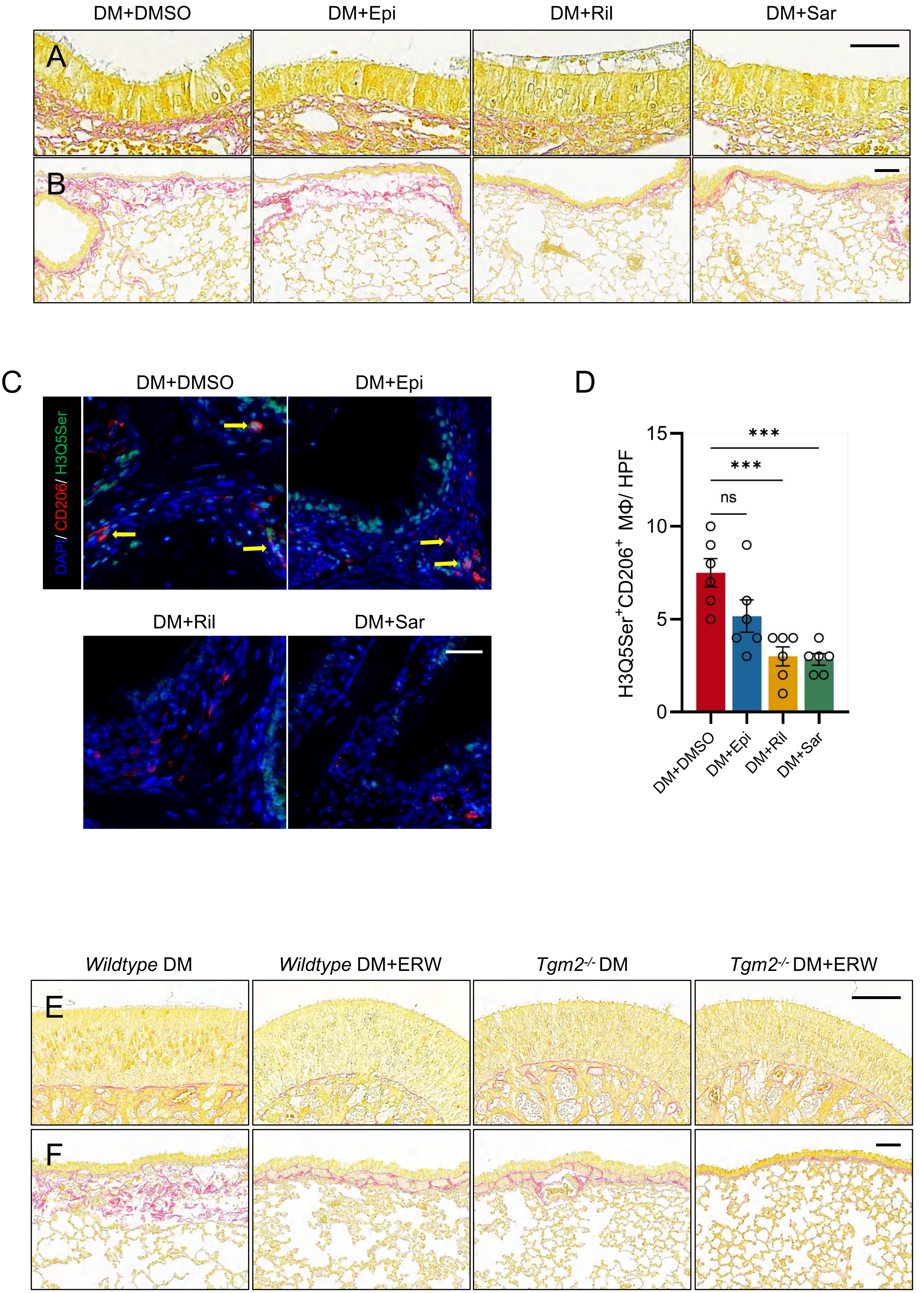

Suppl. Fig. S18

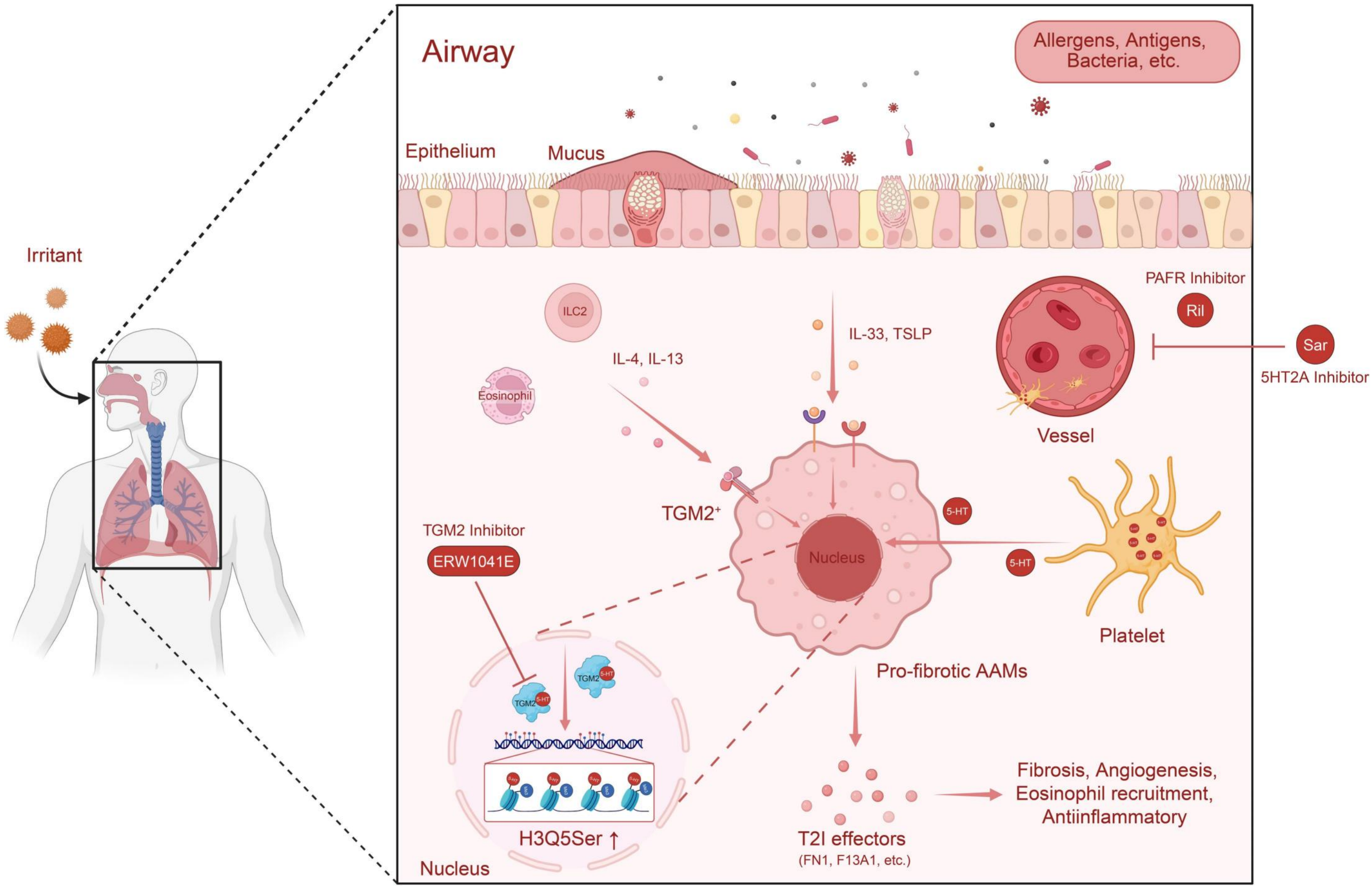

Supplement: Supplementary file 1 [file 41423_2026_1455_MOESM1_ESM.pdf]
